# Draft assemblies for 177 bird species enhance genus-level coverage

**DOI:** 10.1093/gigascience/giag045

**Published:** 2026-05-09

**Authors:** Guangji Chen, Shuang Wang, Daniel Bilyeli Øksnebjerg, Sascha Dreyer Nielsen, Wei Dai, Wei Jiang, Jing Liang, Wei Han, Chengran Zhou, Qiye Li, Bent Petersen, Ara Monadjem, Zamekile D Bhembe, Machawe Maphalala, Diego Ocampo, Luis Sandoval, Jörns Fickel, Alex D Greenwood, Claudia A Szentiks, Marco Roller, Sharon M Birks, Adam D Leaché, Alejandro Rico-Guevara, Jérôme Fuchs, Nguyen Tran Vy, Christina Hvilsom, Juliana Andrea Berner, Jan Terje Lifjeld, Arild Johnsen, Lars Erik Johannessen, Kim Labuschagne, Knud Andreas Jønsson, Martin Irestedt, Andrew Hart Reeve, Leo Joseph, Olof Hellgren, Robb T Brumfield, Theresa M Burg, Juan Carlos Illera, Alexandre Aleixo, Ben Smit, Frank E Rheindt, Jessica Lee, Isao Nishiumi, Javier Quesada, John P Dumbacher, Manuel Schweizer, Michael J Andersen, Christopher C Witt, Richard A Phillips, Richard O Prum, Kristof Zyskowski, Steven M Goodman, Marie Jeanne Raherilalao, Ulf Ottosson, Yahkat Barshep, Sam Ivande, Vojtěch Brlík, Emmanuel Okposio, Bonny Koane, Tri Haryoko, Erich D Jarvis, Carsten Rahbek, Fumin Lei, Gary R Graves, Shaohong Feng, Peter A Hosner, M Thomas P Gilbert, Guojie Zhang

**Affiliations:** Center for Evolutionary & Organismal Biology, Liangzhu Laboratory & Women’s Hospital, Zhejiang University School of Medicine, Hangzhou 310058, China; Center for Evolutionary & Organismal Biology, Liangzhu Laboratory & Women’s Hospital, Zhejiang University School of Medicine, Hangzhou 310058, China; Center for Evolutionary Hologenomics, Globe Institute, University of Copenhagen, 1353 Copenhagen K, Denmark; Center for Evolutionary Hologenomics, Globe Institute, University of Copenhagen, 1353 Copenhagen K, Denmark; BGI Research, Wuhan 430074, China; State Key Laboratory of Genome and Multi-omics Technologies & Shenzhen Key Laboratory of Forensics, BGI Research, Shenzhen 518083, China; Center for Evolutionary & Organismal Biology, Liangzhu Laboratory & Women’s Hospital, Zhejiang University School of Medicine, Hangzhou 310058, China; Center for Evolutionary & Organismal Biology, Liangzhu Laboratory & Women’s Hospital, Zhejiang University School of Medicine, Hangzhou 310058, China; BGI Research, Wuhan 430074, China; State Key Laboratory of Genome and Multi-omics Technologies & Shenzhen Key Laboratory of Forensics, BGI Research, Shenzhen 518083, China; College of Life Sciences, University of Chinese Academy of Sciences, Beijing 100049, China; Center for Evolutionary Hologenomics, Globe Institute, University of Copenhagen, 1353 Copenhagen K, Denmark; Centre of Excellence for Omics-Driven Computational Biodiscovery (COMBio), Faculty of Applied Sciences, AIMST University, Kedah, Malaysia; Department of Zoology and Entomology, Mammal Research Institute, University of Pretoria, Hatfield 0028, Pretoria, South Africa; Department of Biological Sciences, University of Eswatini, Lozitha road, Kwaluseni M201, Eswatini; Department of Biological Sciences, University of Eswatini, Lozitha road, Kwaluseni M201, Eswatini; Department of Biological Sciences, University of Eswatini, Lozitha road, Kwaluseni M201, Eswatini; Museum of Vertebrate Zoology, University of California Berkeley, Berkeley, CA 94720, USA; Centro de Investigación en Biodiversidad y Ecología Tropical, Universidad de Costa Rica, San José 11501-2060, Costa Rica; Centro de Investigación en Biodiversidad y Ecología Tropical, Universidad de Costa Rica, San José 11501-2060, Costa Rica; Laboratorio de Ecología Urbana y Comunicación Animal, Escuela de Biología, Universidad de Costa Rica, San José 11501-2060, Costa Rica; Department of Evolutionary Genetics, Leibniz Institute for Zoo and Wildlife Research (IZW), Alfred-Kowalke-Strasse 17, 10315 Berlin, Germany; Institute of Biochemistry and Biology, Potsdam University, Karl-Liebknecht-Straße 24-25, 14476 Potsdam, Germany; Department Wildlife Diseases, Leibniz Institute for Zoo and Wildlife Research, Alfred-Kowalke-Strasse 17, 10315 Berlin, Germany; Department of Veterinary Medicine, Freie Universität Berlin, 14163 Berlin, Germany; Department Wildlife Diseases, Leibniz Institute for Zoo and Wildlife Research, Alfred-Kowalke-Strasse 17, 10315 Berlin, Germany; Wilhelma Zoological-Botanical Gardens Stuttgart, Wilhelma 13, D-70376 Stuttgart, Germany; Department of Animal Sciences, Division of Microbiology and Animal Hygiene, Faculty of Agricultural Science, Georg-August-University, Burckhardtweg 2, D-37077 Göttingen, Germany; Institute for Microbiology, University of Veterinary Medicine Hannover, Foundation, Bischofsholer Damm15, D-30173 Hannover, Germany; Burke Museum of Natural History and Culture, University of Washington, Seattle, WA 98105, USA; Burke Museum of Natural History and Culture, University of Washington, Seattle, WA 98105, USA; Department of Biology, University of Washington, Seattle, WA 98105, USA; Burke Museum of Natural History and Culture, University of Washington, Seattle, WA 98105, USA; Department of Biology, University of Washington, Seattle, WA 98105, USA; Institut de Systématique, Evolution, Biodiversité (ISYEB), Muséum national d’Histoire naturelle, CNRS, SU, EPHE-PSL, UA, CP51, 57 Rue Cuvier, 75005 Paris, France; Institute of Tropical Biology, VAST, Ho Chi Minh City 700000, Vietnam; Copenhagen Zoo, 2000 Frederiksberg, Denmark; Copenhagen Zoo, 2000 Frederiksberg, Denmark; Natural History Museum, University of Oslo, 0562 Oslo, Norway; Natural History Museum, University of Oslo, 0562 Oslo, Norway; Natural History Museum, University of Oslo, 0562 Oslo, Norway; South African National Biodiversity Institute(SANBI), Pretoria 0184, South Africa; Natural History Museum of Denmark, University of Copenhagen, 2100 Copenhagen, Denmark; Department of Bioinformatics and Genetics, Swedish Museum of Natural History, Box 50007, SE-104 05 Stockholm, Sweden; Department of Bioinformatics and Genetics, Swedish Museum of Natural History, Box 50007, SE-104 05 Stockholm, Sweden; Natural History Museum of Denmark, University of Copenhagen, 2100 Copenhagen, Denmark; Department of Bioinformatics and Genetics, Swedish Museum of Natural History, Box 50007, SE-104 05 Stockholm, Sweden; Australian National Wildlife Collection, National Research Collections Australia, CSIRO, Canberra, Canberra, ACT 2601, Australia; Department of Biology, Lund University, Sölvegatan 35, 223 62 Lund, Sweden; Museum of Natural Science, Louisiana State University, Baton Rouge, LA 70803, USA; Department of Biological Sciences, Louisiana State University, Baton Rouge, LA 70803, USA; Department of Biological Sciences, University of Lethbridge, Lethbridge, AB, T1K 3M4, Canada; Biodiversity Research Institute (CSIC-Oviedo University-Principality of Asturias), University of Oviedo, Campus of Mieres, E-33006 Mieres, Asturias, Spain; Museu Paraense Emílio Goeldi, Avenida Magalhães Barata, 376, Belém-PA, 66040-170, PA, Brazil; Vale Institute of Technology, Rua Boaventura da Silva 955, Belém, PA, 66055-090, Brazil; Department of Zoology and Entomology, Rhodes University, Makhanda 6140, South Africa; Department of Biological Sciences, National University of Singapore, 14 Science Drive 4, Singapore 117543; Mandai Nature, 80 Mandai Lake Road, Mandai, Singapore 729826; Department of Zoology, National Museum of Nature and Science, Tsukuba, Ibaraki, 305-0005, Japan; Department of Vertebrates, Natural Sciences Museum of Barcelona, Plaça Leonardo da Vinci 4-5, 08003 Barcelona, Spain; Ornithology and Mammalogy Department, California Academy of Sciences, San Francisco, CA 94118, USA; Natural History Museum Bern, Bernastrasse 15, 3005 Bern, Switzerland; Division of Population Genetics, Institute of Ecology and Evolution, University of Bern, Baltzerstrasse 6, 3012 Bern, Switzerland; Museum of Southwestern Biology, University of New Mexico, Albuquerque, NM 87131, USA; Museum of Southwestern Biology, University of New Mexico, Albuquerque, NM 87131, USA; British Antarctic Survey (BAS), Natural Environment Research Council (NERC), High Cross, Madingley Road, CB3 0ET Cambridge, UK; Department of Ecology and Evolutionary Biology, and Yale Peabody Museum, Yale University, 170 Whitney Avenue, New Haven, CT 06511, USA; Division of Vertebrate Zoology, Peabody Museum of Natural History, Yale University, New Haven, CT 06520-8106, USA; Field Museum of Natural History, 1400 South Lake Shore Drive, Chicago, IL 60605, USA; Association Vahatra, BP 3972, Antananarivo 101, Madagascar; Mention Zoologie et Biodiversité Animale, Université d’Antananarivo, BP 906, Antananarivo 101, Madagascar; A.P. Leventis Ornithological Research Institute (APLORI), Centre of Excellence, University of Jos, P.O. Box 13404, Laminga, Jos-East, Plateau State, Nigeria; A.P. Leventis Ornithological Research Institute (APLORI), Centre of Excellence, University of Jos, P.O. Box 13404, Laminga, Jos-East, Plateau State, Nigeria; Department of Zoology, University of Jos, P.O. Box 13404, Laminga, Jos-East, Plateau State, Nigeria; A.P. Leventis Ornithological Research Institute (APLORI), Centre of Excellence, University of Jos, P.O. Box 13404, Laminga, Jos-East, Plateau State, Nigeria; Global Center for Species Survival, Indianapolis Zoo, 1200 West Washington St. Indianapolis, IN 46222, USA; Department of Ecology, Charles University, 12800 Prague, Czech Republic; Department of Biology, California State University, Fresno, CA 93740, USA; New Guinea Binatang Research Centre, P.O. Box 604, Madang 511, Papua New Guinea; Museum Zoologicum Bogoriense, Research Center for Biosystematics and Evolution, National Research and Innovation Agency (BRIN), Cibinong 16911, West Java, Indonesia; The Vertebrate Genome Laboratory, The Rockefeller University, 1230 York Avenue, New York, NY 10065, USA; Center for Macroecology, Evolution and Climate, Globe Institute, University of Copenhagen, Universitetsparken 15, 2100 Copenhagen Ø, Denmark; Center for Global Mountain Biodiversity, Globe Institute, University of Copenhagen, Universitetsparken 15, 2100 Copenhagen Ø, Denmark; Department of Biology, University of Southern Denmark, Campusvej 55, 5230 Odense M, Denmark; Institute of Zoology, Chinese Academy of Sciences, Beijing 100101, China; Center for Macroecology, Evolution and Climate, Globe Institute, University of Copenhagen, Universitetsparken 15, 2100 Copenhagen Ø, Denmark; Department of Vertebrate Zoology, National Museum of Natural History, Smithsonian Institution, Washington, DC 20013-7012, USA; Center for Evolutionary & Organismal Biology, Liangzhu Laboratory & Women’s Hospital, Zhejiang University School of Medicine, Hangzhou 310058, China; Department of General Surgery of Sir Run Run Shaw Hospital, Zhejiang University School of Medicine, Hangzhou 310016, China; Natural History Museum of Denmark, University of Copenhagen, 2100 Copenhagen, Denmark; Center for Macroecology, Evolution and Climate, Globe Institute, University of Copenhagen, Universitetsparken 15, 2100 Copenhagen Ø, Denmark; Center for Global Mountain Biodiversity, Globe Institute, University of Copenhagen, Universitetsparken 15, 2100 Copenhagen Ø, Denmark; Center for Evolutionary Hologenomics, Globe Institute, University of Copenhagen, 1353 Copenhagen K, Denmark; University Museum, NTNU, 7491 Trondheim, Norway; Center for Evolutionary & Organismal Biology, Liangzhu Laboratory & Women’s Hospital, Zhejiang University School of Medicine, Hangzhou 310058, China

**Keywords:** birds, genome sequencing, biodiversity

## Abstract

**Background:**

With over 10,000 recognized species, birds constitute one of the most diverse and widely distributed vertebrate groups. Although avian genomics has advanced rapidly over the past decade, substantial gaps remain across the global avifauna. Filling these gaps is essential for understanding macroevolutionary patterns, population structure, and the molecular basis of ecological and behavioral diversity. Worldwide museum collections represent invaluable resources for filling these gaps, yet the typically degraded DNA and limited quantities from historical specimens have posed significant challenges for generating high-quality genome assemblies.

**Results:**

Here, the Bird Genome 10 K Project adopted low-input sequencing strategies that reduce costs while improving assembly quality compared with earlier order- and family-level genomes. Using mainly stLFR, complemented by 10X Genomics and standard next-generation sequencing, we assembled 177 avian genomes from museum specimens and tissue collections representing 161 genera, including 102 newly sequenced at the genomic level. The assemblies average ∼1.2 Gb in size, with scaffold N50 = 8.03 Mb, contig N50 = 120 kb, 93% BUSCO completeness, and Merqury Quality Value score of 56.

**Conclusions:**

These genomes greatly expand avian taxonomic coverage and demonstrate the efficiency of low-input sequencing for generating high-quality assemblies from limited and often degraded material sourced from museum specimens. This resource provides a foundation for comparative genomics, conservation genetics, and evolutionary studies across the avian tree of life.

## Context

**Figure 1:**
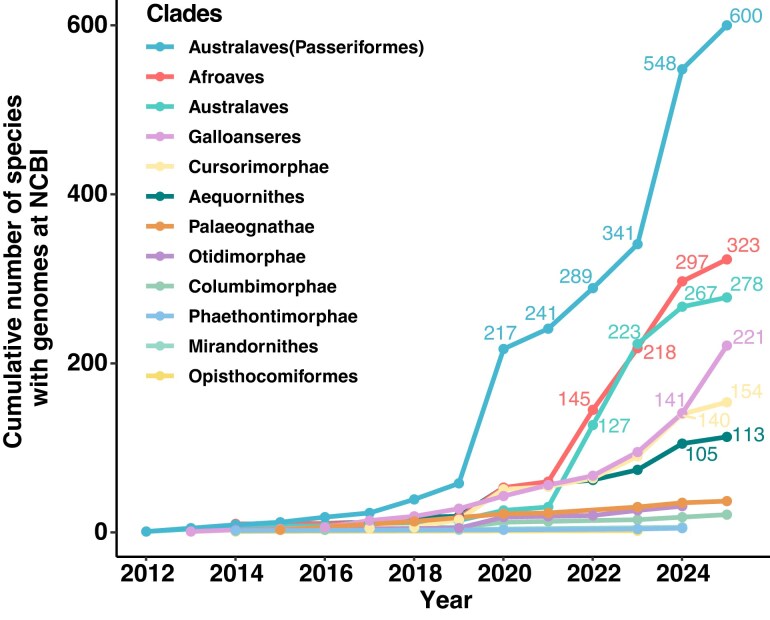
Temporal accumulation of avian reference genomes in the NCBI database. Cumulative number of bird species with reference genomes in the NCBI database (obtained from [27] on 11 October 2025). Data points are color-coded by taxonomic clades to illustrate the expanding genomic representation across different avian lineages.

Birds, derived from theropod dinosaurs, constitute one of the most species-rich and widely distributed vertebrate radiations, encompassing more than 10,000 species and over 2,000 genera that inhabit nearly every ecosystem on Earth [1, 2]. Over the past decade, facilitated by international efforts such as the Bird 10,000 Genomes (B10K) Project [3, 4] and the Vertebrate Genomes Project, avian genome resources have expanded rapidly (Fig. 1), providing unprecedented opportunities for comparative and functional genomics. This growth has been enabled not only by advances in sequencing and assembly technologies but also by the invaluable contribution of natural history museums and institutional biobanks, whose curated specimens have become indispensable for large-scale genome initiatives.

The B10K Project has achieved significant milestones over the past decade in generating genomic data for species representing order- and family-level diversity across the avian tree of life [3–6]. However, genus- and species-level coverage remains highly uneven. Several genera, such as *Aphelocoma, Falco, Gallus, Anas*, and *Haemorhous*, are represented by dozens of assemblies, whereas many lineages remain underrepresented due to limited access to fresh tissues or vouchered samples. For numerous species, especially those inhabiting remote regions or represented primarily by historical specimens, natural history collections remain the only feasible sources of DNA. However, these collections are often geographically dispersed and difficult to access for genomic research due to limitations in both the quality (high-molecular-weight genomic DNA) and quantity of available genetic material. As a result, molecular-level understanding of these taxa has long been constrained by the scarcity of genomic resources.

**Figure 2:**
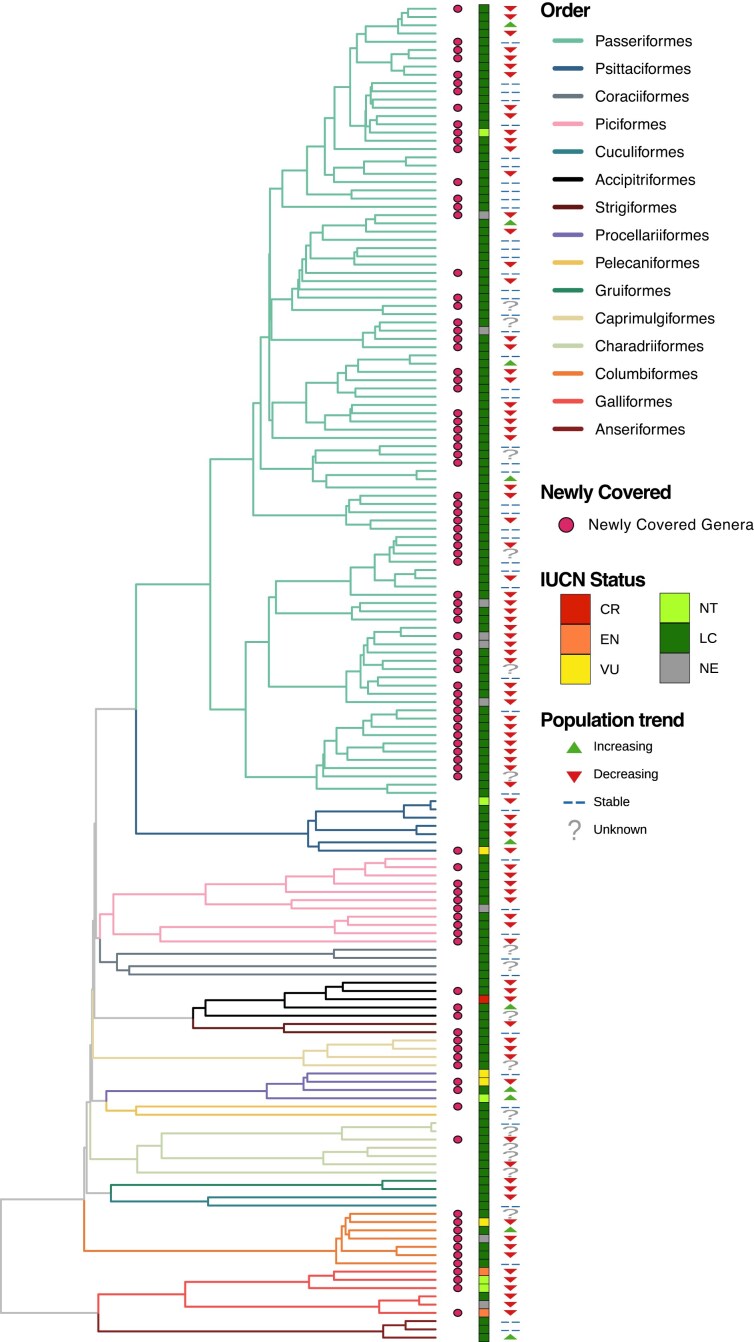
Phylogenetic distribution, taxonomic representation, and conservation profiles of the 177 newly sequenced avian genomes. Branches and internal clades are color-coded according to taxonomic orders. Red circles at the tips indicate newly covered genera. The color-coded strip represents the IUCN Red List categories (CR: critically endangered; EN: endangered; VU: vulnerable; NT: near threatened; LC: least concern; NE: not evaluated; obtained from [28]). And the symbols indicate the population trend, including increasing, decreasing, stable, and unknown.

Recent advances in low-input sequencing and museomics technologies (e.g., stLFR [7]) are gradually bridging this gap, allowing for the assembly of genomes from previously inaccessible samples. B10K Project has strategically adopted these low-input sequencing methods, reducing costs while improving genome assembly quality compared to earlier order- and family-level studies. Here, using stLFR technologies, we report draft genome assemblies for 177 bird species representing 161 genera, including 102 genera newly covered at the genomic level (Fig. 2). These newly sequenced genera increased by 4.43% the genus-level coverage of extant bird species, providing a more continuous phylogenetic framework for comparative genomics across the avian tree of life. These assemblies substantially enrich the genomic resources available for avian research, offering new insights into the genetic diversity and evolutionary history of this remarkable vertebrate group.

## Materials and methods

### Sampling, sequencing, assembly, and annotation

#### DNA extraction

Genomic DNA was extracted from 177 avian species, comprising both frozen materials and historical museum specimens, as detailed in Supplementary Table 1. Specifically, 80.8% of the samples (*n* = 143) were collected before 2020 with many challenging historical sources that necessitated the low-input sequencing strategy. All DNA extractions were performed using the “MagMAX DNA Multi-Sample Ultra 2.0” kit (Thermo Fisher) following the manufacturer’s guidelines. For post-lysis, RNA degradation was performed by incubating the lysate with RNase A at 37°C for 5 min. Subsequently, genomic DNA was isolated using a bead-based purification method, utilizing 440 µl of DNA Binding Bead Mix per sample. The automated isolation process was executed on the Kingfisher Duo Prime system (Thermo Fisher Scientific), following the standard MMX_Ultra2_Cell_Tissue_96_Duo protocol. Quality control was performed on the 2200 Tapestation System, and all DNA extractions were stored at −80 °C until library construction.

#### Library construction and sequencing

##### Linked-reads library

The selection of sequencing technologies was strategically determined by the interplay between DNA integrity and the progressive availability of sequencing platforms throughout the project. The majority of the DNA samples (*n* = 161) were prepared using the stLFR (Single-Tube Long Fragment Reads) technology, which served as our primary pipeline due to its ability to generate high-quality linked-read data with 10 ng of input DNA with fragment sizes exceeding 10 kb. During the early phase of this study, 13 DNA samples that exhibited exceptional DNA integrity (main fragment sizes > 40 kb) were prepared using the 10X Genomics Chromium library system.

For the 13 samples prepared using the 10X Genomics Chromium library system, high-molecular-weight genomic DNA was processed with the Chromium Genome Library Kit (10X Genomics, Pleasanton, USA) to generate barcoded linked-read libraries. To ensure compatibility and sequencing efficiency across platforms, the libraries were adapted for sequencing on the BGISEQ-500 platform (BGI-Shenzhen, China) following in-house optimization to produce 150 bp paired-end reads in a total of 1.6 Tb raw sequencing reads (on average ∼103x).

For the 161 samples prepared using stLFR technology (MGI), high-molecular-weight genomic DNA was processed following the manufacturer’s barcoding protocol, which enables the physical linkage of short reads derived from the same long DNA fragment. The barcoded libraries were sequenced on the DNBSEQ-T7 platform (MGI, Shenzhen, China) with PE100+42 mode, which included two 100 bp reads for the genomic inserts and a dedicated 42 bp read to capture the co-barcoding sequences essential for the stLFR method [7], and produced in total 26.3 Tb raw sequencing reads (on average ∼136x).

##### Standard next-generation sequencing

For the remaining 3 samples with relative DNA degradation (fragment sizes <10 kb) that failed to meet the requirements for linked-read library construction, standard short-read next-generation technology with ∼500 ng input DNA was employed to ensure sufficient genomic coverage despite suboptimal sample quality. Sequencing libraries with an average insert size of ∼350 bp were constructed according to the manufacturer’s protocol and sequenced on the DNBSEQ-T1 platform (BGI-Shenzhen, China) to produce 150 bp paired-end reads in total 226 Gb raw sequencing reads (on average 63x).

#### Assembly and statistics of genomes

##### stLFR and 10X Genomics

For the 161 samples sequenced using stLFR technology, raw data were initially transformed into 10X Genomics linked-reads format following the stlfr2supernova pipeline [8]. Subsequently, these 161 samples, together with 13 samples sequenced by 10X Genomics linked-reads technology, were preprocessed with SOAPfilter (v2.2, RRID:SCR_000689) using parameters “-i $insertsize -p -z -g 1 -M 2 -Q 10 lane.lst stat_file” to remove adapters and low-quality reads. The cleaned reads were then introduced into the Supernova software (v2.0.1; RRID:SCR_016756) [9] to assemble the genomes under the “pseudohap” mode. The assembly was executed with the command: “supernova run –id=ID –fastqs=./–localcores=32 –localmem=300 G –nopreflight,” followed by “supernova mkoutput –style=pseudohap –asmdir=./ID/outs/assembly –outprefix=output” to generate the final genomic sequences.

To ensure assembly quality, scaffolds containing more than 80% “N” bases were removed using housed Perl script, which typically resulted in the exclusion of ∼2 scaffolds per assembly. Subsequently, gap filling was applied with Gapcloser (v1.12, RRID:SCR_015026) [10] by using the default parameters and the cleaned paired-end reads.

##### Standard next-generation technology

For the 3 samples sequenced by the standard next-generation technology, reads were cleaned by SOAPfilter (v2.2, RRID:SCR_000689) with previous parameters and assembled using the SOAPdenovo (v2.04, RRID:SCR_010752) [10] with a *K*-mer size of 23-mer following the B10K family-phased assembly strategy [4]. After removing scaffolds with “N” > 80%, GapCloser (v1.12, RRID:SCR_015026) [10] was used to close the intra-scaffold gaps with default parameters based on the cleaned paired-end reads.

#### Gene structures annotation

Annotation of protein-coding genes was conducted with a homology-based method for the 177 bird species following the pipeline implemented by the B10K consortium [4]. The reference gene set consists of the primary reference gene set (20,194 genes), the supplemental human gene set (20,169 genes), and the supplemental transcriptome gene set (5,257 transcripts), which could be accessed [11]. The protein sequences in the primary reference gene set were first aligned to each genome using TBLASTN (v2.2.26; RRID:SCR_011822) [12] with an *e*-value cut-off 1e-5, and multiple adjacent hits of the same query were connected by genBlastA (v1.0.4; RRID:SCR_020951) [13]. Homologous blocks with a length greater than 30% of the query protein length were retained. Each connected hit region was later extended to include its 2 kb upstream and downstream flanking regions, on which gene structure was predicted by Genewise (v2.4.1; RRID:SCR_015054) [14]. MUSCLE (v3.8.31; RRID:SCR_011812) [15] was then used to align the annotated protein against its reference protein. Predicted proteins with length ≥30 amino acids and an identity value ≥40% were retained. Pseudogenes (annotated genes containing >2 frameshifts or >1 premature stop codon) and retrogenes were further removed. Next, gene models that overlapped in >40% of their coding sequence were clustered into one group, and the one with the highest identity to the reference proteins was retained to form a non-redundant gene set for each species. Two supplemental gene sets were also used for homology-based gene prediction for these newly released assemblies as above, but only the newly annotated loci from these supplemental sets were kept. Finally, all candidate annotated genes that had >10 duplications were removed. Among the protein-coding annotations of these 177 avian species, an average of 89.53% were annotated using the core avian gene set, 8.43% were supported by the transcriptome gene set, and 2.03% by the human gene set.

#### Phylogenetic information

Phylogenetic information for the 177 newly released avian species was derived from the recent large-scale avian phylogenies [16], and the tree was pruned to match the dataset using the R package ape [17], and visualized using the R package ggtree [18].

### Data validation and quality control

#### Sample taxonomic verification

Voucher specimens were verified against reference collections. Mitogenomes were BLAST searched against references in the NCBI database. If no reference mitogenome was available, we checked NCBI for commonly sequenced mitochondrial genes (*COI, ND2, CYB*) as a reference to verify sequence authenticity. For genomes that lacked a mitochondrial assembly, which was frequent when blood was the source tissue, we used a collection of nuclear markers well-represented on NCBI for birds (*FGB, GAPDH, MB, MUSK, ODC, RAG1, TGFb2*).

#### Contamination screening

Genome assemblies were screened for contamination using the NCBI Foreign Contamination Screen (FCS-GX) pipeline [19]. Adapter and contaminant sequences were detected using the FCS adaptor module, and contaminant scaffolds were removed or trimmed using the FCS cleaning module with the default parameters.

#### Quality control on sequencing reads

Quality control steps for raw reads before assembly using the SOAPfilter2 package (v2.2, RRID:SCR_000689) were as follows: remove reads with more than 10% of *N* bases; remove reads with more than 40% low-quality bases (Phred score < = 10); remove reads with undersized insert size; filter out the PCR duplicates.

#### Statistics of genome assembly

Assembly quality of 177 assemblies was assessed by contig N50, scaffold N50, and total assembly length. Genome completeness was measured with Compleasm (v0.2.7, RRID:SCR_026370) [20] using aves_odb12 as the reference gene set for these species. Three standard categories of BUSCO results were assessed as follows: complete and single-copy BUSCOs (S), complete and duplicated BUSCOs (D), fragmented BUSCOs (F). The complete and single-copy BUSCOs (S) and duplicated BUSCOs (D) hits were combined to assess the degree of genome completeness. Furthermore, to estimate the base-level accuracy and consensus quality, Merqury (v1.3, RRID:SCR_022964) [21] was utilized to calculate the Consensus Quality Value (QV) and *k*-mer completeness by comparing the assemblies against the original high-quality sequencing reads in a *k*-mer-based, reference-independent manner.

#### Genomic quality

**Figure 3:**
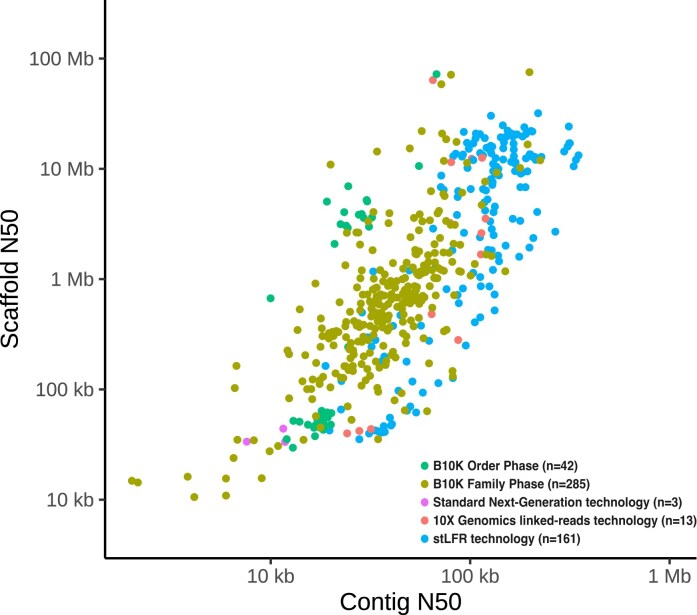
Evaluation of assembly continuity for 504 avian genomes. Scatter plot of Contig N50 versus Scaffold N50 metrics. Each dot represents a species. The dataset comprises 177 genomes from this study and 327 legacy genomes from the B10K project (Order Phase and Family Phase).

**Figure 4:**
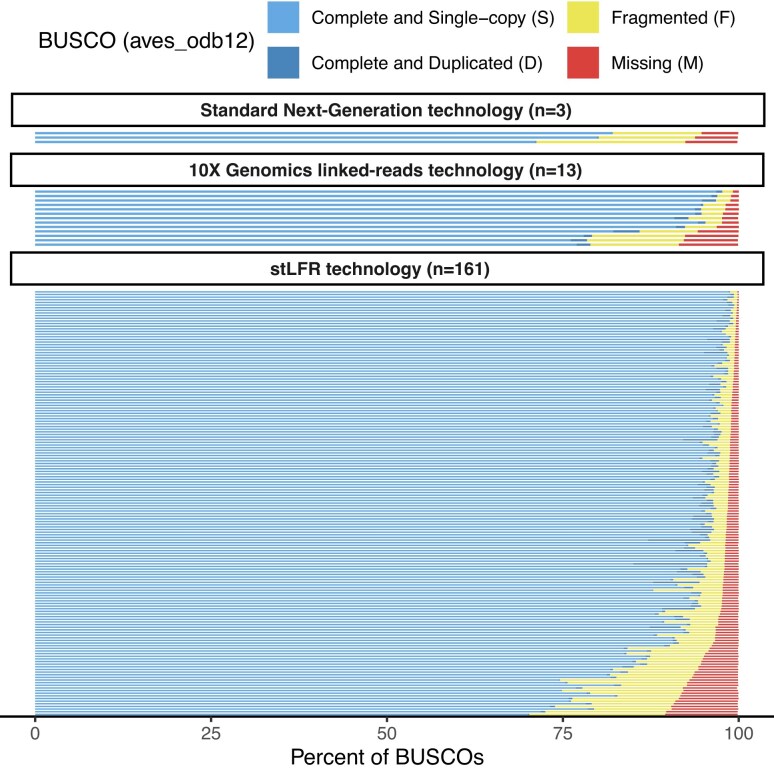
Assessment of genome assembly completeness for 177 newly sequenced avian genomes. Distribution of BUSCO scores (aves_odb12) for the 177 newly assembled genomes. Each bar represents an individual species, color-coded by gene category: complete single-copy, complete duplicated, fragmented, and missing.

The genome assemblies of the 177 avian genomes have an average genome size of ∼1.2 Gb, with average scaffold N50 = 8.03 Mb, and contig N50 = 120 kb (Fig. 3). Overall, the assembly quality shows an improvement compared with previous datasets generated at the order and family levels, largely due to the adoption of low-input sequencing strategies. Only 2.5% of the core genes in aves_odb12 could not be predicted on these newly released genomes (ranging from 0.2 to 10.2%), suggesting that the completeness of these genomes was suitable for most comparative genome analyses. An average of 93% complete core genes in these 177 avian genomes was comparable to that of the previously published genomes (Fig. 4). On average, only 4.4% of the core genes were partly annotated for the 177 avian genomes. Furthermore, *k*-mer-based evaluation yielded an average Merqury QV score of 56 (Supplementary Table 1), indicating a relatively high consensus accuracy at the base level.

**Figure 5:**
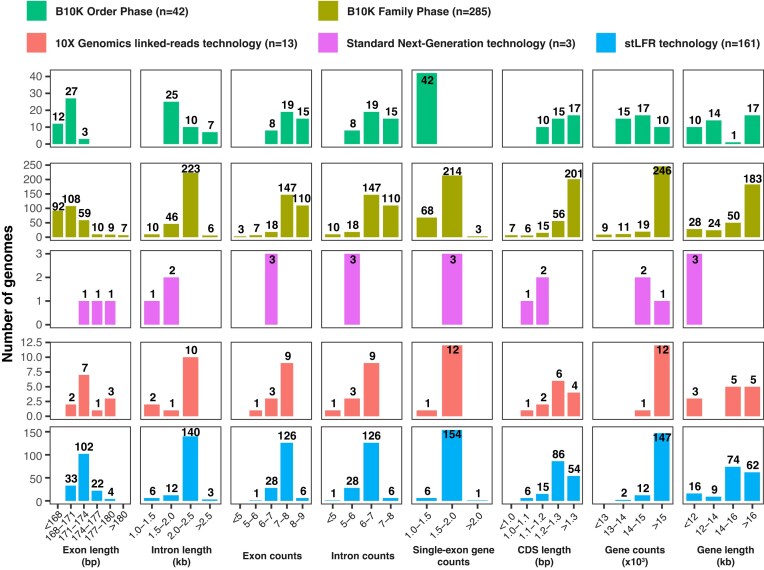
Evaluation of gene annotation quality and consistency. Distribution of eight primary annotation indicators for 504 avian genomes, stratified by B10K project phase and sequencing technology (stLFR, 10X Genomics, and NGS). From left to right, the panels display the frequency distributions of mean exon and intron lengths, mean numbers of exons and introns, single-exon gene counts, mean CDS lengths, total gene numbers, and mean gene lengths.

**Figure 6:**
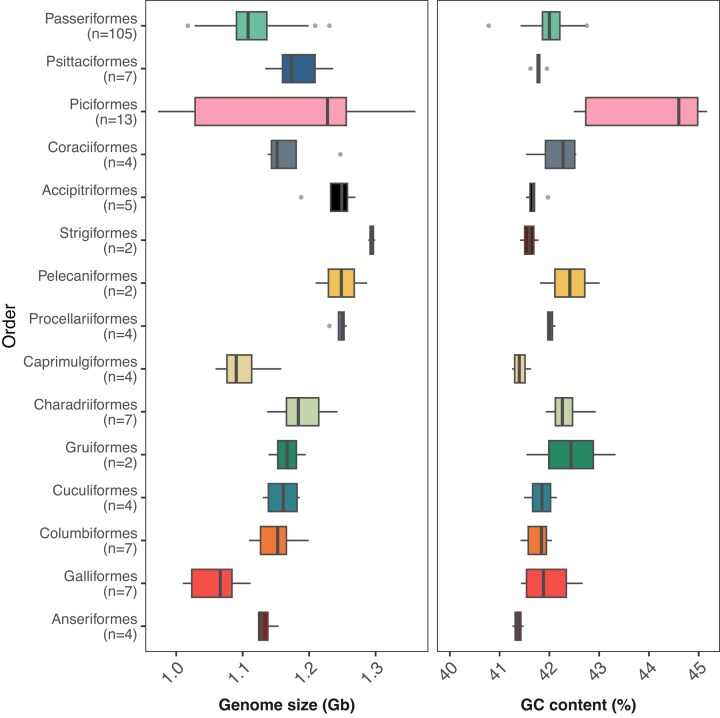
Comparative analysis of genome size and GC content across major avian taxonomic orders. Boxplots illustrating the distribution of estimated genome sizes (Gb) and GC content (%) for the 177 sequenced avian species, grouped by taxonomic order. The number of species (*n*) within each order is indicated in parentheses along the *y*-axis. For each boxplot, the thick vertical line represents the median, the box boundaries indicate the first and third quartiles (interquartile range, IQR), and the whiskers extend to the rest of the distribution, except for points that are determined to be outliers, which are plotted as individual gray dots.

Using the homologous annotation method, the 177 genomes were predicted to contain an average of 16,461 protein-coding genes, similar to previously published bird genomes [4]. The average gene length and coding sequence length are ∼15 and 1.26 kb, respectively. Genes contained on average ∼7 exons, with an average length of 172 bp and an average intron length of 2.17 kb (Supplementary Table 1). Across most annotation and assembly metrics, there was no significant difference between the two Linked-Reads technologies (10X Genomics and stLFR), both of which outperformed the standard short-read next-generation sequencing method (Fig. 5). Investigations across different avian lineages showed that Piciformes have a distinctly higher GC content with larger genome size variation (Supplementary Table 1 and Fig. 6), which is consistent with prior research [22].

### Re-use potential

We created this dataset to support future research on avian biology. While it will be useful for broad avian evolutionary studies, we expect its most significant value will be in conservation genetics and the practical management of the threatened species. Specifically, these genomic resources enable the assessments of genetic diversity in endangered birds, providing essential data to identify inbreeding depression or genetic bottlenecks. The dataset facilitates precise population genetic structure analysis and the mapping of gene flow, which are critical for designing effective translocation or captive breeding programs. Furthermore, we expect its most significant value will be in studying how global change affects the loss of genomic diversity in avian species, which is a necessary input to cope with the current challenges of the Anthropocene [23, 24].

However, since our gene prediction primarily relies on a homology-based pipeline, there may be reduced sensitivity in identifying lineage-specific genes that lack close homologs in reference databases. Additionally, potential gene fragmentation effects may exist in regions where the assembly is less contiguous, which is a common trade-off in homology-based structural annotations. It should be noted that while this study provides the protein-coding gene annotations, the identification and characterization of non-coding RNAs, such as miRNAs and lncRNAs, were not conducted in the current study. Future studies leveraging small RNA sequencing and specialized bioinformatic pipelines will be necessary to fully capture the regulatory landscape of these birds' genomes.

Despite these, the potential uses include population genomics, mapping genetic structure and gene flow, identifying hybrid zones, studying local adaptation, and investigating disease ecology. The quality of the genomes released here represents a level that was feasible at the time of this project, given the financial, technical, and biosample constraints available to the project. Nevertheless, we believe the quality will be suitable for many of the above possible end uses.

## Additional files

Supplementary Table 1.xlsx.

## Abbreviations

AviList: The Global Avian Checklist; BUSCOs: Benchmarking Universal Single-Copy Orthologs; B10K: The Bird 10,000 Genomes (B10K) Project; NCBI: National Center for Biotechnology Information; NGDC: National Genomics Data Center; stLFR: Single-Tube Long Fragment Reads.

## Supplementary Material

giag045_Supplemental_File

giag045_Authors_Response_To_Reviewer_Comments_original_submission

giag045_GIGA-D-25-00475_Original_Submission

giag045_GIGA-D-25-00475_Revision_1

giag045_Reviewer_1_Report_original_submission Reviewer 1 -- 12/21/2025

giag045_Reviewer_2_Report_original_submission Reviewer 2 -- 1/17/2026

giag045_Reviewer_3_Report_original_submission Reviewer 3 -- 2/12/2026

## Data Availability

Genome assemblies and annotations of the 177 species generated in this study have been deposited under the BioProject accession number PRJEB102986 (ENA). Sample information for each genome and the genome statistics can also be viewed online at B10K website [25]. Code to run the genome assembly pipeline can be found at [8] and the corresponding genome annotation pipeline [11]. All additional Supplementary Material is available in the GigaScience repository, GigaDB [26].

## References

[1] Dickinson E C, Remsen JV Jr (eds.). The Howard and Moore complete checklist of the birds of the world, version 4.1 (Downloadable corrigenda). Aves Press. 2018. Available at: www.howardandmoore.org.

[2] Rheindt F E, Donald P F, Donsker D B et al. AviList: a unified global bird checklist. Biodivers Conserv. 2025;34:3359–76. 10.1007/s10531-025-03120-y.

[3] Zhang G, Li C, Li Q et al. Comparative genomics reveals insights into avian genome evolution and adaptation. Science. 2014;346:1311–20. 10.1126/science.1251385. 25504712 PMC4390078

[4] Feng S, Stiller J, Deng Y et al. Dense sampling of bird diversity increases power of comparative genomics. Nature. 2020;587:252–57. 10.1038/s41586-020-2873-9. 33177665 PMC7759463

[5] Jarvis E D, Mirarab S, Aberer A J et al. Whole-genome analyses resolve early branches in the tree of life of modern birds. Science. 2014;346:1320–31. 10.1126/science.1253451. 25504713 PMC4405904

[6] Stiller J, Feng S, Chowdhury A-A et al. Complexity of avian evolution revealed by family-level genomes. Nature. 2024;629:851–60. 10.1038/s41586-024-07323-1. 38560995 PMC11111414

[7] Wang O, Cheng X, Drmanac R et al. A simple cost-effective method for whole-genome sequencing, haplotyping, and assembly. In: Peters B A, Drmanac R, eds. Methods in Molecular Biology. New York, NY: Springer; 2023. 10.1007/978-1-0716-2819-5_7. 36335495

[8] A pipeline to de novo assemble the stLFR reads using Supernova Assembler . https://github.com/BGI-Qingdao/stlfr2supernova_pipeline. Accessed 25 June 2022.

[9] Weisenfeld N I, Kumar V, Shah P et al. Direct determination of diploid genome sequences. Genome Res. 2017;27:757–67. 10.1101/gr.214874.116. 28381613 PMC5411770

[10] Luo R, Liu B, Xie Y et al. SOAPdenovo2: an empirically improved memory-efficient short-read de novo assembler. GigaScience. 2012;1. 10.1186/2047-217X-1-18. PMC3626529 23587118

[11] Scripts used in the annotation of B10K genomes . https://github.com/B10KGenomes/annotation. Accessed 25 June 2022.

[12] Altschul S F, Madden T L, Schäffer A A et al. Gapped BLAST and PSI-BLAST: a new generation of protein database search programs. Nucleic Acids Res. 1997;25:3389–402. 10.1093/nar/25.17.3389. 9254694 PMC146917

[13] She R, Chu JS-C, Wang K et al. GenBlastA: enabling BLAST to identify homologous gene sequences. Genome Res. 2009;19:143–49. 10.1101/gr.082081.108. 18838612 PMC2612959

[14] Birney E, Clamp M, Durbin R. GeneWise and Genomewise. Genome Res. 2004;14:988–95. 10.1101/gr.1865504. 15123596 PMC479130

[15] Edgar R C . MUSCLE: multiple sequence alignment with high accuracy and high throughput. Nucleic Acids Res. 2004;32:1792–97. 10.1093/nar/gkh340. 15034147 PMC390337

[16] Claramunt S, Sheard C, Brown J W et al. A new time tree of birds reveals the interplay between dispersal, geographic range size, and diversification. Curr Biol. 2025;35:3883–95. 10.1016/j.cub.2025.07.004. 40744014

[17] Paradis E, Claude J, Strimmer K. APE: analyses of phylogenetics and evolution in R language. Bioinformatics. 2004;20:289–90. 10.1093/bioinformatics/btg412. 14734327

[18] Yu G . Using ggtree to visualize data on tree-like structures. Curr Protoc Bioinformatics. 2020;69:e96. 10.1002/cpbi.96. 32162851

[19] Foreign Contamination Screening caller scripts and documentation . https://github.com/ncbi/fcs. Accessed 18 August 2026.

[20] Huang N, Li H. compleasm: a faster and more accurate reimplementation of BUSCO. Bioinformatics. 2023;39:btad595. 10.1093/bioinformatics/btad595. 37758247 PMC10558035

[21] Rhie A, Walenz B P, Koren S et al. Merqury: reference-free quality, completeness, and phasing assessment for genome assemblies. Genome Biol. 2020;21:245. 10.1186/s13059-020-02134-9. 32928274 PMC7488777

[22] Kapusta A, Suh A. Evolution of bird genomes—a transposon’s-eye view. Ann NY Acad Sci. 2017;1389:164–85. 10.1111/nyas.13295. 27997700

[23] Cassin-Sackett L, Welch A J, Venkatraman M X et al. The contribution of genomics to bird conservation. In: Kraus R, ed. Avian Genomics in Ecology and Evolution. Cham: Springer; 2019. 10.1007/978-3-030-16477-5_10.

[24] Bernatchez L, Ferchaud A-L, Berger C S et al. Genomics for monitoring and understanding species responses to global climate change. Nat Rev Genet. 2024;25:165–83. 10.1038/s41576-023-00657-y. 37863940

[25] B10K Website . https://b10k.com/. Accessed 1 April 2026.

[26] Chen G, Wang S, Øksnebjerg D B et al. Supporting data for “draft assemblies for 177 bird species enhance genus-level coverage”. GigaScience Database. 2026. 10.5524/102772. PMC13274736 42104960

[27] Bird species reference genomes in the NCBI database. https://www.ncbi.nlm.nih.gov/genome/browse/#!/aves/. Accessed 11 October 2025.

[28] The IUCN Red List of Threatened Species . https://www.iucnredlist.org. Accessed 1 April 2026.

