# Supplementary material for: Draft assemblies for 177 bird species enhance genus-level coverage
Source: Gigascience. 2026 May 9;15:giag045. doi: 10.1093/gigascience/giag045 (PMC13274736; doi:10.1093/gigascience/giag045)
Supplement: giag045_GIGA-D-25-00475_Original_Submission [file giag045_giga-d-25-00475_original_submission.pdf]

GigaScience

Draft assemblies for 177 bird species enhance genus-level coverage

--Manuscript Draft--

**Manuscript Number:**	GIGA-D-25-00475
**Full Title:**	Draft assemblies for 177 bird species enhance genus-level coverage
**Article Type:**	Data Note
**Funding Information:**	National Key Research and Development Program of China (2024YFA1802500) — Prof. Guojie Zhang<br>National Natural Science Foundation of China (32422009) — Prof. Shaohong Feng<br>National Key Research and Development Program of China (2023YFA1800500) — Prof. Shaohong Feng<br>Postdoctoral Fellowship Program of CPSF Grant (GZB2025059) — Dr. Guangji Chen<br>Danish National Research Foundation (DNRF143) — Prof. M. Thomas P. Gilbert<br>Independent Research Fund Denmark (1054-00039B) — Prof. Peter A. Hosner<br>Villum Fonden (25925) — Prof. Peter A. Hosner

Abstract:

Background

With over 10,000 recognized species, birds constitute one of the most diverse and widely distributed vertebrate groups. Although avian genomics has advanced rapidly over the past decade, substantial gaps remain across the global avifauna. Filling these gaps is essential for understanding macroevolutionary patterns, population structure, and the molecular basis of ecological and behavioral diversity. Worldwide museum collections represent invaluable resources for filling these gaps, yet the typically degraded DNA and limited quantities from historical specimens have posed significant challenges for generating high-quality genome assemblies.

Results

Here, the Bird Genome 10K (B10K) Project adopted low-input sequencing strategies that reduce costs while improving assembly quality compared with earlier order- and family-level genomes. Using mainly st-LFR, complemented by 10X Genomics and standard next-generation sequencing, we assembled 177 avian genomes from museum specimens and tissue collections representing 161 genera, including 102 newly sequenced at the genomic level. The assemblies average ~1.2 Gb in size, with scaffold N50 = 8.03 Mb, contig N50 = 120 kb, and 93% BUSCO completeness.

Conclusions

These genomes greatly expand avian taxonomic coverage and demonstrate the efficiency of low-input sequencing for generating high-quality assemblies from limited and often degraded material sourced from museum specimens. This resource provides a foundation for comparative genomics, conservation genetics, and evolutionary studies across the avian tree of life.

**Corresponding Author:**	Guojie Zhang<br>Zhejiang University<br>Hangzhou, Zhejiang CHINA
**Corresponding Author Secondary Information:**	
**Corresponding Author's Institution:**	Zhejiang University

Powered by Editorial Manager® and ProduXion Manager® from Aries Systems Corporation

**Corresponding Author's Secondary Institution:**	
**First Author:**	Guojie Zhang
**First Author Secondary Information:**	
**Order of Authors:**	Guojie Zhang
	Guangji Chen
	Shuang Wang
	Daniel Bilyeli Øksnebjerg
	Sascha Dreyer Nielsen
	Wei Dai
	Wei Jiang
	Jing Liang
	Wei Han
	Chengran Zhou
	Qiye Li
	Bent Petersen
	Ara Monadjem
	Diego Ocampo
	Luis Sandoval
	Jörns Fickel
	Alex Greenwood
	Claudia Szentiks
	Marco Roller
	Sharon Birks
	Adam D. Leaché
	Alejandro Rico-Guevera
	Jérôme Fuchs
	Nguyen Tran Vy
	Christina Hvilsom
	Juliana Andrea Berner
	Jan Terje Lifjeld
	Arild Johnsen
	Lars Erik Johannessen
	Kim Labuschagne
	Knud Andreas Jønsson
	Martin Irestedt
	Leo Joseph
	Olof Hellgren
	Robb Brumfield
	Theresa Burg

Powered by Editorial Manager® and ProduXion Manager® from Aries Systems Corporation

	Alexandre Aleixo
	Ben Smit
	Frank Rheindt
	Jessica Lee
	Isao Nishiumi
	Javier Quesada Lara
	John P. Dumbacher
	Manuel Schweizer
	Michael Andersen
	Christopher Witt
	Richard Phillips
	Richard Prum
	Kristof Zyskowski
	Steve Goodman
	Marie Jeanne Raherilalao
	Ulf Ottosson
	Yahkat Barshep
	Sam Ivande
	Vojtěch Brlík
	Erich D. Jarvis
	Carsten Rahbek
	Fumin Lei
	Gary Graves
	Shaohong Feng
	Peter A. Hosner
	M. Thomas P. Gilbert
	Zamekile D. Bhembe
	Machawe Maphalala
	Emmanuel Okposio
**Order of Authors Secondary Information:**	
**Additional Information:**	
**Question**	**Response**
Are you submitting this manuscript to a special series or article collection?	No
**Experimental design and statistics**<br><br>Full details of the experimental design and statistical methods used should be given in the Methods section, as detailed in our Minimum Standards Reporting Checklist.	Yes

Information essential to interpreting the data presented should be made available in the figure legends.<br><br>Have you included all the information requested in your manuscript?	
**Resources**<br><br>A description of all resources used, including antibodies, cell lines, animals and software tools, with enough information to allow them to be uniquely identified, should be included in the Methods section. Authors are strongly encouraged to cite Research Resource Identifiers (RRIDs) for antibodies, model organisms and tools, where possible.<br><br>Have you included the information requested as detailed in our Minimum Standards Reporting Checklist?	Yes
**Availability of data and materials**<br><br>All datasets and code on which the conclusions of the paper rely must be either included in your submission or deposited in publicly available repositories (where available and ethically appropriate), referencing such data using a unique identifier in the references and in the "Availability of Data and Materials" section of your manuscript.<br><br>Have you have met the above requirement as detailed in our Minimum Standards Reporting Checklist?	Yes
GigaScience has policies and guidelines in place for the use of generative AI-writing tools such as ChatGPT. If you have used such writing tools to assist with writing the manuscript this must be declared and cited in the text. Authors should not list AI-writing tools and other AI-assisted technologies as an author or	No

co-author and should acknowledge that they are fully responsible for text generated or refined by AI-writing tools.<p><br><br>A summary of use (particularly in the introduction or among methods) needs to be included at the end of the paper, and the outputs should also be included as a supplementary file hosted in GigaDB or other open repositories. Please read our guidelines for more information. <p><br><br>By submitting to GigaScience, you are aware of the journal's AI-writing tools policy, and if you have declared use of such tools below, you have acknowledged this where appropriate in your manuscript and have made a summary of use and outputs available. <p><br><b>AI-assisted writing tools have been used in the preparation of this manuscript?	

Powered by Editorial Manager® and ProduXion Manager® from Aries Systems Corporation

Manuscript Click here to access/download;Manuscript;Manuscript.docx

Draft assemblies for 177 bird species enhance genus-level coverage

Guangji Chen[1,*], Shuang Wang[1,*], Daniel Bilyeli Øksnebjerg[2,*], Sascha Dreyer Nielsen[2,*], Wei Dai[3,*], Wei Jiang[4], Jing Liang[1], Wei Han[1], Chengran Zhou[3], Qiye Li[3,4,5], Bent Petersen[2,6], Ara Monadjem[7,8], Zamekile D. Bhembe[7], Machawe Maphalala[9], Diego Ocampo[10], Luis Sandoval[11,12], Jörns Fickel[13,14], Alex Greenwood[15,16], Claudia Szentiks[15], Marco Roller[17,18,19], Sharon M. Birks[20], Adam D. Leaché[20], Alejandro Rico-Guevera[20,21], Jérôme Fuchs[22], Nguyen Tran Vy[23], Christina Hvilsom[24], Julianan Andrea Berner[24], Jan Terje Lifjeld[25], Arild Johnsen[25], Lars Erik Johannessen[25], Kim Labuschagne[26], Knud Andreas Jønsson[27,28], Martin Irestedt[28], Leo Joseph[29], Olof Hellgren[30], Robb Brumfield[31,32], Theresa M. Burg[33], Alexandre Aleixo[34,35], Ben Smit[36], Frank Rheindt[37], Jessica Lee[38], Isao Nishiumi[39], Javier Quesada Lara[40], John P. Dumbacher[41], Manuel Schweizer[42,43], Michael Andersen[44], Christopher C. Witt[44], Richard A. Phillips[45], Richard Prum[46], Kristof Zyskowski[47], Steve M. Goodman[48], Marie Jeanne Raherilalao[49,50], Ulf Ottosson[51], Yahkat Barshep[51,52], Sam Ivande[51,53], Vojtěch Brlík[54], Emmanuel Okposio[55], Erich D. Jarvis[56], Carsten Rahbek[57,58], Fumin Lei[59], Gary Graves[57,60], Shaohong Feng[1,61,#], Peter A. Hosner[27,57,58,#], M. Thomas P. Gilbert[2,62,#], Guojie Zhang[1,#]

Affiliations:

1. Center for Evolutionary & Organismal Biology, Liangzhu Laboratory & Women's Hospital, Zhejiang University School of Medicine, Hangzhou, China
2. Center for Evolutionary Hologenomics, Globe Institute, University of Copenhagen, Copenhagen, Denmark
3. BGI Research, Wuhan, China
4. State Key Laboratory of Genome and Multi-omics Technologies, BGI Research, Shenzhen, China
5. College of Life Sciences, University of Chinese Academy of Sciences, Beijing, China
6. Senckenberg Natural History Collections Dresden, Dresden, Germany
7. Department of Biological Sciences, University of Eswatini, Kwaluseni, Eswatini
8. Mammal Research Institute, Department of Zoology and Entomology, University of Pretoria, Hatfield, Pretoria, South Africa
9. Centre for Functional Biodiversity, School of Life Sciences, University of KwaZulu-Natal, Scottsville, P/Bag X01, Pietermaritzburg, 3209 South Africa
10. Escuela de Informática y Programa de Gestión Financiera, Comisión Institucional de Teletrabajo, Universidad Nacional, Costa Rica
11. Laboratorio de Ecología Urbana y Comunicación Animal, Escuela de Biología, Universidad de Costa Rica, San José, Costa Rica
12. Centro de Investigación en Biodiversidad y Ecología Tropical, Universidad de Costa Rica, San José, Costa Rica
13. Department of Evolutionary Genetics, IZW, Alfred-Kowalke-Strasse 17, 10315 Berlin, Germany
14. Institute of Biochemistry and Biology, Potsdam University, Karl-Liebknecht-Straße 24-25, 14476 Potsdam, Germany
15. Leibniz Institute for Zoo and Wildlife Research, Berlin, Germany

16. Department of Veterinary Medicine, Freie Universität Berlin, Berlin, Germany
17. Wilhelma Zoological-Botanical Gardens Stuttgart, Wilhelma 13, D-70376 Stuttgart, Germany
18. Department of Animal Sciences, Division of Microbiology and Animal Hygiene, Faculty of AgriculturalScience, Georg-August-University, Burckhardtweg 2, D-37077 Göttingen, Germany
19. Institute for Microbiology, University of Veterinary Medicine Hannover, Foundation, Bischofsholer Damm15, D-30173 Hannover, Germany
20. Burke Museum of Natural History and Culture, University of Washington, Seattle, Washington, USA
21. Department of Biology, University of Washington, Seattle, WA 98105, USA
22. Institut de Systématique, Evolution, Biodiversité (ISYEB), Muséum national d'Histoire naturelle, Paris, France
23. Institute of Tropical Biology, VAST, Ho Chi Minh City, Vietnam
24. Copenhagen Zoo, Copenhagen, Denmark
25. Natural History Museum, University of Oslo, Norway
26. South African National Biodiversity Institute(SANBI), Pretoria, SouthAfrica
27. Natural History Museum of Denmark, University of Copenhagen, Copenhagen, Denmark
28. Department of BioinformaΘcs and GeneΘcs, Swedish Museum of Natural History, Stockholm, Sweden
29. Australian National Wildlife Collection, National Research Collections Australia, CSIRO, Canberra, Australia
30. Department of Biology, Lund University, Lund, Sweden
31. Museum of Natural Science, Louisiana State University, Baton Rouge, LA 70803, USA.
32. Department of Biological Sciences, Louisiana State University, Baton Rouge, LA 70803, USA.
33. Department of Biological Sciences, University of Lethbridge, Lethbridge, AB, T1K 3M4, Canada
34. Department of Zoology, Museu Paraense Emílio Goeldi, Belém, PA, Brazil
35. Finnish Museum of Natural History, University of Helsinki, Helsinki, Finland
36. Department of Zoology and Entomology, Rhodes University, Makhanda, South Africa
37. Department of Biological Sciences, National University of Singapore, Singapore 117558, Singapore
38. Mandai Nature, Mandai, Singapore
39. Department of Zoology, National Museum of Nature and Science, Tokyo, Japan
40. Natural Sciences Museum of Barcelona, Barcelona, Spain
41. Ornithology & Mammalogy Department, California Academy of Sciences, San Francisco, CA 94118, United States
42. Natural History Museum Bern, Bern, Switzerland
43. Division of Population Genetics, Institute of Ecology and Evolution, University of Bern, Bern, Switzerland
44. Museum of Southwestern Biology, University of New Mexico, New Mexico, United States of America
45. British Antarctic Survey (BAS), Natural Environment Research Council (NERC), High Cross, Madingley Road, CB3 0ET Cambridge, United Kingdom
46. Department of Ecology and Evolutionary Biology, and Peabody Museum of Natural History, Yale University, New Haven, CT, USA

47. Division of Vertebrate Zoology, Peabody Museum of Natural History, Yale University, New Haven, CT USA
48. Field Museum of Natural History, Chicago, IL 60605, USA
49. Association Vahatra, Antananarivo, Madagascar
50. Mention Zoologie et Biodiversité Animale, Université d'Antananarivo, Madagascar
51. A.P. Leventis Ornithological Research Institute (APLORI), Centre of Excellence, University of Jos Biological Conservatory, Jos, Nigeria
52. Department of Zoology, University of Jos, Jos, Nigeria
53. Global Center for Species Survival, Indianapolis Zoo, 1200 West Washington St. Indianapolis, IN 46222, United States
54. Department of Ecology, Charles University, Prague, Czech Republic
55. Department of Biology, California State University, Fresno, California, USA
56. The Vertebrate Genome Laboratory, The Rockefeller University, New York, USA
57. Center for Macroecology, Evolution and Climate, Globe Institute, University of Copenhagen, Copenhagen, Denmark
58. Center for Global Mountain Biodiversity, Globe Institute, University of Copenhagen, Copenhagen, Denmark
59. Institute of Zoology, Chinese Academy of Sciences, Beijing, China
60. Department of Vertebrate Zoology, National Museum of Natural History, Smithsonian Institution, Washington DC, USA
61. Department of General Surgery of Sir Run Run Shaw Hospital, Zhejiang University School of Medicine, Hangzhou, China
62. University Museum, NTNU, Trondheim, Norway

* contributed equally
# Corresponding author

Abstract

Background: With over 10,000 recognized species, birds constitute one of the most diverse and widely distributed vertebrate groups. Although avian genomics has advanced rapidly over the past decade, substantial gaps remain across the global avifauna. Filling these gaps is essential for understanding macroevolutionary patterns, population structure, and the molecular basis of ecological and behavioral diversity. Worldwide museum collections represent invaluable resources for filling these gaps, yet the typically degraded DNA and limited quantities from historical specimens have posed significant challenges for generating high-quality genome assemblies. **Results**: Here, the Bird Genome 10K (B10K) Project adopted low-input sequencing strategies that reduce costs while improving assembly quality compared with earlier order- and family-level genomes. Using mainly st-LFR, complemented by 10X Genomics and standard next-generation sequencing, we assembled 177 avian genomes from museum specimens and tissue collections representing 161 genera, including 102 newly sequenced at the genomic level. The assemblies average ~1.2 Gb in size, with scaffold N50 = 8.03 Mb, contig N50 = 120 kb, and 93% BUSCO completeness. **Conclusions**: These genomes greatly expand avian taxonomic coverage

and demonstrate the efficiency of low-input sequencing for generating high-quality assemblies from limited and often degraded material sourced from museum specimens. This resource provides a foundation for comparative genomics, conservation genetics, and evolutionary studies across the avian tree of life.

Keywords: birds; genome sequencing; biodiversity.

Context

Birds, derived from theropod dinosaurs, constitute one of the most species-rich and widely distributed vertebrate radiations, encompassing more than 10,000 species and over 2,000 genera that inhabit nearly every ecosystem on Earth[1,2]. Over the past decade, facilitated by international efforts such as the Bird 10,000 Genomes (B10K) Project[3,4] and the Vertebrate Genomes Project, avian genome resources have expanded rapidly (**Figure 1**), providing unprecedented opportunities for comparative and functional genomics. This growth has been enabled not only by advances in sequencing and assembly technologies but also by the invaluable contribution of natural history museums and institutional biobanks, whose curated specimens have become indispensable for large-scale genome initiatives.

The B10K Project has achieved significant milestones over the past decade in generating genomic data for species representing order- and family-level diversity across the avian tree of life[3–6]. However, genus- and species-level coverage remains highly uneven. Several genera, such as *Aphelocoma*, *Falco*, *Gallus*, *Anas*, and *Haemorhous* are represented by dozens of assemblies, whereas many lineages remain underrepresented due to limited access to fresh tissues or vouchered samples. For numerous species, especially those inhabiting remote regions or represented primarily by historical specimens, natural history collections remain the only feasible sources of DNA. However, these collections are often geographically dispersed and difficult to access for genomic research due to limitations in both the quality (high-molecular-weight genomic DNA) and quantity of available genetic material. As a result, molecular-level understanding of these taxa has long been constrained by the scarcity of genomic resources.

Recent advances in low-input sequencing and museomics technologies (e.g., st-LFR) are gradually bridging this gap, allowing for the assembly of genomes from previously inaccessible samples. B10K Project has strategically adopted these low-input sequencing methods, reducing costs while improving genome assembly quality compared to earlier order- and family-level studies. Here, using st-LFR technologies, we report draft genome assemblies for 177 bird species representing 161 genera, including 102 genera newly covered at the genomic level (**Figure 2**). These assemblies substantially enrich the genomic resources available for avian research, offering new insights into the genetic diversity and evolutionary history of this remarkable vertebrate group.

Materials and Methods

Sampling, sequencing, assembly, and annotation

DNA extraction

Genomic DNA was extracted from blood and tissue samples using the 'MagMAX DNA Multi-Sample Ultra 2.0` kit (Thermo Fisher) following the manufacturer's guidelines. Post-lysis, we included an RNase A step at 37°C, followed by bead-based purification and automated isolation on the Kingfisher Duo Prime using the MMX_Ultra2_Cell_Tissue_96_Duo program. Quality control was performed on the 2200 Tapestation System, and all DNA extractions were stored at -80°C until library construction.

Library construction and sequencing

Linked-Reads Library

For the 13 samples prepared using the 10X Genomics Chromium library system, high-molecular-weight genomic DNA was processed with the Chromium Genome Library Kit (10X Genomics, Pleasanton, USA) to generate barcoded linked-read libraries. To ensure compatibility and sequencing efficiency across platforms, the libraries were adapted for sequencing on the BGISEQ-500 platform (BGI-Shenzhen, China) following in-house optimization to produce 150 bp paired-end reads in total 1.6 Tb raw sequencing reads (on average ~ 103x).

For the 161 samples prepared using st-LFR (Single-Tube Long Fragment Reads) technology (MGI), high-molecular-weight genomic DNA was processed following the manufacturer's barcoding protocol, which enables the physical linkage of short reads derived from the same long DNA fragment. The barcoded libraries were sequenced on the DNBSEQ-T7 platform (MGI, Shenzhen, China) with 100+100+42 bp paired-end reads in total 26.3 Tb raw sequencing reads (on average ~ 136x).

Standard Next-Generation Sequencing

For the 3 samples sequenced using standard short-read next-generation technology, genomic DNA was extracted from tissues or blood using either the phenol–chloroform method or a commercial genomic DNA extraction kit. DNA integrity was verified via agarose gel electrophoresis and quantified using a Qubit fluorometer. Sequencing libraries with an average insert size of approximately 350 bp were constructed according to the manufacturer's protocol and sequenced on the DNBSEQ-T1 platform (BGI-Shenzhen, China) to produce 150 bp paired-end reads in total 226 Gb raw sequencing reads (on average 63x).

Assembly and Statistics of genomes

St-LFR and 10X Genomics

For the 161 samples sequenced using st-LFR technology, data were initially transformed into 10X Genomics linked-reads format. Subsequently, this sample set, together with 13 samples sequenced by 10X Genomics linked-reads technology, were introduced into the Supernova software (v2.0.1) [7] to assemble the genomes under the "pseudohap" mode. After removing scaffolds with "N" > 80%, gap filling was applied with Gapcloser (v1.12) [8] and the paired-end information as above.

Standard Next-Generation technology

For the 3 samples sequenced by the standard Next-Generation technology, clean reads were assembled using the SOAPdenovo (v2.04) [8] with a K-mer size of 23-mer following the B10K family-phased assembly strategy [4]. After removing scaffolds with "N" > 80%, GapCloser (v1.12) [8] was used to close the intra-scaffold gaps based on the paired-end information.

Gene structures annotation

Annotation of protein-coding genes was conducted with a homology-based method for both the 177 bird species following the pipeline implemented by the Bird 10,000 Genomes (B10K) consortium[4]. The reference gene set consists of the primary reference gene set (20,194 genes), the supplemental human gene set (20,169 genes), and the supplemental transcriptome gene set (5,257 transcripts). The protein sequences in the primary reference gene set were first aligned to each genome using TBLASTN (v2.2.26) [9] with an e-value cut-off 1e-5, and multiple adjacent hits of the same query were connected by genBlastA (v1.0.4) [10]. Homologous blocks with a length greater than 30% of the query protein length were retained. Each connected hit region was later extended to include its 2 kb upstream and downstream flanking regions, on which gene structure was predicted by Genewise (v2.4.1) [11]. MUSCLE (v3.8.31) [12] was then used to align the annotated protein against its reference protein. Predicted proteins with length ≥ 30 amino acids and an identity value ≥ 40% were retained. Pseudogenes (annotated genes containing > 2 frameshifts or > 1 premature stop codon) and retrogenes were further removed. Next, gene models that overlapped in > 40% of their coding sequence were clustered into one group and the one with the highest identity to the reference proteins was retained to form a non-redundant gene set for each species. Two supplemental gene sets were also used for homology-based gene prediction for these newly released assemblies as above, but only the newly annotated loci from these supplemental sets were kept. Finally, all candidate annotated genes that had > 10 duplications were removed.

Phylogenetic information

Phylogenetic information for the 177 newly released avian species was derived from the recently large-scale avian phylogenies[13], and the tree was pruned to match the dataset using the R package ape[14], and visualized using the R packages ggtree[15].

Data Validation and quality control

Sample verification

Voucher specimens were verified against reference collections. Mitogenomes were BLAST searched against references in the NCBI database. If no reference mitogenome was available, we checked NCBI for commonly sequenced mitochondrial genes (*COI*, *ND2*, *CYB*) as a reference to verify sequence authenticity. For genomes that lacked a mitochondrial assembly, which was frequent when blood was the source tissue, we used a collection of nuclear markers well-represented on NCBI for birds (*FGB, GAPDH, MB, MUSK, ODC, RAG1, TGFb2*).

Quality control on sequencing reads

Quality control steps for raw reads before assembly using the SOAPfilter2 package (v2.2) [12] were: Remove reads with more than 10% of N bases; Remove reads with more than 40% low-quality bases (Phred score < = 10); Remove reads with undersized insert size; Filter out the PCR duplicates.

Statistics of genome assembly

Assembly quality of 177 assemblies was assessed by contig N50, scaffold N50, and total assembly length. Genome completeness was measured with Compleasm (v0.2.7)[16] using aves_odb12 as the reference gene set for these species. Three standard categories of BUSCO results were assessed as follows: Complete and single-copy BUSCOs (S), Complete and duplicated BUSCOs (D), Fragmented BUSCOs (F), The Complete and single-copy BUSCOs (S) and duplicated BUSCOs (D) hits were combined to assess the degree of genome completeness.

Genomic quality

The genome assemblies of the 177 avian genomes have an average genome size of ~ 1.2 Gb, with average scaffold N50 = 8.03 Mb, and contig N50 = 120 kb (**Figure 3**). Overall, the assembly quality shows an improvement compared with previous datasets generated at the order and family levels (**Figure 3**), largely due to the adoption of low-input sequencing strategies. Only 2.50% of the core genes in aves_odb12 could not be predicted on these newly released genomes (ranging from 0.2% to 10.3%), suggesting that the completeness of these genomes was suitable for most comparative genome analyses (**Figure 3**). An average of 93% complete core genes in these 177 avian genomes was comparable to that of the previously published genomes (**Figure 4**). On average, only 4.45% of the core genes were partly annotated for the 177 avian genomes.

Using the homologous annotation method, the 177 genomes were predicted to contain an average of 16,477 protein-coding genes, similar to previously published bird genomes[4]. The average gene length and coding sequence length are ~15 and 1.26 kb, respectively. Genes contained on average ~7 exons, with an average length of 172 bp and an average intron length of 2.17 kb (**Supplementary Table 1**). Across most annotation and assembly metrics, there was no significant difference between the two Linked-Reads technologies (10X Genomics and st-LFR), both of which outperformed the standard short-read next-generation sequencing method (**Figure 5**).

Re-use potential

We created this dataset to support future research on birds. While it will be useful for broad avian evolutionary studies, we expect its most lasting value will be in conservation genetics and management of the species included. Potential uses include population genomics, mapping genetic structure and gene flow, identifying hybrid zones, studying local adaptation, and investigating disease ecology. The quality of the genomes released here represents a level that was feasible at the time of this project, given the financial, technical, and biosample constraints

available to the project. Nevertheless, we believe the quality will be suitable for many of the above possible end uses.

Supplementary data

Supplementary Tables are available online.

Data Availability

Genome assemblies, and annotations of the 177 species generated in this study have been deposited in the NGDC under accession PRJCA049655. Sample information for each genome and the genome statistics can also be viewed online at https://b10k.com/. Code to run the genome assembly pipeline can be found at: https://github.com/BGI-Qingdao/stlfr2supernova_pipeline and the corresponding genome annotation pipeline: https://github.com/B10KGenomes/annotation.

Abbreviations

AviList: The Global Avian Checklist; NCBI: National Center for Biotechnology Information; st-LFR: Single-Tube Long Fragment Reads; B10K: The Bird 10,000 Genomes (B10K) Project; BUSCOs: Benchmarking Universal Single-Copy Orthologs; NGDC: National Genomics Data Center.

Declarations

All field sampling efforts followed appropriate local permission structures, laws, and regulations, including the Nagoya Protocol and Institutional Review Boards where they apply. All tissue samples were received and processed at Globe Institute, University of Copenhagen, which is certified to receive and process biological tissues under a permit for Animal By-Products DK-123-oth-905637 issued by The Danish Veterinary and Food Administration. CITES samples received from outside the European Union are under the CITES Scientific Exchange Exemption, registration number DK 014 issued by the Danish Environmental Protection Agency.

Competing interests

The authors declare no competing interests.

Funding

This work was supported by the National Key Research and Development Program of China (no. 2024YFA1802500) to G.Z.; the National Natural Science Foundation of China grant (no. 32422009) and the National Key Research and Development Program of China grant (no. 2023YFA1800500) to S.F.; the Postdoctoral Fellowship Program of CPSF Grant (no. GZB2025059) to G.C.; Danish National Research Foundation (DNRF143) to M.T.P.G.; Independent Research Fund Denmark 1054-00039B to P.A.H., who was also supported by a research grant (25925) from VILLUM FONDEN.

Authors' contributions

E.D.J., C.R., F.L., G.G., S.F., P.A.H., M.T.P.G. and G.Z. conceived of the study. A.M., Z.D.B., M.M, D.O., L.S., J.Fi., N.T.V., A.G., C.S., M.R., S.M.B., A.D.L., A.R., J.Fu., C.H., J.A.B., J.T.L., A.J., L.E.J., K.L., K.A.J., M.I., L.J., O.H., R.B., T.M.B., A.A., B.S., F.R., Je.L., I.N., J.Q.L., J.P.D., M.S., M.A., C.C.W., R.A.Ph., R.Pr., K.Z., S.M.G., M.J.R., U.O., Y.B., S.I., V.B., E.O., F.L., G.G. and P.A.H. provided genetic samples from fieldwork. G.C., S.W., D.B.O., S.D.N., W.D., W.J., Ji.L., W.H., C.Z., Q.L.,and B.P. performed sample acquisition, lab work, and bioinformatics. G.C., S.F., P.A.H., M.T.P.G. and G.Z. drafted the manuscript, which was edited and approved by all authors.

Acknowledgements

The authors would like to thank the various field workers, museums, universities and other genetic resource archives, permitting officials, and other support and administrative staff without whom sample collection programs could not function: Natural History Museum Denmark (NHMD), University of Copenhagen, Smithsonian National Museum of Natural History (USNM), Natural History Museum of Oslo (NHMO), Senckenberg Natural History Collections Dresden (SNSD), University of Eswatini, University of Pretoria, Universidad Nacional, Costa Rica, Universidad de Costa Rica, Leibniz Institute for Zoo and Wildlife Research (IZW), Freie Universität Berlin (FUB), Wilhelma Zoological-Botanical Gardens Stuttgart, University of Veterinary Medicine Hannover (TiHo Hannover), Burke Museum of Natural History and Culture (UWBM), University of Washington (UW), Muséum national d'histoire naturelle (MNHN), Copenhagen Zoo, South African National Biodiversity Institute (SANBI), Swedish Museum of Natural History (NRM), Australian National Wildlife Collection (CSIRO ANWC), Lund University, Louisiana State University (LSU), University of Lethbridge (ULeth), Museu Paraense Emílio Goeldi (MPEG), Finnish Museum of Natural History (LUOMUS), University of Helsinki, Rhodes University, National University of Singapore (NUS), Mandai Nature, National Museum of Nature and Science Japan (NSMT), Natural Sciences Museum of Barcelona (MZB), California Academy of Sciences (CAS), Natural History Museum Bern (NMBE), University of Bern, Museum of Southwestern Biology (MSB), University of New Mexico (UNM), British Antarctic Survey (BAS), Yale University, Field Museum of Natural History (FMNH), Université d'Antananarivo, Association Vahatra, A.P. Leventis Ornithological Research Institute (APLORI), University of Jos, Indianapolis Zoo, Charles University, Institute of Zoology, Chinese Academy of Sciences (IOZ), and NTNU University Museum. We also thank the Zhejiang Lab, the Information Technology Center of ZheJiang University and China Mobile Zhejiang Co., Ltd (Hangzhou Branch) for providing computational resources.

Reference

1. Howard, R., Moore, A., Dickinson, E. C. & Remsen, J. V. *The Howard and Moore Complete Checklist of the Birds of the World*. (Aves Press, 2013).
2. Rheindt, F. E. *et al.* AviList: a unified global bird checklist. *Biodivers. Conserv.* **34**, 3359–3376 (2025).
3. Zhang, G. *et al.* Comparative genomics reveals insights into avian genome evolution and adaptation. *Science* **346**, 1311–1320 (2014).

4. Feng, S. *et al.* Dense sampling of bird diversity increases power of comparative genomics. *Nature* **587**, 252–257 (2020).
5. Jarvis, E. D. *et al.* Whole-genome analyses resolve early branches in the tree of life of modern birds. *Science* **346**, 1320–1331 (2014).
6. Stiller, J. *et al.* Complexity of avian evolution revealed by family-level genomes. *Nature* **629**, 851–860 (2024).
7. Weisenfeld, N. I., Kumar, V., Shah, P., Church, D. M. & Jaffe, D. B. Direct determination of diploid genome sequences. *Genome Res.* **27**, 757–767 (2017).
8. Luo, R. *et al.* SOAPdenovo2: an empirically improved memory-efficient short-read de novo assembler. *Gigascience* **1**, 18 (2012).
9. Altschul, S. F. *et al.* Gapped BLAST and PSI-BLAST: a new generation of protein database search programs. *Nucleic Acids Res.* **25**, 3389–3402 (1997).
10. She, R., Chu, J. S.-C., Wang, K., Pei, J. & Chen, N. GenBlastA: enabling BLAST to identify homologous gene sequences. *Genome Res.* **19**, 143–149 (2009).
11. Birney, E., Clamp, M. & Durbin, R. GeneWise and Genomewise. *Genome Res.* **14**, 988–995 (2004).
12. Edgar, R. C. MUSCLE: multiple sequence alignment with high accuracy and high throughput. *Nucleic Acids Res.* **32**, 1792–1797 (2004).
13. Claramunt, S. *et al.* A new time tree of birds reveals the interplay between dispersal, geographic range size, and diversification. *Curr. Biol.* **35**, 3883–3895.e4 (2025).
14. Paradis, E., Claude, J. & Strimmer, K. APE: Analyses of Phylogenetics and Evolution in R language. *Bioinformatics* **20**, 289–290 (2004).
15. Yu, G. Using ggtree to Visualize Data on Tree-Like Structures. *Curr. Protoc. Bioinformatics* **69**, e96 (2020).
16. Huang, N. & Li, H. compleasm: a faster and more accurate reimplementation of BUSCO. *Bioinformatics* **39**, (2023).

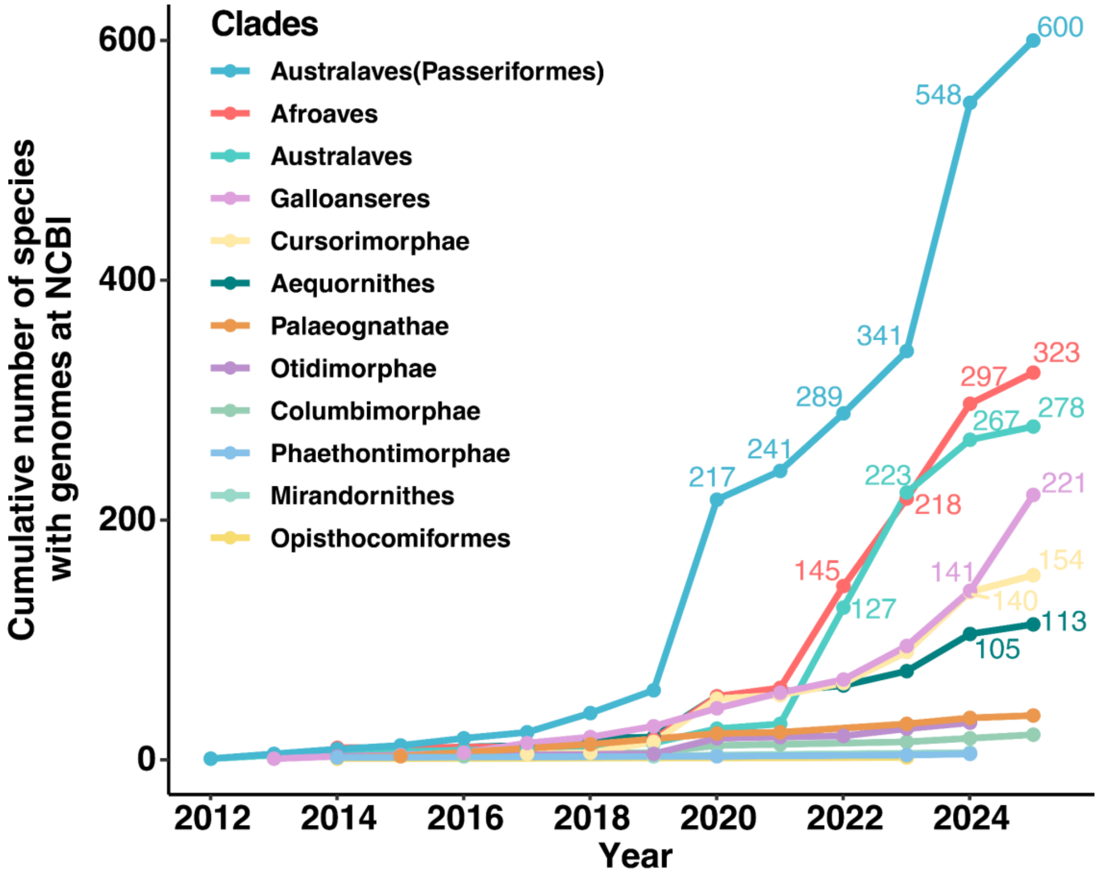

Figure 1. Cumulative number of species of the bird (coloured by clades) with reference genomes deposited at NCBI (obtained from https://www.ncbi.nlm.nih.gov/genome/browse/#!/aves/ on October 11,2025).

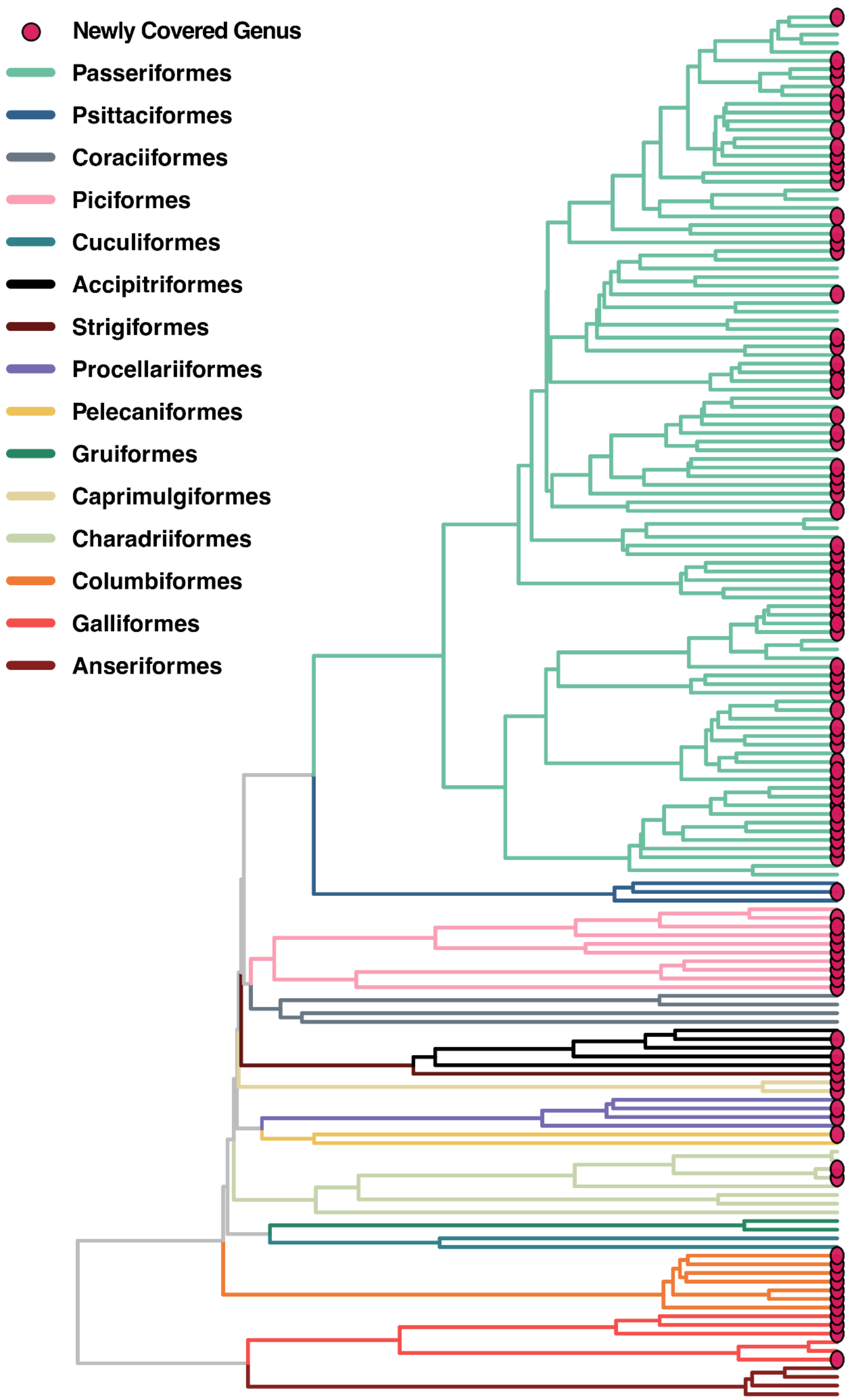

Figure 2. Phylogeny for newly released 177 avian genomes coloured by orders and highlighted the newly covered genus.

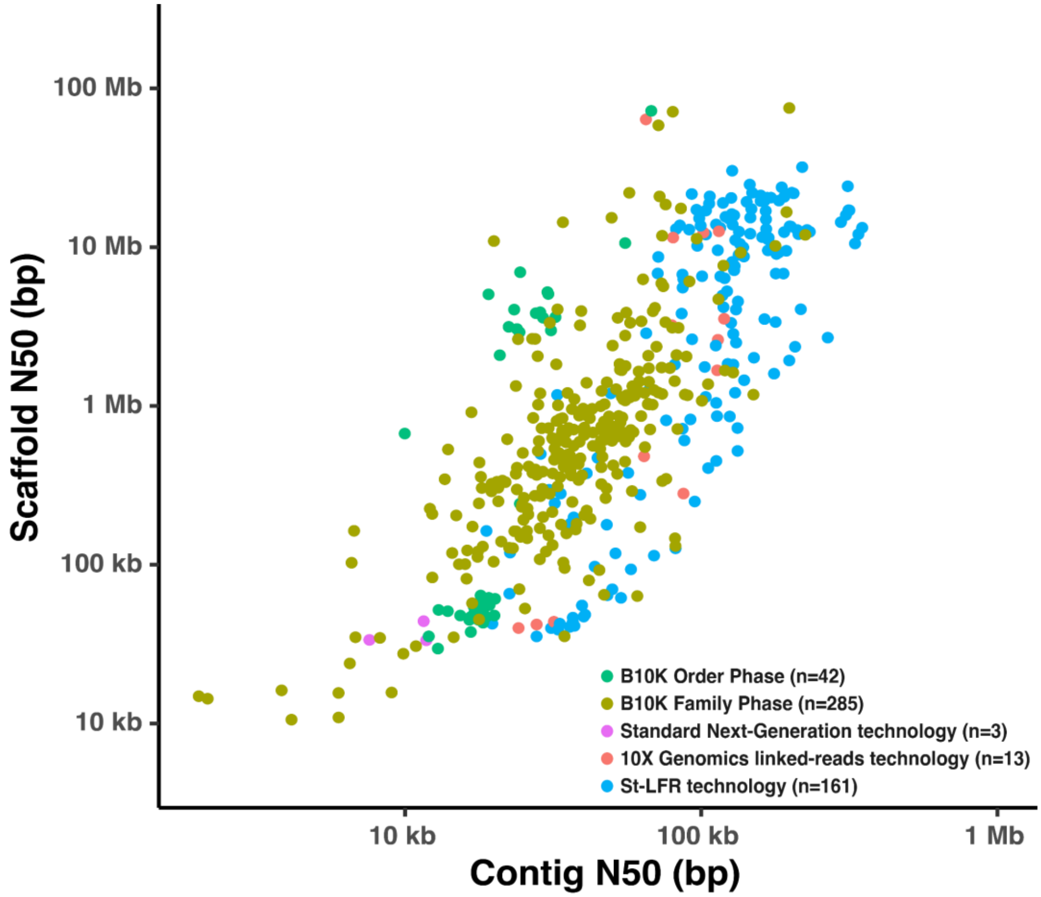

Figure 3. Assembly statistics for 177 avian genomes and 327 avian genomes of B10K Order and Family Phases.

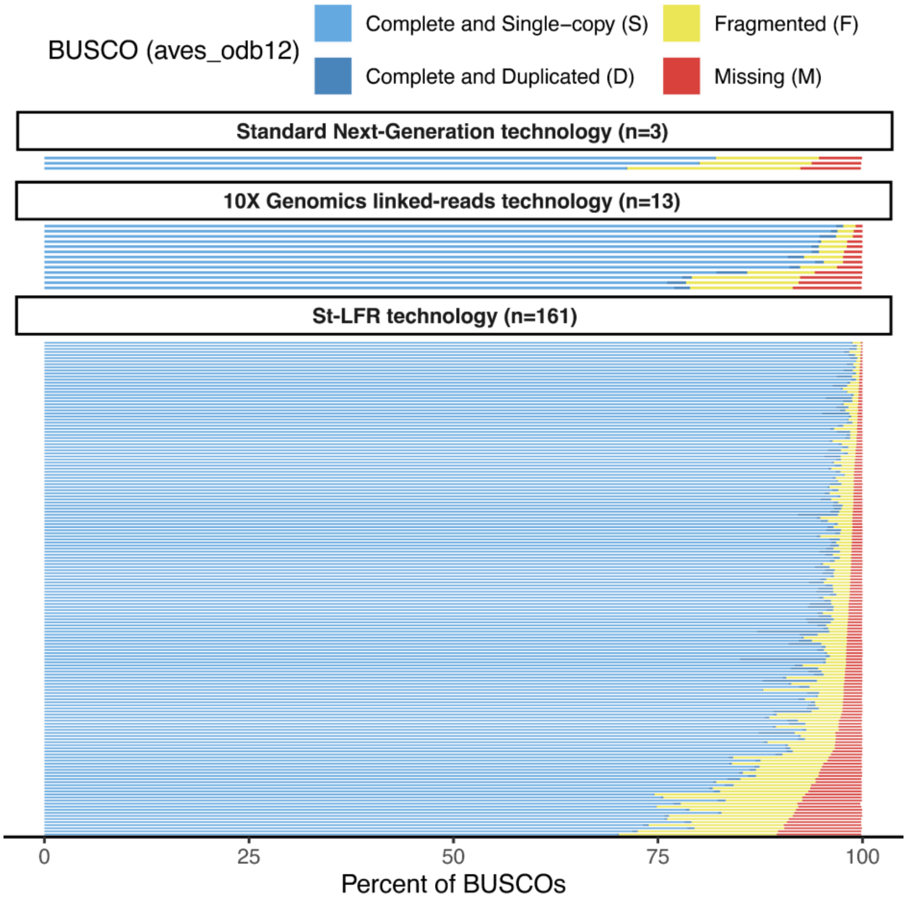

Figure 4. BUSCO evaluation for 177 newly released avian genomes.

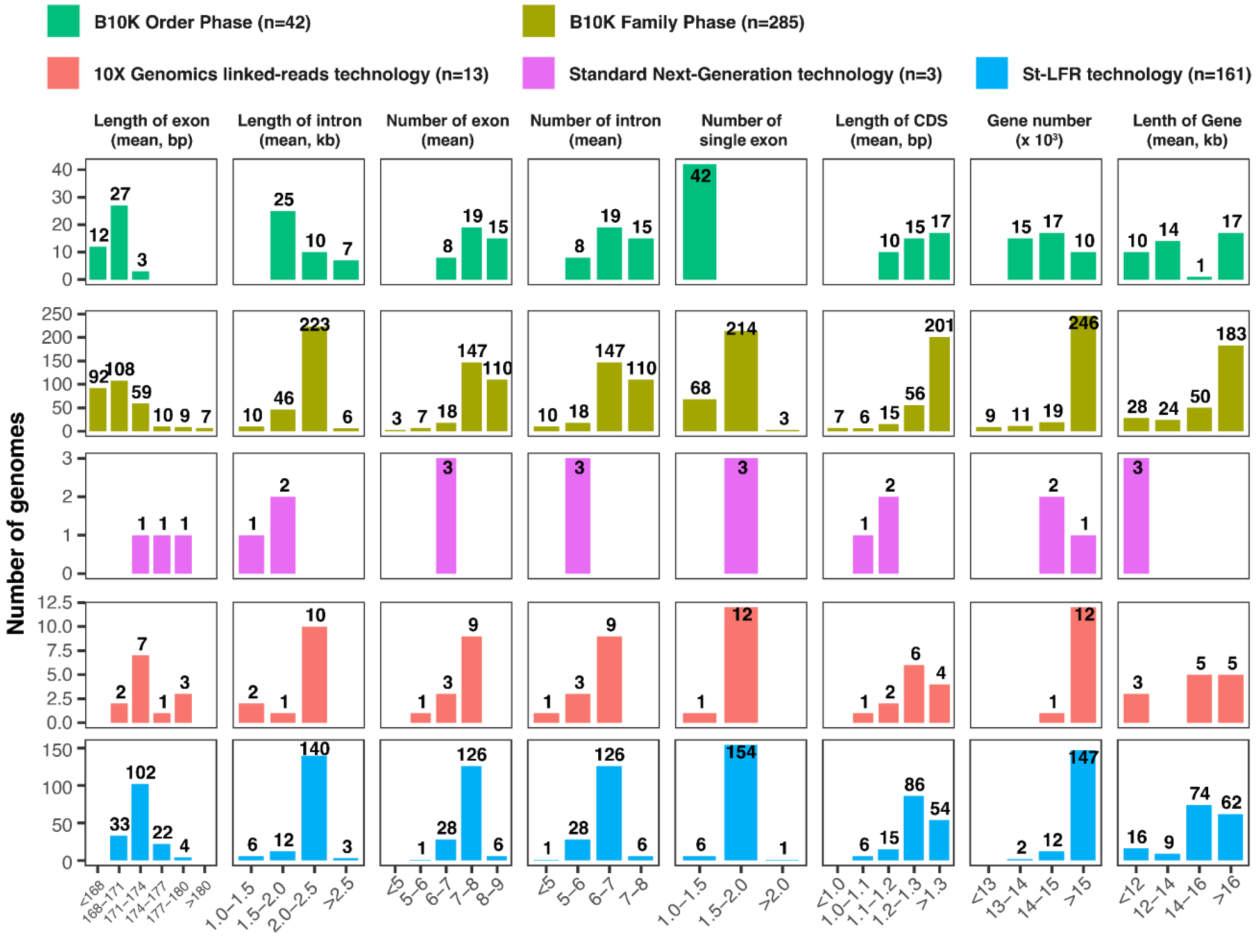

Figure 5. Eight indicators of gene annotation statistics for the 177 avian genomes and 327 avian genomes of B10K Order and Family Phases.

Figuer 1

Click here to access/download;Figure;Figure_1.p

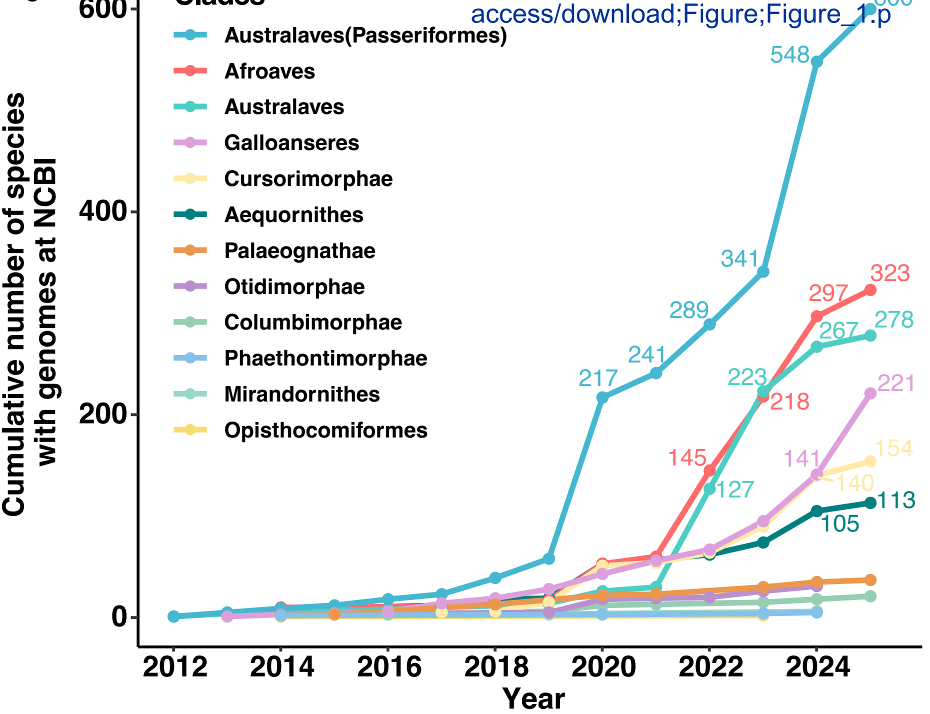

Figuer 2

Click here to access/download;

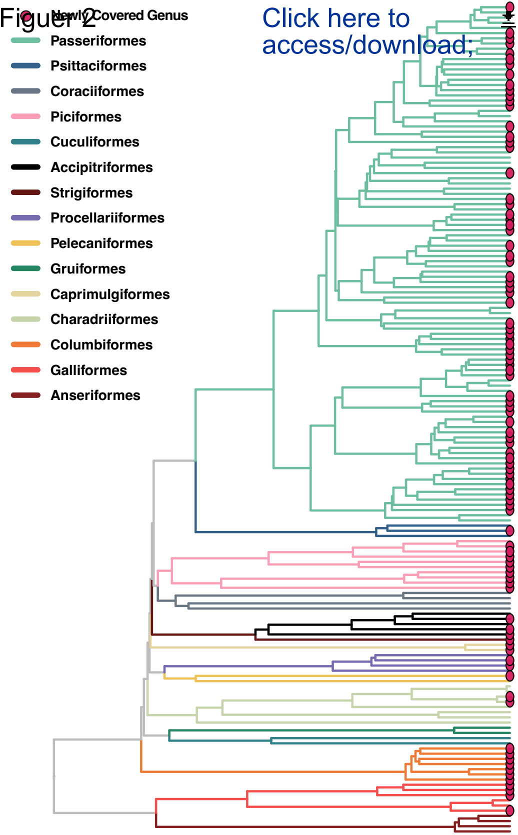

Figuer 3

Click here to access/download;Figure;Fig

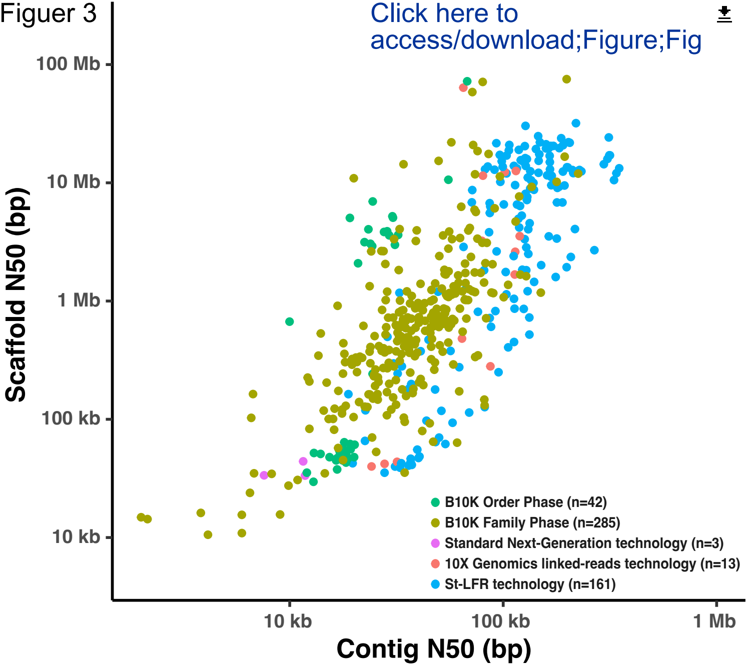

Figuer 4

Click here to access/download;Figure;Figure_4.p

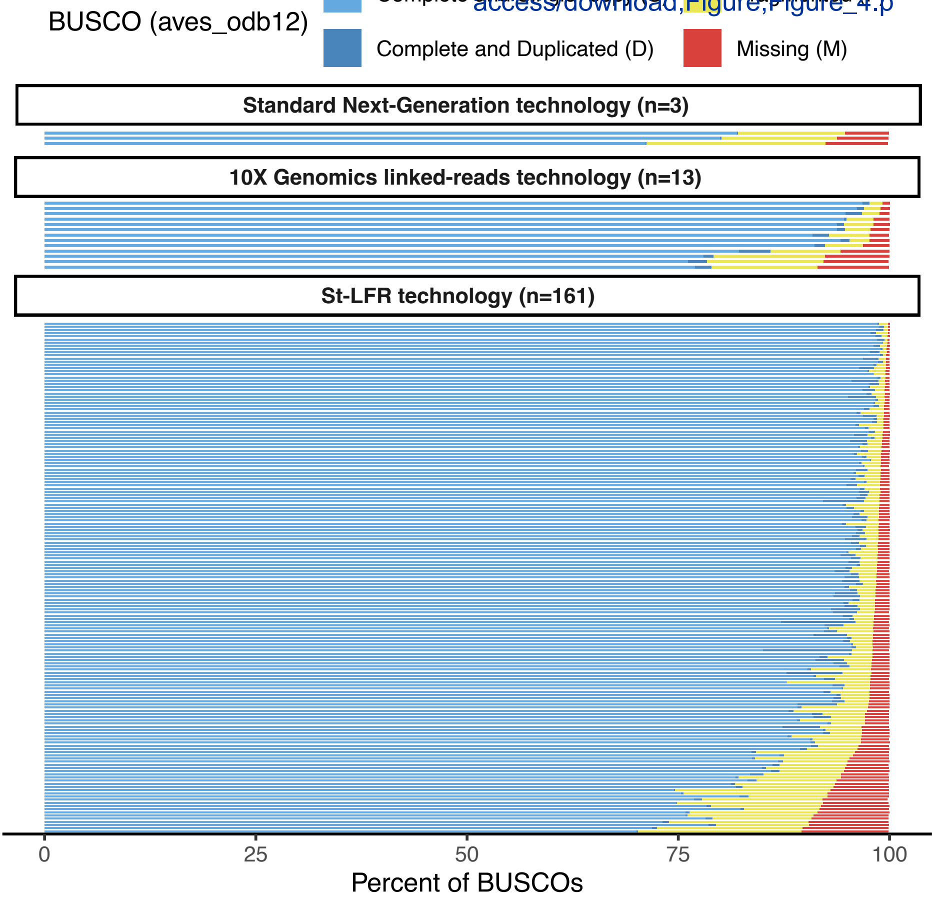

Figuer 5

Click here to access/download;Figure;Figure_5.pdf

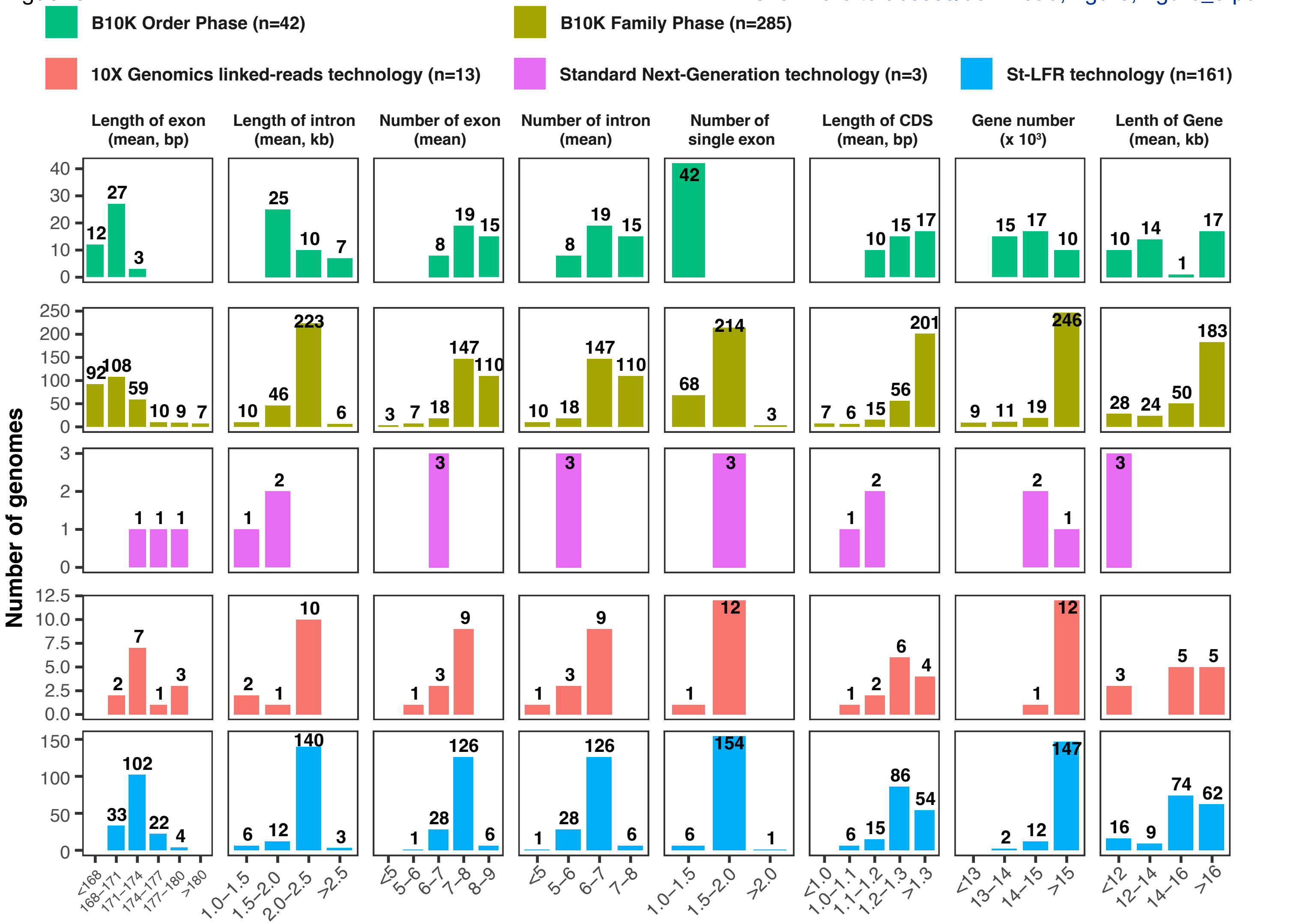

Supplementary Tables1

Click here to access/download
Supplementary Material
Supplementary Tables.xlsx

Cover letter Click here to access/download;Personal Cover;Cover Letter.pdf

ZHEJIANG UNIVERSITY
Centre for Evolutionary & Organismal Biology

School of Medicine, Zhejiang University
886# Yuhangtang Road, Xihu District
Hangzhou, Zhejiang, China
https://evolution.zju.edu.cn

Dear Editors,

We are pleased to submit our manuscript entitled "Draft assemblies for 177 bird species enhance genus-level coverage" for consideration in *GigaScience*.

Birds, derived from theropod dinosaurs, represent one of the most species-rich and widely distributed vertebrate radiations, yet genomic resources remain highly uneven across taxa. Building upon the framework of the Bird Genome 10K (B10K) Project, we report draft genome assemblies for 177 bird species representing 161 genera, including 102 genera newly covered at the genomic level.

To overcome limitations in DNA quantity and quality—particularly for samples derived from museum collections—B10K project adopted low-input sequencing strategies, using Single-Tube Long Fragment Reads (st-LFR) technology, together with 10X Genomics linked-reads technology and standard next-generation sequencing. These approaches reduced sequencing costs while improving assembly continuity relative to previous order and family phases. The resulting assemblies average ~1.2 Gb in genome size with 93% BUSCO completeness, providing a marked improvement in coverage and data quality.

This dataset substantially expands the phylogenetic and ecological representation of avian genomes and provides a valuable foundation for future research in evolutionary genomics, conservation genetics, and adaptive evolution. The complete assemblies and metadata have been publicly deposited in the National Genomics Data Center (PRJCA049655) in accordance with *GigaScience*'s commitment to open data and reproducible research.

All authors have approved the submission, declared no competing interests, and confirmed that the manuscript is not under consideration elsewhere.

Thank you very much for considering our submission. We look forward to your feedback.

Sincerely,

Guojie Zhang on behalf of all co-authors
Center of Evolutionary & Organismal Biology
Zhejiang University School of Medicine,
Hangzhou, 310058, China
E-mail: guojiezhang@zju.edu.cn
