# Supplementary material for: Draft assemblies for 177 bird species enhance genus-level coverage
Source: Gigascience. 2026 May 9;15:giag045. doi: 10.1093/gigascience/giag045 (PMC13274736; doi:10.1093/gigascience/giag045)
Supplement: giag045_GIGA-D-25-00475_Revision_1 [file giag045_giga-d-25-00475_revision_1.pdf]

GigaScience

Draft assemblies for 177 bird species enhance genus-level coverage

--Manuscript Draft--

**Manuscript Number:**	GIGA-D-25-00475R1	
**Full Title:**	Draft assemblies for 177 bird species enhance genus-level coverage	
**Article Type:**	Data Note	
**Funding Information:**	National Key Research and Development Program of China (2024YFA1802500)	Prof. Guojie Zhang
	National Natural Science Foundation of China (32422009)	Prof. Shaohong Feng
	National Key Research and Development Program of China (2023YFA1800500)	Prof. Shaohong Feng
	Postdoctoral Fellowship Program of CPSF Grant (GZB2025059)	Dr. Guangji Chen
	Danish National Research Foundation (DNRF143)	Prof. M. Thomas P. Gilbert
	Independent Research Fund Denmark (1054-00039B)	Prof. Peter A. Hosner
	Villum Fonden (25925)	Prof. Peter A. Hosner
	Spanish Ministry of Science, Innovation and Universities (PGC2018-097575-B-I00)	Prof. Juan Carlos Illera
	European Regional Development Fund (PID2022-140091NB-I00)	Prof. Juan Carlos Illera
	Villum Foundation (Young Investigator Programme) (15560)	Prof. Knud Andreas Jønsson
	Carlsberg Foundation (CF15-0079)	Prof. Knud Andreas Jønsson

Abstract:

Background

With over 10,000 recognized species, birds constitute one of the most diverse and widely distributed vertebrate groups. Although avian genomics has advanced rapidly over the past decade, substantial gaps remain across the global avifauna. Filling these gaps is essential for understanding macroevolutionary patterns, population structure, and the molecular basis of ecological and behavioral diversity. Worldwide museum collections represent invaluable resources for filling these gaps, yet the typically degraded DNA and limited quantities from historical specimens have posed significant challenges for generating high-quality genome assemblies.

Results

Here, the Bird Genome 10K (B10K) Project adopted low-input sequencing strategies that reduce costs while improving assembly quality compared with earlier order- and family-level genomes. Using mainly st-LFR, complemented by 10X Genomics and standard next-generation sequencing, we assembled 177 avian genomes from museum specimens and tissue collections representing 161 genera, including 102 newly sequenced at the genomic level. The assemblies average ~1.2 Gb in size, with scaffold N50 = 8.03 Mb, contig N50 = 120 kb, 93% BUSCO completeness, and Merqury QV score of 56.

Conclusions

These genomes greatly expand avian taxonomic coverage and demonstrate the efficiency of low-input sequencing for generating high-quality assemblies from limited and often degraded material sourced from museum specimens. This resource provides

Powered by Editorial Manager® and ProduXion Manager® from Aries Systems Corporation

	a foundation for comparative genomics, conservation genetics, and evolutionary studies across the avian tree of life.
**Corresponding Author:**	Guojie Zhang<br>Zhejiang University<br>Hangzhou, Zhejiang CHINA
**Corresponding Author Secondary Information:**	
**Corresponding Author's Institution:**	Zhejiang University
**Corresponding Author's Secondary Institution:**	
**First Author:**	Guangji Chen
**First Author Secondary Information:**	
**Order of Authors:**	Guangji Chen
	Shuang Wang
	Daniel Bilyeli Øksnebjerg
	Sascha Dreyer Nielsen
	Wei Dai
	Wei Jiang
	Jing Liang
	Wei Han
	Chengran Zhou
	Qiye Li
	Bent Petersen
	Ara Monadjem
	Diego Ocampo
	Luis Sandoval
	Jörns Fickel
	Alex Greenwood
	Claudia Szentiks
	Marco Roller
	Sharon Birks
	Adam D. Leaché
	Alejandro Rico-Guevera
	Jérôme Fuchs
	Nguyen Tran Vy
	Christina Hvilsom
	Juliana Andrea Berner
	Jan Terje Lifjeld
	Arild Johnsen
	Lars Erik Johannessen
	Kim Labuschagne

Powered by Editorial Manager® and ProduXion Manager® from Aries Systems Corporation

	Knud Andreas Jønsson
	Martin Irestedt
	Leo Joseph
	Olof Hellgren
	Robb Brumfield
	Theresa Burg
	Alexandre Aleixo
	Ben Smit
	Frank Rheindt
	Jessica Lee
	Isao Nishiumi
	Javier Quesada Lara
	John P. Dumbacher
	Manuel Schweizer
	Michael Andersen
	Christopher Witt
	Richard Phillips
	Richard Prum
	Kristof Zyskowski
	Steve Goodman
	Marie Jeanne Raherilalao
	Ulf Ottosson
	Yahkat Barshep
	Sam Ivande
	Vojtěch Brlík
	Erich D. Jarvis
	Carsten Rahbek
	Fumin Lei
	Gary Graves
	Shaohong Feng
	Peter A. Hosner
	M. Thomas P. Gilbert
	Machawe Maphalala
	Zamekile D. Bhembe
	Emmanuel Okposio
	Andrew Hart Reeve
	Juan Carlos Illera
	Bonny Koane
	Tri Haryoko
	Guojie Zhang

Powered by Editorial Manager® and ProduXion Manager® from Aries Systems Corporation

Order of Authors Secondary Information:	
Response to Reviewers:	Response to Reviewers<br>Response: We would like to thank the editor and reviewers for taking the time to review our manuscript and providing constructive suggestions to improve this work. Below, we provide a point-by-point response to reviewers' comments.<br><br>Reviewer #1:<br>As a Data Note, this manuscript centers on the draft genome assemblies of 177 bird species, substantially augmenting the genus-level taxonomic coverage of avian genomes. Significantly, high-quality genomes were successfully acquired from museum specimens and tissue samples through st-LFR, addressing many gaps in avian genomic resources at the genus level. This study offers a critical foundational dataset for comparative genomics, conservation genetics, and evolutionary research. The research design is well-defined, the technical approach is logical, and the data volume is ample with high completeness and provided a valuable dataset for scientific field.<br>Response: We appreciate your time and effort on our manuscript. Your insightful comments are very useful for us to refine our manuscript. We have carefully read your comments and addressed them in this revision.<br><br>Several comments:<br>(1) The rationale for selecting three sequencing technologies (161 samples with st-LFR, 13 samples with 10X Genomics, and 3 samples with standard next-generation sequencing) is unclear (e.g., differences in sample quality, species-specific requirements, etc.). It is recommended to supplement the initial DNA quality parameters of different samples (such as DNA integrity values, degradation degrees), explain why some samples adopted 10X Genomics or standard next-generation sequencing, and whether the impact of this differentiated strategy on assembly quality has been evaluated. This details would help this field to have a general idea about the different sequencing methods adapted to different sample quality.<br>Response: We thank the reviewer for this suggestion. The differentiated sequencing strategy was indeed an adaptive approach based on both the temporal availability of technologies and the variations in DNA integrity across different samples. The 13 samples were sequenced during the early stages of the project when 10X Genomics was the leading technology for high-quality, long-range genome assembly. It was applied to samples with exceptional DNA integrity (fragment size >40kb). As stLFR technology matured and became the primary platform, it was adopted for the 161 samples due to its lower DNA input requirements (10ng with fragment size >10kb) while still providing high-quality linked-read information. And considering the preciousness of the samples, the remaining 3 samples were relatively degraded (fragment size <10kb) and did not meet the requirements for linked-read technologies, so we opted for standard next-generation sequencing to ensure at least basic genomic coverage. And we have added these information in the "Methods" section as following: "The selection of sequencing technologies was strategically determined by the interplay between DNA integrity and the progressive availability of sequencing platforms throughout the project. The majority of the DNA samples (n = 161) were prepared using the stLFR (Single-Tube Long Fragment Reads) technology, which served as our primary pipeline due to its ability to generate high-quality linked-read data with 10 ng of input DNA with fragment sizes exceeding 10 kb. During the early phase of this study, 13 DNA samples which exhibited exceptional DNA integrity (main fragment sizes > 40 kb) were prepared using the 10X Genomics Chromium library system. For the remaining 3 samples with relative DNA degradation (fragment sizes < 10 kb) that failed to meet the requirements for linked-read library construction, standard short-read next-generation technology with approximately 500 ng input DNA was employed to ensure sufficient genomic coverage despite suboptimal sample quality."<br><br>(2) The reference gene set in homologous annotation includes core avian genes, human genes, and transcriptome data, but the rationale for using the human gene set for avian gene annotation is not explained (e.g., the risk of missing species-specific genes). Current several avian genomes and annotations can meet the T2T genome assemblies and annotations. It is recommended to supplement the screening criteria of the reference gene set and the contribution ratio of different reference sets to the annotation results (such as the proportion of genes annotated by core avian genes); meanwhile, the annotation of non-coding RNAs is not mentioned. If no relevant

analysis was conducted, it should be stated in the limitations; if it has been carried out, the relevant results need to be supplemented.
Response: We thank the reviewer for this comment regarding the reference gene sets. We agree that reliance on human data for avian annotation requires careful justification to avoid missing specific genes. To address the concern of potential bias, we have quantified the contribution of each reference set to the final gene models. Among the protein-coding annotations of these 177 avian species, on average, 89.53% by core avian genes set, 8.43% of genes supported by the transcriptome gene set, and 2.03% by human genes set. We have added the proportion of genes annotated by core avian genes into the Supplementary Table 1, and revised the related sentences in the "Gene structures annotation" and "Re-use potential" section.

We also acknowledge that the annotation of non-coding RNAs (ncRNAs) is a significant component of genome characterization. As suggested, we have clearly stated this in the "Re-use potential" section as following:
“Since our gene prediction primarily relies on a homology-based pipeline, there may be reduced sensitivity in identifying lineage-specific genes that lack close homologs in reference databases. Additionally, potential gene fragmentation effects may exist in regions where the assembly is less contiguous, which is a common trade-off in homology-based structural annotations. It should be noted that while this study provides the protein-coding gene annotations, the identification and characterization of non-coding RNAs (ncRNAs), such as miRNAs and lncRNAs, were not conducted in the current study. Future studies leveraging small RNA sequencing and specialized bioinformatic pipelines will be necessary to fully capture the regulatory landscape of these birds' genomes.”

(3) Only the average assembly and annotation indicators of the 177 genomes are presented, without grouping comparison by avian order or family-level taxonomic units, making it impossible to reflect the differences in genomic characteristics (such as genome size, gene number, intron length) among different avian groups. It is recommended to supplement grouped statistical analysis by taxonomic units to reveal the group-specific patterns of avian genomes.
Response: We thank the reviewer for this suggestion. In the revised manuscript, we have included a grouped statistical analysis of genome size and GC content across different taxonomic orders. Notably, our results show that bird species within the order Piciformes have a relatively higher average GC content of approximately 44% with larger genome size variation (Figure 6), which is consistent with previous avian genomic studies (Kapusta and Suh 2017).

(4) As a Data Note, in-depth functional analysis is not required, but combined with the conservation status of some species, the specific application scenarios of their genomic data in conservation genetics (such as population genetic structure analysis, genetic diversity assessment of endangered species) can be illustrated to enhance the application orientation of the data.
Response: We appreciate this suggestion. We agree that emphasizing the practical application of genomic data in conservation genetics will significantly enhance the value of this Data Note. As suggested, we have revised the "Re-use potential" section as following:
“While it will be useful for broad avian evolutionary studies, we expect its most significant value will be in conservation genetics and the practical management of the threatened species included. Specifically, these genomic resources enable the assessments of genetic diversity in endangered birds, providing essential data to identify inbreeding depression or genetic bottlenecks. Furthermore, the dataset facilitates precise population genetic structure analysis and the mapping of gene flow, which are critical for designing effective translocation or captive breeding programs.”

(5) It is recommended to mark the endangered species or endemic species can be marked in the figure to highlight the conservation value of the data if possible. The axis labels and legend information of Figures 3 and 5 are incomplete (e.g., some axes do not specify units, and abbreviations of indicator names are not annotated). It is recommended to standardize the figure annotations to ensure that readers can directly interpret the data.
Response: We have implemented the following improvements to enhance the clarity and impact of our data visualization. In Figure 2, we have explicitly marked IUCN

Powered by Editorial Manager® and ProduXion Manager® from Aries Systems Corporation

Status and population trend according to the IUCN Red List. We also have thoroughly revised the axis labels, legends, and captions for Figures 3 and 5.

(6) The supplementary tables do not include detailed collection information of each sample (such as collection time, location, specimen number, preservation method) and specific parameters of DNA extraction (such as RNase A treatment time, bead purification conditions). This information is crucial for other researchers to repeat the experiment or verify the data. It is recommended to improve the supplementary tables and supplement the above key experimental details.
Response: We agree that detailed sample metadata and experimental parameters are essential for data reproducibility. We have addressed these concerns as suggested. We have updated the Supplementary Table 1 to include the collection details for each of the 177 species, including collection time, location, specimen/accession numbers, and sample types. We have revised the "DNA extraction" section to provide specific technical details as
“For post-lysis, RNA degradation was performed by incubating the lysate with 10 µL RNase A at 37°C for 5 minutes. Subsequently, genomic DNA was isolated using a bead-based purification method, utilizing 440 µL of DNA Binding Bead Mix per sample. The automated isolation process was executed on the Kingfisher Duo Prime system (Thermo Fisher Scientific), following the standard MMX_Ultra2_Cell_Tissue_96_Duo protocol.”

This manuscript successfully assembled the genomes of 177 bird species using low-input sequencing technologies, significantly enhancing the genus-level coverage of avian genomes. The data quality is reliable and the sharing is good, which meets the requirements for publishing Data Notes in GigaScience. It is recommended that the authors supplement the sample selection logic, technical details, and data completeness in response to the above issues, optimize the figure and code explanations. The manuscript can be considered for acceptance after revision.
Response: We appreciate your time and effort on our manuscript. We have carefully addressed your comments in this revision.

Reviewer #2:
Overall evaluation
This Data Note reports 177 draft avian genome assemblies spanning 161 genera, including 102 genera with genomes sequenced for the first time, and provides baseline quality metrics. The dataset substantially improves genus-level genome coverage in birds and represents a valuable community resource, particularly for comparative genomics, phylogenomics, and biodiversity/conservation applications.
The work is timely and impactful. The manuscript is generally clear and well-structured, and the resource value is high. However, as a Data Note, the strongest requirement is reusability and reproducibility. I recommend a minor revision to strengthen transparency of sample provenance, reproducibility of the assembly workflow, and contamination screening/validation.
Response: We appreciate the reviewer's interest in our work. We have revised the manuscript accordingly according to the constructive and insightful comments of the reviewer.

Major comments
1) Clarify sample provenance (museum vs fresh tissue/blood) and dataset composition
The manuscript emphasizes the utility of low-input strategies and mentions museum specimens, but it is not fully clear what proportion of the 177 genomes were derived from challenging sources (e.g., historic toepads) versus fresh tissues or blood. For re-use and interpretation, the manuscript would benefit from a concise summary of sample types.
Requested improvement :
Add a short table or summary paragraph specifying, for all species:
sample source type (museum toepad/historical vs fresh/frozen tissue vs blood)
preservation condition (if known)
library type per sample (st-LFR / 10X / short-read NGS)
Response: We agree that clarifying the provenance and type of samples is essential for interpreting the assembly quality and the utility of our low-input strategies. As requested, we have provided a comprehensive breakdown of the sample metadata. We have updated the Supplementary Table 1 to include specific columns for sample

Powered by Editorial Manager® and ProduXion Manager® from Aries Systems Corporation

	source type (e.g., skin tissue, muscle tissue, or blood sample), and the corresponding library type (stLFR, 10X, or NGS) for each of the 177 species. We also noted that 80.8% of the samples (143 out of 177) were collected prior to 2020 in the revised manuscript.<br><br>2) Assembly workflow needs more parameter detail for reproducibility<br>The assembly workflow is described at a high level (st-LFR conversion to 10X format; Supernova pseudohap; Gapcloser), but critical details are missing for others to reproduce the pipeline.<br>Requested improvement:<br>Please add the main tools/commands and key parameters, including:<br>name/version of the tool for st-LFR → 10X conversion, and the essential settings<br>any major Supernova settings beyond pseudohap mode<br>Gapcloser parameters<br>clarify at what stage and how many scaffolds were removed with the "N > 80%" filtering criterion<br>Response: We thank the reviewer for emphasizing the importance of reproducibility in assembly workflow. In the revised manuscript, we have expanded the sentences to include comprehensive technical details and specific parameters. We have explicitly stated the use of the stlfr2supernova pipeline (https://github.com/BGI-Qingdao/stlfr2supernova_pipeline) for format conversion and added the command line for Supernova (v2.0.1). We also clarified the scaffold filtering process based on "N" content, which resulted in the removal of an average of 2 scaffolds per assembly. Furthermore, we revised the sentences related to GapCloser (v1.12) using the default parameters and the cleaned paired-end reads for gap filling.<br><br>3) Add contamination screening / taxonomic validation summary<br>Species validation is performed using BLAST of mitochondrial genomes (and nuclear markers when mitochondria are unavailable). This is useful, but for large datasets—especially those potentially involving museum/historic material—contamination screening is important.<br>Requested improvement :<br>Provide a short contamination screening result summary using a standard approach (e.g., Kraken2/Centrifuge summary, BlobToolKit, or similar), even if only for a subset, OR<br>Include a statement of what contamination checks were done and thresholds used<br>Response: We agree that contamination screening and taxonomic validation are critical for large-scale genomic datasets. We have now included a detailed summary of our quality control pipeline in the revised manuscript under the "Contamination screening" and "Sample taxonomic verification" sections. Specifically, we employed the NCBI Foreign Contamination Screen (FCS-GX) pipeline to systematically detect and remove adapter sequences and non-avian contaminant scaffolds (e.g., bacteria, fungi, or human DNA) using default sensitivity thresholds. Additionally, we implemented a taxonomic verification strategy using mitogenome BLAST searches against the NCBI reference database, supplemented by specific mitochondrial markers (COI, ND2, CYB) or a suite of conserved avian nuclear markers (e.g., RAG1, GAPDH, FGB) to ensure both assembly purity and the accuracy of species identification.<br><br>4) Include at least one additional assembly integrity metric (optional but strongly recommended)<br>N50 and BUSCO are informative, but many users will expect at least one additional metric that reflects base-level accuracy or k-mer completeness.<br>Requested improvement:<br>If raw reads are available, please consider reporting Merqury QV/k-mer completeness (even for a representative subset). If this is not feasible, a short justification would be acceptable.<br>Response: We thank the reviewer for this suggestion, which has further strengthened the quality assessment of our dataset. As requested, we have conducted a k-mer-based evaluation using Merqury for our assemblies, which resulted in an average Merqury QV score of ~56. We have also incorporated these results into the revised manuscript and added the specific details for individual species to the Supplementary Table 1.<br><br>5) Gene annotation section: clarify limitations of purely homology-based prediction

Powered by Editorial Manager® and ProduXion Manager® from Aries Systems Corporation

Annotation is performed using a homology-based pipeline (TBLASTN → genBlastA → GeneWise). This is reasonable for a Data Note, but users should be warned about limitations such as reduced sensitivity for lineage-specific genes and potential fragmentation effects.
Requested improvement:
Add 2-3 sentences in the "Re-use potential" section clarifying expected limitations (e.g., no RNA-seq support, structural annotations mainly, assembly fragmentation effects).
Response: We have revised the "Re-use potential" section to explicitly clarify the limitations of our homology-based annotation pipeline. As suggested, we added sentences discussing the potential reduced sensitivity for lineage-specific genes and possible gene fragmentation effects associated with structural annotations. We believe these additions provide a more transparent and realistic guide for future users of our dataset.

Minor comments
1. Please correct minor typos/formatting inconsistencies (e.g., figure label spelling and consistent "st-LFR" naming).
Response: We have revised the figure labels and consistent the naming as "stLFR" as Reviewer 3 mentioned.

2. Expand read preprocessing description slightly (adapter trimming, barcode handling, trimming thresholds) to improve clarity.
Response: We appreciate the reviewer's request for clarity on read preprocessing. While the "Quality Control" section provided a general overview, we have now explicitly integrated the technical parameters and filtering thresholds into the "Assembly" section to ensure full transparency and reproducibility as following:
"For the 161 samples sequenced using stLFR technology, raw data were initially transformed into 10X Genomics linked-reads format following the stlfr2supernova pipeline (https://github.com/BGI-Qingdao/stlfr2supernova_pipeline). Subsequently, these 161 samples, together with 13 samples sequenced by 10X Genomics linked-reads technology, were preprocessed with SOAPfilter (v2.2.1) using parameters `-i 500 -p -z -g 1 -M 2 -Q 10 lane.lst stat_file` to remove adapters and low-quality reads."

3. Add one or two sentences interpreting the key figures showing genus-level coverage improvement across avian orders/families.
Response: We have added a sentence to the manuscript interpreting the significance of the improved genus-level coverage. The 102 newly sequenced genera effectively fill previous gaps in the avian genomic map, and an increase of 4.43% in total genus coverage significantly enhances the resolution of the avian tree of life, providing a more robust foundation for exploring evolutionary traits across diverse lineages.

Reviewer #3:
In this paper, the B10K Project reports the adoption of low-input sequencing strategies to reduce costs while improving assembly quality compared with their earlier generation of order- and family-level avian genomes. Using primarily stLFR, complemented by 10X Genomics, the authors report the generation of 177 avian genomes representing 161 genera, including 102 newly sequenced at the genomic level. These genomes will be of broad use to the research community. The paper is well written and represents a significant data contribution. I have a few minor suggestions for the authors to consider.
Response: We appreciate your professional review work on our manuscript. We have carefully read your comments and addressed them in this revision.

Sampling and DNA extraction
From the Introduction, I understood that genomes were being generated primarily from historical sources (e.g., toe pads), but the mention of tissue and blood here suggests I may have misunderstood. Are you using the stLFR method for samples with limited amounts of high-molecular-weight DNA? It would be useful to clarify the source material used for DNA extraction.
Response: We apologize for any confusion. To clarify, our dataset is composed of mostly frozen materials and historical museum specimens. If properly preserved, we can still obtain relatively high-quality DNA from these samples (main fragments size

	>20k). We chose the stLFR method not only for its ability to generate long-range genomic information from HMW DNA but also because of its low-input requirement. We revised the manuscript and added the tissue type and library construction method to each sample in the Supplementary Table 1.<br><br>Library construction<br>Please provide a citation for the stLFR method. In addition, please elaborate on the 100 + 100 + 42 bp paired-end reads. Are these three separate runs on the DNBSEQ-T7 platform?<br>Response: We have added the citation for stLFR technology (Wang et al., 2019) to the manuscript. And the 100 + 100 + 42 bp format does not represent three separate runs. Instead, it is a single, integrated paired-end sequencing run performed on the DNBSEQ-T7 platform. In this configuration, the first two 100 bp segments sequence the paired-end genomic inserts (Read 1 and Read 2), while the additional 42 bp segment is a dedicated read designed to capture the co-barcoding information essential for stLFR long-fragment reconstruction.<br><br>stLFR and 10X Genomics<br>Please provide additional information on how the stLFR data were transformed into 10X Genomics format to enable assembly. If possible, could the script used for this conversion be deposited on GitHub or a similar repository to allow others to adopt a similar strategy?<br>Response: We have revised the sentences to include the technical details and specific parameters. We have explicitly stated the use of the stlfr2supernova pipeline (https://github.com/BGI-Qingdao/stlfr2supernova_pipeline) for format conversion.<br><br>Gene structures and annotation<br>In the first line of this section, something appears to be missing (e.g., "…homology-based method for both the ???…").<br>Response: Thank you for pointing this out. We have corrected the typo in the revised manuscript.<br><br>The genome annotation strategy is very useful and will further enhance the value of these genomes. To enable other researchers to replicate these analyses and apply similar approaches to their own genomes, could the primary reference gene set (20,194 genes), the supplemental human gene set (20,169 genes), and the supplemental transcriptome gene set (5,257 transcripts) be made available on GitHub or a similar data archive?<br>Response: We agree that making these gene sets accessible is crucial for the reproducibility of our analyses and for the broader research community. The primary reference gene set (20,194 genes), the supplemental human gene set (20,169 genes), and the supplemental transcriptome gene set (5,257 transcripts) can be accessed and downloaded via our GitHub repository via (https://github.com/B10KGenomes/annotation/tree/master/Gene_Annotation/0.homolog_data). And, we also have explicitly added this link in the relevant section to ensure easy navigation for future users.
**Additional Information:**	
**Question**	**Response**
Are you submitting this manuscript to a special series or article collection?	No
**Experimental design and statistics**<br><br><br>Full details of the experimental design and statistical methods used should be given in the Methods section, as detailed in our Minimum Standards Reporting Checklist. Information essential to interpreting the data presented should be made available	Yes

Powered by Editorial Manager® and ProduXion Manager® from Aries Systems Corporation

in the figure legends.<br><br>Have you included all the information requested in your manuscript?	
**Resources**<br><br>A description of all resources used, including antibodies, cell lines, animals and software tools, with enough information to allow them to be uniquely identified, should be included in the Methods section. Authors are strongly encouraged to cite Research Resource Identifiers (RRIDs) for antibodies, model organisms and tools, where possible.<br><br>Have you included the information requested as detailed in our Minimum Standards Reporting Checklist?	Yes
**Availability of data and materials**<br><br>All datasets and code on which the conclusions of the paper rely must be either included in your submission or deposited in publicly available repositories (where available and ethically appropriate), referencing such data using a unique identifier in the references and in the “Availability of Data and Materials” section of your manuscript.<br><br>Have you have met the above requirement as detailed in our Minimum Standards Reporting Checklist?	Yes
GigaScience has policies and guidelines in place for the use of generative AI-writing tools such as ChatGPT. If you have used such writing tools to assist with writing the manuscript this must be declared and cited in the text. Authors should not list AI-writing tools and other AI-assisted technologies as an author or co-author and should acknowledge that they are fully responsible for text generated or refined by AI-writing	No

tools.<p><br><br>A summary of use (particularly in the introduction or among methods) needs to be included at the end of the paper, and the outputs should also be included as a supplementary file hosted in GigaDB or other open repositories. Please read our guidelines for more information. <p><br><br>By submitting to GigaScience, you are aware of the journal's AI-writing tools policy, and if you have declared use of such tools below, you have acknowledged this where appropriate in your manuscript and have made a summary of use and outputs available. <p><br><b>AI-assisted writing tools have been used in the preparation of this manuscript?	

Manuscript

Click here to access/download;Manuscript;Manuscript_revised-OCRIDadded.docx

Draft assemblies for 177 bird species enhance genus-level coverage

Guangji Chen[1,*], Shuang Wang[1,*], Daniel Bilyeli Øksnebjerg[2,*], Sascha Dreyer Nielsen[2,*], Wei Dai[3,*], Wei Jiang[4], Jing Liang[1], Wei Han[1], Chengran Zhou[3], Qiye Li[4,5], Bent Petersen[2,6], Ara Monadjem[7,8], Zamekile D. Bhembe[7], Machawe Maphalala[7], Diego Ocampo[9,10], Luis Sandoval[11,12], Jörns Fickel[13,14], Alex D. Greenwood[15,16], Claudia A. Szentiks[15], Marco Roller[17,18,19], Sharon M. Birks[20], Adam D. Leaché[20,21], Alejandro Rico-Guevara[20,21], Jérôme Fuchs[22], Nguyen Tran Vy[23], Christina Hvilsom[24], Juliana Andrea Berner[24], Jan Terje Lifjeld[25], Arild Johnsen[25], Lars Erik Johannessen[25], Kim Labuschagne[26], Knud Andreas Jønsson[27,28], Martin Irestedt[28], Andrew Hart Reeve[27,28], Leo Joseph[29], Olof Hellgren[30], Robb T. Brumfield[31,32], Theresa M. Burg[33], Juan Carlos Illera[34], Alexandre Aleixo[35,36], Ben Smit[37], Frank E. Rheindt[38], Jessica Lee[39], Isao Nishiumi[40], Javier Quesada[41], John P. Dumbacher[42], Manuel Schweizer[43,44], Michael J. Andersen[45], Christopher C. Witt[45], Richard A. Phillips[46], Richard Prum[47], Kristof Zyskowski[48], Steven M. Goodman[49], Marie Jeanne Raherilalao[50,51], Ulf Ottosson[52], Yahkat Barshep[52,53], Sam Ivande[52,54], Vojtěch Brlík[55], Emmanuel Okposio[56], Bonny Koane[57], Tri Haryoko[58], Erich D. Jarvis[59], Carsten Rahbek[60,61,62], Fumin Lei[63], Gary R. Graves[60,64], Shaohong Feng[1,65,#,] Peter A. Hosner[27,60,61,#], M. Thomas P. Gilbert[2,66,#], Guojie Zhang[1,#]

Affiliations:

1. Center for Evolutionary & Organismal Biology, Liangzhu Laboratory & Women's Hospital, Zhejiang University School of Medicine, Hangzhou, China
2. Center for Evolutionary Hologenomics, Globe Institute, University of Copenhagen, Copenhagen, Denmark
3. BGI Research, Wuhan, China
4. State Key Laboratory of Genome and Multi-omics Technologies, & Shenzhen Key Laboratory of Forensics, BGI Research, Shenzhen 518083, China
5. College of Life Sciences, University of Chinese Academy of Sciences, Beijing, China
6. Senckenberg Natural History Collections Dresden, Dresden, Germany
7. Department of Zoology and Entomology, Mammal Research Institute, University of Pretoria, Hatfield, Pretoria, South Africa
8. Department of Biological Sciences, University of Eswatini, Kwaluseni, Eswatini
9. Museum of Vertebrate Zoology, University of California Berkeley, Berkeley, CA 94720 USA
10. Centro de Investigación en Biodiversidad y Ecología Tropical, Universidad de Costa Rica, San José, Costa Rica
11. Laboratorio de Ecología Urbana y Comunicación Animal, Escuela de Biología, Universidad de Costa Rica, San José, Costa Rica
12. Centro de Investigación en Biodiversidad y Ecología Tropical, Universidad de Costa Rica, San José, Costa Rica
13. Department of Evolutionary Genetics, Leibniz Institute for Zoo and Wildlife Research (IZW), Alfred-Kowalke-Strasse 17, 10315 Berlin, Germany
14. Institute of Biochemistry and Biology, Potsdam University, Karl-Liebknecht-Straße 24-25, 14476 Potsdam, Germany

15. Department Wildlife Diseases, Leibniz Institute for Zoo and Wildlife Research, Alfred-Kowalke-Strasse 17, 10315 Berlin, Germany
16. Department of Veterinary Medicine, Freie Universität Berlin, Berlin, Germany
17. Wilhelma Zoological-Botanical Gardens Stuttgart, Wilhelma 13, D-70376 Stuttgart, Germany
18. Department of Animal Sciences, Division of Microbiology and Animal Hygiene, Faculty of AgriculturalScience, Georg-August-University, Burckhardtweg 2, D-37077 Göttingen, Germany
19. Institute for Microbiology, University of Veterinary Medicine Hannover, Foundation, Bischofsholer Damm15, D-30173 Hannover, Germany
20. Burke Museum of Natural History and Culture, University of Washington, Seattle, Washington, USA
21. Department of Biology, University of Washington, Seattle, WA 98105, USA
22. Institut de Systématique, Evolution, Biodiversité (ISYEB), Muséum national d'Histoire naturelle, Paris, France
23. Institute of Tropical Biology, VAST, Ho Chi Minh City, Vietnam
24. Copenhagen Zoo, Copenhagen, Denmark
25. Natural History Museum, University of Oslo, Norway
26. South African National Biodiversity Institute(SANBI), Pretoria, SouthAfrica
27. Natural History Museum of Denmark, University of Copenhagen, Copenhagen, Denmark
28. Department of Bioinformatics and Genetics, Swedish Museum of Natural History, Stockholm, Sweden
29. Australian National Wildlife Collection, National Research Collections Australia, CSIRO, Canberra, Australia
30. Department of Biology, Lund University, Lund, Sweden
31. Museum of Natural Science, Louisiana State University, Baton Rouge, LA 70803, USA.
32. Department of Biological Sciences, Louisiana State University, Baton Rouge, LA 70803, USA.
33. Department of Biological Sciences, University of Lethbridge, Lethbridge, AB, T1K 3M4, Canada
34. Biodiversity Research Institute (CSIC-Oviedo University-Principality of Asturias), University of Oviedo, Campus of Mieres, E-33006 Mieres, Asturias, Spain
35. Museu Paraense Emílio Goeldi, Belém, PA, Brazil
36. Vale Institute of Technology, Belém, PA, Brazil
37. Department of Zoology and Entomology, Rhodes University, Makhanda, South Africa
38. Department of Biological Sciences, National University of Singapore, Singapore 117558, Singapore
39. Mandai Nature, Mandai, Singapore
40. Department of Zoology, National Museum of Nature and Science, Tokyo, Japan
41. Department of Vertebrates, Natural Sciences Museum of Barcelona, Barcelona, Spain
42. Ornithology & Mammalogy Department, California Academy of Sciences, San Francisco, CA 94118, United States
43. Natural History Museum Bern, Bern, Switzerland
44. Division of Population Genetics, Institute of Ecology and Evolution, University of Bern, Bern, Switzerland
45. Museum of Southwestern Biology, University of New Mexico, New Mexico, United States of America

46. British Antarctic Survey (BAS), Natural Environment Research Council (NERC), High Cross, Madingley Road, CB3 0ET Cambridge, United Kingdom
47. Department of Ecology and Evolutionary Biology, and Peabody Museum of Natural History, Yale University, New Haven, CT, USA
48. Division of Vertebrate Zoology, Peabody Museum of Natural History, Yale University, New Haven, CT USA
49. Field Museum of Natural History, Chicago, IL 60605, USA
50. Association Vahatra, Antananarivo, Madagascar
51. Mention Zoologie et Biodiversité Animale, Université d'Antananarivo, Madagascar
52. A.P. Leventis Ornithological Research Institute (APLORI), Centre of Excellence, University of Jos Biological Conservatory, Jos, Nigeria
53. Department of Zoology, University of Jos, Jos, Nigeria
54. Global Center for Species Survival, Indianapolis Zoo, 1200 West Washington St. Indianapolis, IN 46222, United States
55. Department of Ecology, Charles University, Prague, Czech Republic
56. Department of Biology, California State University, Fresno, California, USA
57. New Guinea Binatang Research Centre, Madang, Papua New Guinea.
58. Museum Zoologicum Bogoriense, Research Center for Biosystematics and Evolution, National Research and Innovation Agency (BRIN), Cibinong, 16911, Indonesia
59. The Vertebrate Genome Laboratory, The Rockefeller University, New York, USA
60. Center for Macroecology, Evolution and Climate, Globe Institute, University of Copenhagen, Copenhagen, Denmark
61. Center for Global Mountain Biodiversity, Globe Institute, University of Copenhagen, Copenhagen, Denmark
62. Department of Biology, University of Southern Denmark, 5230 Odense M, Denmark
63. Institute of Zoology, Chinese Academy of Sciences, Beijing, China
64. Department of Vertebrate Zoology, National Museum of Natural History, Smithsonian Institution, Washington DC, USA
65. Department of General Surgery of Sir Run Run Shaw Hospital, Zhejiang University School of Medicine, Hangzhou, China
66. University Museum, NTNU, Trondheim, Norway

* contributed equally
# Corresponding author

ORCIDs: Guangji Chen [0000-0002-9441-1155]; Shuang Wang [0009-0008-9451-5684]; Sascha Dreyer Nielsen [0000-0003-2485-9314]; Wei Dai [0000-0001-9286-759X]; Chengran Zhou [0000-0002-9468-5973]; Qiye Li [0000-0002-5993-0312]; Bent Petersen [0000-0002-2472-8317]; Ara Monadjem [0000-0003-1906-4023]; Zamekile D. Bhembe [0009-0006-5354-5751]; Machawe Maphalala [0000-0002-9304-1391]; Luis Sandoval [0000-0002-0793-6747]; Jörns Fickel [0000-0002-0593-5820]; Alex D. Greenwood [0000-0002-8249-1565]; Adam D. Leaché [0000-0001-8929-6300]; Alejandro Rico-Guevaera [0000-0003-4067-5312]; Christina Hvilsom [0000-0001-

7870-6888]; Arild Johnsen [0000-0003-4864-6284]; Lars Erik Johannessen [0000-0001-5981-9190]; Kim Labuschagne [0000-0003-2784-4767]; Knud Andreas Jønsson [0000-0002-1875-9504]; Martin Irestedt [0000-0003-1680-6861]; Leo Joseph [0000-0001-7564-1978]; Robb T. Brumfield [0000-0003-2307-0688]; Theresa M. Burg [0000-0001-5096-3479]; Juan Carlos Illera [0000-0002-4389-0264]; Alexandre Aleixo [0000-0002-7816-9725]; Frank E. Rheindt [0000-0001-8946-7085]; Jessica Lee [0000-0003-0757-4237]; Javier Quesada [0000-0002-6010-8473]; Manuel Schweizer [0000-0002-7555-8450]; Michael J. Andersen [0000-0002-7220-5588]; Christopher C. Witt [0000-0003-2781-1543]; Richard A. Phillips [0000-0002-0208-1444]; Richard Prum [0000-0002-4741-7132]; Kristof Zyskowski [0000-0002-5680-6412]; Marie Jeanne Raherilalao [0000-0002-8618-7157]; Ulf Ottosson [0000-0001-7914-0484]; Sam Ivande [0000-0003-4949-1376]; Vojtěch Brlík [0000-0002-7902-8123]; Bonny Koane [0000-0001-6770-5126]; Tri Haryoko [0000-0002-8549-3662]; Erich D. Jarvis [0000-0001-8931-5049]; Carsten Rahbek [0000-0003-4585-0300]; Fumin Lei [0000-0001-9920-8167]; Gary R. Graves [0000-0003-1406-5246]; Shaohong Feng [0000-0002-2462-7348]; Peter A. Hosner [0000-0001-7499-6224]; M. Thomas P. Gilbert [0000-0002-5805-7195]; Guojie Zhang [0000-0001-6860-1521]

Abstract

Background: With over 10,000 recognized species, birds constitute one of the most diverse and widely distributed vertebrate groups. Although avian genomics has advanced rapidly over the past decade, substantial gaps remain across the global avifauna. Filling these gaps is essential for understanding macroevolutionary patterns, population structure, and the molecular basis of ecological and behavioral diversity. Worldwide museum collections represent invaluable resources for filling these gaps, yet the typically degraded DNA and limited quantities from historical specimens have posed significant challenges for generating high-quality genome assemblies. **Results**: Here, the Bird Genome 10K (B10K) Project adopted low-input sequencing strategies that reduce costs while improving assembly quality compared with earlier order- and family-level genomes. Using mainly stLFR, complemented by 10X Genomics and standard next-generation sequencing, we assembled 177 avian genomes from museum specimens and tissue collections representing 161 genera, including 102 newly sequenced at the genomic level. The assemblies average ~1.2 Gb in size, with scaffold N50 = 8.03 Mb, contig N50 = 120 kb, 93% BUSCO completeness, and Merqury QV score of 56. **Conclusions**: These genomes greatly expand avian taxonomic coverage and demonstrate the efficiency of low-input sequencing for generating high-quality assemblies from limited and often degraded material sourced from museum specimens. This resource provides a foundation for comparative genomics, conservation genetics, and evolutionary studies across the avian tree of life.

Keywords: birds; genome sequencing; biodiversity.

Context

Birds, derived from theropod dinosaurs, constitute one of the most species-rich and widely

distributed vertebrate radiations, encompassing more than 10,000 species and over 2,000 genera that inhabit nearly every ecosystem on Earth[1,2]. Over the past decade, facilitated by international efforts such as the Bird 10,000 Genomes (B10K) Project[3,4] and the Vertebrate Genomes Project, avian genome resources have expanded rapidly (**Figure 1**), providing unprecedented opportunities for comparative and functional genomics. This growth has been enabled not only by advances in sequencing and assembly technologies but also by the invaluable contribution of natural history museums and institutional biobanks, whose curated specimens have become indispensable for large-scale genome initiatives.

The B10K Project has achieved significant milestones over the past decade in generating genomic data for species representing order- and family-level diversity across the avian tree of life[3–6]. However, genus- and species-level coverage remains highly uneven. Several genera, such as *Aphelocoma*, *Falco*, *Gallus*, *Anas*, and *Haemorhous* are represented by dozens of assemblies, whereas many lineages remain underrepresented due to limited access to fresh tissues or vouchered samples. For numerous species, especially those inhabiting remote regions or represented primarily by historical specimens, natural history collections remain the only feasible sources of DNA. However, these collections are often geographically dispersed and difficult to access for genomic research due to limitations in both the quality (high-molecular-weight genomic DNA) and quantity of available genetic material. As a result, molecular-level understanding of these taxa has long been constrained by the scarcity of genomic resources.

Recent advances in low-input sequencing and museomics technologies (e.g., stLFR[7]) are gradually bridging this gap, allowing for the assembly of genomes from previously inaccessible samples. B10K Project has strategically adopted these low-input sequencing methods, reducing costs while improving genome assembly quality compared to earlier order- and family-level studies. Here, using stLFR technologies, we report draft genome assemblies for 177 bird species representing 161 genera, including 102 genera newly covered at the genomic level (**Figure 2**). These newly sequenced genera increased by 4.43% the genus-level coverage of extant bird species, providing a more continuous phylogenetic framework for comparative genomics across the avian tree of life. These assemblies substantially enrich the genomic resources available for avian research, offering new insights into the genetic diversity and evolutionary history of this remarkable vertebrate group.

Materials and Methods

Sampling, sequencing, assembly, and annotation

DNA extraction

Genomic DNA was extracted from 177 avian species, comprising both frozen materials and historical museum specimens, as detailed in **Supplementary Table 1**. Specifically, 80.8% of the samples (n = 143) were collected before 2020 with many challenging historical sources that necessitated the low-input sequencing strategy. All DNA extractions were performed using the ‘MagMAX DNA Multi-Sample Ultra 2.0` kit (Thermo Fisher) following the manufacturer’s guidelines. For post-lysis, RNA degradation was performed by incubating the lysate with RNase A

at 37°C for 5 minutes. Subsequently, genomic DNA was isolated using a bead-based purification method, utilizing 440 µL of DNA Binding Bead Mix per sample. The automated isolation process was executed on the Kingfisher Duo Prime system (Thermo Fisher Scientific), following the standard MMX_Ultra2_Cell_Tissue_96_Duo protocol. Quality control was performed on the 2200 Tapestation System, and all DNA extractions were stored at -80°C until library construction.

Library construction and sequencing

Linked-Reads Library

The selection of sequencing technologies was strategically determined by the interplay between DNA integrity and the progressive availability of sequencing platforms throughout the project. The majority of the DNA samples (n = 161) were prepared using the stLFR (Single-Tube Long Fragment Reads) technology, which served as our primary pipeline due to its ability to generate high-quality linked-read data with 10 ng of input DNA with fragment sizes exceeding 10 kb. During the early phase of this study, 13 DNA samples which exhibited exceptional DNA integrity (main fragment sizes > 40 kb) were prepared using the 10X Genomics Chromium library system.

For the 13 samples prepared using the 10X Genomics Chromium library system, high-molecular-weight genomic DNA was processed with the Chromium Genome Library Kit (10X Genomics, Pleasanton, USA) to generate barcoded linked-read libraries. To ensure compatibility and sequencing efficiency across platforms, the libraries were adapted for sequencing on the BGISEQ-500 platform (BGI-Shenzhen, China) following in-house optimization to produce 150 bp paired-end reads in total 1.6 Tb raw sequencing reads (on average ~ 103x).

For the 161 samples prepared using stLFR (Single-Tube Long Fragment Reads) technology (MGI), high-molecular-weight genomic DNA was processed following the manufacturer's barcoding protocol, which enables the physical linkage of short reads derived from the same long DNA fragment. The barcoded libraries were sequenced on the DNBSEQ-T7 platform (MGI, Shenzhen, China) with PE100+42 mode, which included two 100 bp reads for the genomic inserts and a dedicated 42 bp read to capture the co-barcoding sequences essential for the stLFR method[7], and produced in total 26.3 Tb raw sequencing reads (on average ~ 136x).

Standard Next-Generation Sequencing

For the remaining 3 samples with relative DNA degradation (fragment sizes < 10 kb) that failed to meet the requirements for linked-read library construction, standard short-read next-generation technology with approximately 500 ng input DNA was employed to ensure sufficient genomic coverage despite suboptimal sample quality. Sequencing libraries with an average insert size of approximately 350 bp were constructed according to the manufacturer's protocol and sequenced on the DNBSEQ-T1 platform (BGI-Shenzhen, China) to produce 150 bp paired-end reads in total 226 Gb raw sequencing reads (on average 63x).

Assembly and Statistics of genomes

stLFR and 10X Genomics

For the 161 samples sequenced using stLFR technology, raw data were initially transformed into 10X Genomics linked-reads format following the stlfr2supernova pipeline [8]. Subsequently, these 161 samples, together with 13 samples sequenced by 10X Genomics linked-reads technology, were preprocessed with SOAPfilter (v2.2, RRID:SCR_000689) using parameters `-i $insertsize -p -z -g 1 -M 2 -Q 10 lane.lst stat_file` to remove adapters and low-quality reads. The cleaned reads were then introduced into the Supernova software (v2.0.1; RRID:SCR_016756) [9] to assemble the genomes under the "pseudohap" mode. The assembly was executed with the command: `supernova run --id=ID --fastqs=./ --localcores=32 --localmem=300G --nopreflight `, followed by `supernova mkoutput --style=pseudohap --asmdir=./ID/outs/assembly --outprefix=output` to generate the final genomic sequences.

To ensure assembly quality, scaffolds containing more than 80% "N" bases were removed using housed Perl script, which typically resulted in the exclusion of approximately 2 scaffolds per assembly. Subsequently, gap filling was applied with Gapcloser (v1.12, RRID:SCR_015026) [10] by using the default parameters and the cleaned paired-end reads.

Standard Next-Generation technology

For the 3 samples sequenced by the standard Next-Generation technology, reads were cleaned by SOAPfilter (v2.2, RRID:SCR_000689) with previous parameters and assembled using the SOAPdenovo (v2.04, RRID:SCR_010752) [10] with a K-mer size of 23-mer following the B10K family-phased assembly strategy [4]. After removing scaffolds with "N" > 80%, GapCloser (v1.12, RRID:SCR_015026) [10] was used to close the intra-scaffold gaps with default parameters based on the cleaned paired-end reads.

Gene structures annotation

Annotation of protein-coding genes was conducted with a homology-based method for the 177 bird species following the pipeline implemented by the Bird 10,000 Genomes (B10K) consortium[4]. The reference gene set consists of the primary reference gene set (20,194 genes), the supplemental human gene set (20,169 genes), and the supplemental transcriptome gene set (5,257 transcripts), which could be accessed [11]. The protein sequences in the primary reference gene set were first aligned to each genome using TBLASTN (v2.2.26; RRID:SCR_011822) [12] with an e-value cut-off 1e-5, and multiple adjacent hits of the same query were connected by genBlastA (v1.0.4; RRID:SCR_020951) [13]. Homologous blocks with a length greater than 30% of the query protein length were retained. Each connected hit region was later extended to include its 2 kb upstream and downstream flanking regions, on which gene structure was predicted by Genewise (v2.4.1; RRID:SCR_015054) [14]. MUSCLE (v3.8.31; RRID:SCR_011812) [15] was then used to align the annotated protein against its reference protein. Predicted proteins with length ≥ 30 amino acids and an identity value ≥ 40% were retained. Pseudogenes (annotated genes containing > 2 frameshifts or > 1 premature stop codon) and retrogenes were further removed. Next, gene models that overlapped in > 40% of their coding sequence were clustered into one group and the one with the highest identity to the reference proteins was retained to form a non-

redundant gene set for each species. Two supplemental gene sets were also used for homology-based gene prediction for these newly released assemblies as above, but only the newly annotated loci from these supplemental sets were kept. Finally, all candidate annotated genes that had > 10 duplications were removed. Among the protein-coding annotations of these 177 avian species, an average of 89.53% were annotated using the core avian gene set, 8.43% were supported by the transcriptome gene set, and 2.03% by the human gene set.

Phylogenetic information

Phylogenetic information for the 177 newly released avian species was derived from the recently large-scale avian phylogenies[16], and the tree was pruned to match the dataset using the R package ape[17], and visualized using the R packages ggtree[18].

Data Validation and quality control

Sample taxonomic verification

Voucher specimens were verified against reference collections. Mitogenomes were BLAST searched against references in the NCBI database. If no reference mitogenome was available, we checked NCBI for commonly sequenced mitochondrial genes (*COI*, *ND2*, *CYB*) as a reference to verify sequence authenticity. For genomes that lacked a mitochondrial assembly, which was frequent when blood was the source tissue, we used a collection of nuclear markers well-represented on NCBI for birds (*FGB, GAPDH, MB, MUSK, ODC, RAG1, TGFb2*).

Contamination screening

Genome assemblies were screened for contamination using the NCBI Foreign Contamination Screen (FCS-GX) pipeline [19]. Adapter and contaminant sequences were detected using the FCS adaptor module, and contaminant scaffolds were removed or trimmed using the FCS cleaning module with the default parameters.

Quality control on sequencing reads

Quality control steps for raw reads before assembly using the SOAPfilter2 package (v2.2, RRID:SCR_000689) were: Remove reads with more than 10% of N bases; Remove reads with more than 40% low-quality bases (Phred score < = 10); Remove reads with undersized insert size; Filter out the PCR duplicates.

Statistics of genome assembly

Assembly quality of 177 assemblies was assessed by contig N50, scaffold N50, and total assembly length. Genome completeness was measured with Compleasm (v0.2.7, RRID:SCR_026370)[20] using aves_odb12 as the reference gene set for these species. Three standard categories of BUSCO results were assessed as follows: Complete and single-copy BUSCOs (S), Complete and duplicated BUSCOs (D), Fragmented BUSCOs (F), The Complete and single-copy BUSCOs (S) and duplicated BUSCOs (D) hits were combined to assess the degree of genome completeness. Furthermore, to estimate the base-level accuracy and consensus quality, Merqury (v1.3, RRID:SCR_022964)[21] was utilized to calculate the Consensus Quality Value (QV) and k-mer completeness by comparing the assemblies against the original high-quality sequencing reads in

a k-mer-based, reference-independent manner.

Genomic quality

The genome assemblies of the 177 avian genomes have an average genome size of ~ 1.2 Gb, with average scaffold N50 = 8.03 Mb, and contig N50 = 120 kb (**Figure 3**). Overall, the assembly quality shows an improvement compared with previous datasets generated at the order and family levels, largely due to the adoption of low-input sequencing strategies. Only 2.5% of the core genes in aves_odb12 could not be predicted on these newly released genomes (ranging from 0.2% to 10.2%), suggesting that the completeness of these genomes was suitable for most comparative genome analyses. An average of 93% complete core genes in these 177 avian genomes was comparable to that of the previously published genomes (**Figure 4**). On average, only 4.4% of the core genes were partly annotated for the 177 avian genomes. Furthermore, k-mer-based evaluation yielded an average Merqury QV score of 56 (**Supplementary Table 1**), indicating a relatively high consensus accuracy at the base level.

Using the homologous annotation method, the 177 genomes were predicted to contain an average of 16,461 protein-coding genes, similar to previously published bird genomes[4]. The average gene length and coding sequence length are ~15 and 1.26 kb, respectively. Genes contained on average ~7 exons, with an average length of 172 bp and an average intron length of 2.17 kb (**Supplementary Table 1**). Across most annotation and assembly metrics, there was no significant difference between the two Linked-Reads technologies (10X Genomics and stLFR), both of which outperformed the standard short-read next-generation sequencing method (**Figure 5**). Investigations across different avian lineages showed that Piciformes have a distinctly higher GC content with larger genome size variation (**Supplementary Table 1** and **Figure 6**), which is consistent with prior research [22].

Re-use potential

We created this dataset to support future research on avian biology. While it will be useful for broad avian evolutionary studies, we expect its most significant value will be in conservation genetics and the practical management of the threatened species. Specifically, these genomic resources enable the assessments of genetic diversity in endangered birds, providing essential data to identify inbreeding depression or genetic bottlenecks. The dataset facilitates precise population genetic structure analysis and the mapping of gene flow, which are critical for designing effective translocation or captive breeding programs. Furthermore, we expect its most significant value will be in studying how global change affects the loss of genomic diversity in avian species, which is a necessary input to cope with the current challenges of the Anthropocene[23,24].

However, since our gene prediction primarily relies on a homology-based pipeline, there may be reduced sensitivity in identifying lineage-specific genes that lack close homologs in reference databases. Additionally, potential gene fragmentation effects may exist in regions where the

assembly is less contiguous, which is a common trade-off in homology-based structural annotations. It should be noted that while this study provides the protein-coding gene annotations, the identification and characterization of non-coding RNAs (ncRNAs), such as miRNAs and lncRNAs, were not conducted in the current study. Future studies leveraging small RNA sequencing and specialized bioinformatic pipelines will be necessary to fully capture the regulatory landscape of these birds' genomes.

Despite these, the potential uses include population genomics, mapping genetic structure and gene flow, identifying hybrid zones, studying local adaptation, and investigating disease ecology. The quality of the genomes released here represents a level that was feasible at the time of this project, given the financial, technical, and biosample constraints available to the project. Nevertheless, we believe the quality will be suitable for many of the above possible end uses.

Supplementary data

Supplementary Table is available online.

Data Availability

Genome assemblies, and annotations of the 177 species generated in this study have been deposited under the BioProject accession number PRJEB102986 (ENA). Sample information for each genome and the genome statistics can also be viewed online at B10K website[25]. Code to run the genome assembly pipeline can be found at [8] and the corresponding genome annotation pipeline [11]. All additional supporting data are available in the GigaScience repository, GigaDB [26].

Abbreviations

AviList: The Global Avian Checklist; NCBI: National Center for Biotechnology Information; stLFR: Single-Tube Long Fragment Reads; B10K: The Bird 10,000 Genomes (B10K) Project; BUSCOs: Benchmarking Universal Single-Copy Orthologs; NGDC: National Genomics Data Center.

Declarations

All field sampling efforts followed appropriate local permission structures, laws, and regulations, including the Nagoya Protocol and Institutional Review Boards where they apply. All tissue samples were received and processed at Globe Institute, University of Copenhagen, which is certified to receive and process biological tissues under a permit for Animal By-Products DK-123-oth-905637 issued by The Danish Veterinary and Food Administration. CITES samples received from outside the European Union are under the CITES Scientific Exchange Exemption, registration number DK 014 issued by the Danish Environmental Protection Agency.

Competing interests

The authors declare no competing interests.

Funding

This work was supported by the National Key Research and Development Program of China (no. 2024YFA1802500) to G.Z.; the National Natural Science Foundation of China grant (no. 32422009) and the National Key Research and Development Program of China grant (no. 2023YFA1800500) to S.F.; the Postdoctoral Fellowship Program of CPSF Grant (no. GZB2025059) to G.C.; Danish National Research Foundation (DNRF143) to M.T.P.G.; Independent Research Fund Denmark 1054-00039B to P.A.H., who was also supported by a research grant (25925) from VILLUM FONDEN; J.C.I. was funded by two research grants from the Spanish Ministry of Science, Innovation and Universities and the European Regional Development Fund (PGC2018-097575-B-I00; PID2022-140091NB-I00); K.A.J acknowledges support from the Villum Foundation (Young Investigator Programme, project No. 15560, K.A.J.), the Carlsberg Foundation (CF15-0079, K.A.J.).

Authors' contributions

G.Z., M.T.P.G., E.D.J., P.A.H., C.R., F.L., G.R.G. and S.F. conceived of the study. A.M., Z.D.B., M.M, D.O., L.S., J.Fi., N.T.V., A.D.G., C.A.S., M.R., S.M.B., A.D.L., A.R., J.Fu., C.H., J.A.B., J.T.L., A.J., L.E.J., K.L., K.A.J., A.H.R., M.I., L.J., O.H., R.B., T.M.B., J.C.I., A.A., B.S., F.R., Je.L., I.N., J.Q., J.P.D., M.S., M.A., C.C.W., R.A.Ph., R.Pr., K.Z., S.M.G., M.J.R., U.O., Y.B., S.I., V.B., E.O., B.K., T.H., F.L., G.R.G. and P.A.H. provided genetic samples from fieldwork. G.C., S.W., D.B.O., S.D.N., W.D., W.J., Ji.L., W.H., C.Z., Q.L. and B.P. performed sample acquisition, lab work, and bioinformatics. G.C., S.F., P.A.H., M.T.P.G. and G.Z. drafted the manuscript, which was edited and approved by all authors.

Acknowledgements

The authors would like to thank the various field workers, museums, universities and other genetic resource archives, permitting officials, and other support and administrative staff without whom sample collection programs could not function: Natural History Museum Denmark (NHMD), University of Copenhagen, Smithsonian National Museum of Natural History (USNM), Natural History Museum of Oslo (NHMO), Senckenberg Natural History Collections Dresden (SNSD), University of Eswatini, University of Pretoria, CIBET-Universidad de Costa Rica, Leibniz Institute for Zoo and Wildlife Research (IZW), Freie Universität Berlin (FUB), Wilhelma Zoological-Botanical Gardens Stuttgart, University of Veterinary Medicine Hannover (TiHo Hannover), Burke Museum of Natural History and Culture (UWBM), University of Washington (UW), Muséum national d'Histoire naturelle (MNHN), Copenhagen Zoo, South African National Biodiversity Institute (SANBI), Swedish Museum of Natural History (NRM), Australian National Wildlife Collection (CSIRO ANWC), Lund University, Louisiana State University (LSU), University of Lethbridge (ULeth), Museu Paraense Emílio Goeldi (MPEG), Finnish Museum of Natural History (LUOMUS), University of Helsinki, Rhodes University, National University of Singapore (NUS), University of Oviedo (UOV), Mandai Nature, National Museum of Nature and Science Japan

(NSMT), Natural Sciences Museum of Barcelona (MZB), California Academy of Sciences (CAS), Natural History Museum Bern (NMBE), University of Bern, Museum of Southwestern Biology (MSB), University of New Mexico (UNM), British Antarctic Survey (BAS), Yale Peabody Museum (YPM), Field Museum of Natural History (FMNH), Université d'Antananarivo, Association Vahatra, A.P. Leventis Ornithological Research Institute (APLORI), University of Jos, Indianapolis Zoo, Charles University, Institute of Zoology, Chinese Academy of Sciences (IOZ), and NTNU University Museum. We also thank the Zhejiang Lab, the Information Technology Center of ZheJiang University and China Mobile Zhejiang Co., Ltd (Hangzhou Branch) for providing computational resources.

Reference

1. Howard R, Moore A, Dickinson EC, Remsen JV. The Howard and Moore Complete Checklist of the Birds of the World. Aves Press;

2. Rheindt FE, Donald PF, Donsker DB, Gerbracht JA, Iliff MJ, Lepage D, et al. AviList: a unified global bird checklist. *Biodivers Conserv*. Springer Science and Business Media LLC; 2025; doi: 10.1007/s10531-025-03120-y.

3. Zhang G, Li C, Li Q, Li B, Larkin DM, Lee C, et al. Comparative genomics reveals insights into avian genome evolution and adaptation. *Science*. American Association for the Advancement of Science (AAAS); 2014; doi: 10.1126/science.1251385.

4. Feng S, Stiller J, Deng Y, Armstrong J, Fang Q, Reeve AH, et al. Dense sampling of bird diversity increases power of comparative genomics. *Nature*. 2020; doi: 10.1038/s41586-020-2873-9.

5. Jarvis ED, Mirarab S, Aberer AJ, Li B, Houde P, Li C, et al. Whole-genome analyses resolve early branches in the tree of life of modern birds. *Science*. 2014; doi: 10.1126/science.1253451.

6. Stiller J, Feng S, Chowdhury A-A, Rivas-González I, Duchêne D, Fang Q, et al. Complexity of avian evolution revealed by family-level genomes. *Nature*. Nature Publishing Group UK; 2024; doi: 10.1038/s41586-024-07323-1.

7. Wang O, Cheng X, Drmanac R, Peters BA. A simple cost-effective method for whole-genome sequencing, haplotyping, and assembly. *Methods Mol Biol*. New York, NY: Springer US; 2023; doi: 10.1007/978-1-0716-2819-5_7.

8. A pipeline to de novo assemble the stLFR reads using Supernova Assembler. https://github.com/BGI-Qingdao/stlfr2supernova_pipeline Accessed 2026 Apr 13.

9. Weisenfeld NI, Kumar V, Shah P, Church DM, Jaffe DB. Direct determination of diploid genome sequences. *Genome Res*. 2017; doi: 10.1101/gr.214874.116.

10. Luo R, Liu B, Xie Y, Li Z, Huang W, Yuan J, et al. SOAPdenovo2: an empirically improved memory-

efficient short-read de novo assembler. *Gigascience*. 2012; doi: 10.1186/2047-217X-1-18.
11. Scripts used in the annotation of B10K genomes. https://github.com/B10KGenomes/annotation.
12. Altschul SF, Madden TL, Schäffer AA, Zhang J, Zhang Z, Miller W, et al. Gapped BLAST and PSI-BLAST: a new generation of protein database search programs. *Nucleic Acids Res*. 1997; doi: 10.1093/nar/25.17.3389.
13. She R, Chu JS-C, Wang K, Pei J, Chen N. GenBlastA: enabling BLAST to identify homologous gene sequences. *Genome Res*. 2009; doi: 10.1101/gr.082081.108.
14. Birney E, Clamp M, Durbin R. GeneWise and Genomewise. *Genome Res*. 2004; doi: 10.1101/gr.1865504.
15. Edgar RC. MUSCLE: multiple sequence alignment with high accuracy and high throughput. *Nucleic Acids Res*. 2004; doi: 10.1093/nar/gkh340.
16. Claramunt S, Sheard C, Brown JW, Cortés-Ramírez G, Cracraft J, Su MM, et al.. A new time tree of birds reveals the interplay between dispersal, geographic range size, and diversification. *Curr Biol*. Elsevier BV; 2025; doi: 10.1016/j.cub.2025.07.004.
17. Paradis E, Claude J, Strimmer K. APE: Analyses of Phylogenetics and Evolution in R language. *Bioinformatics*. Oxford University Press (OUP); 2004; doi: 10.1093/bioinformatics/btg412.
18. Yu G. Using ggtree to Visualize Data on Tree-Like Structures. *Curr Protoc Bioinformatics*. 2020; doi: 10.1002/cpbi.96.
19. Foreign Contamination Screening caller scripts and documentation. https://github.com/ncbi/fcs.
20. Huang N, Li H. compleasm: a faster and more accurate reimplementation of BUSCO. *Bioinformatics*. 2023; doi: 10.1093/bioinformatics/btad595.
21. Rhie A, Walenz BP, Koren S, Phillippy AM. Merqury: reference-free quality, completeness, and phasing assessment for genome assemblies. *Genome Biol*. 2020; doi: 10.1186/s13059-020-02134-9.
22. Kapusta A, Suh A. Evolution of bird genomes—a transposon's-eye view. *Ann N Y Acad Sci*. Wiley; 2017; doi: 10.1111/nyas.13295.
23. Cassin-Sackett L, Welch AJ, Venkatraman MX, Callicrate TE, Fleischer RC. The contribution of genomics to bird conservation. *Avian Genomics in Ecology and Evolution*. Cham: Springer International Publishing; doi: 10.1007/978-3-030-16477-5_10.
24. Bernatchez L, Ferchaud A-L, Berger CS, Venney CJ, Xuereb A. Genomics for monitoring and understanding species responses to global climate change. *Nat Rev Genet*. 2024; doi: 10.1038/s41576-023-00657-y.
25. B10K Website. https://b10k.com/.

26. Chen G, Wang S, Øksnebjerg D B, Nielsen S D, Dai W, Jiang W, et al. Supporting data for "Draft assemblies for 177 bird species enhance genus-level coverage." GigaScience Database. 2026; doi:10.5524/102772.
27. Bird species reference genomes in the NCBI database. https://www.ncbi.nlm.nih.gov/genome/browse/#!/aves/ Accessed 2025 Oct 11.
28. The IUCN Red List of Threatened Species. https://www.iucnredlist.org.

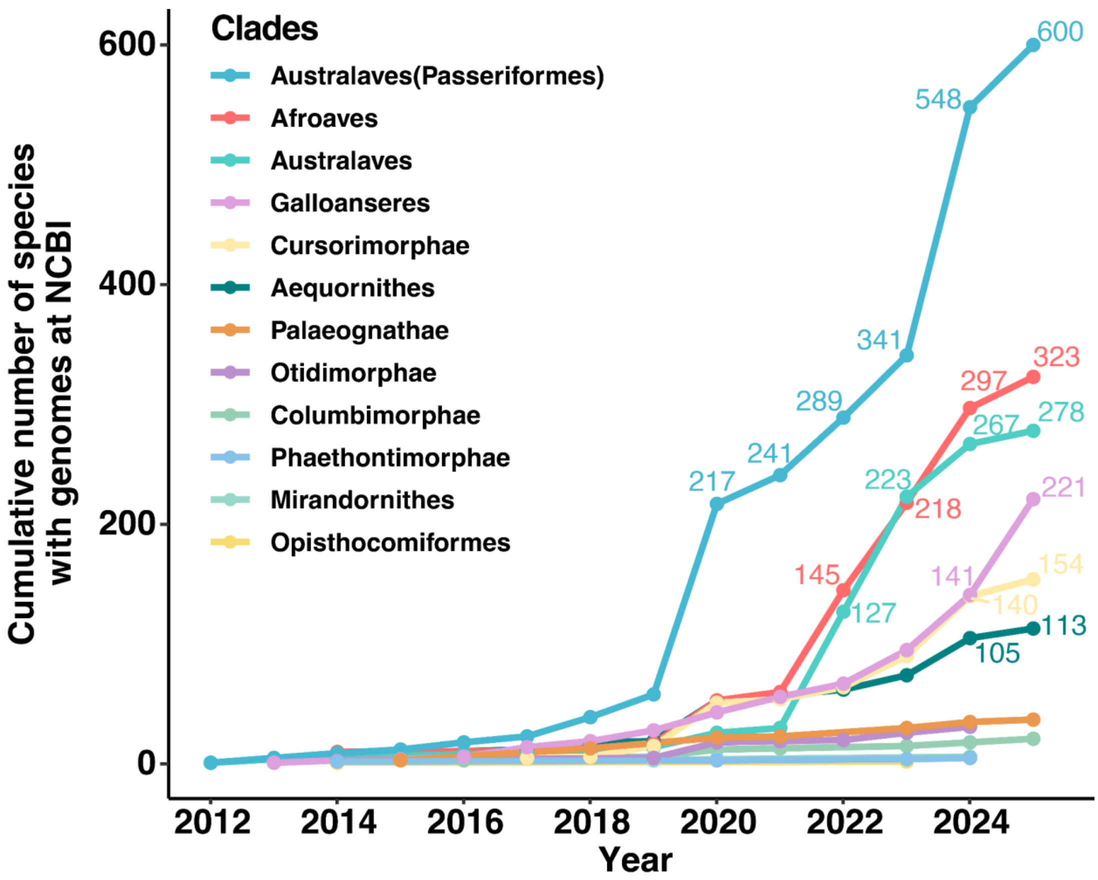

Figure 1. **Temporal accumulation of avian reference genomes in the NCBI database.** Cumulative number of bird species with reference genomes in the NCBI database (obtained from [27] on 11 October 2025). Data points are color-coded by taxonomic clades to illustrate the expanding genomic representation across different avian lineages.

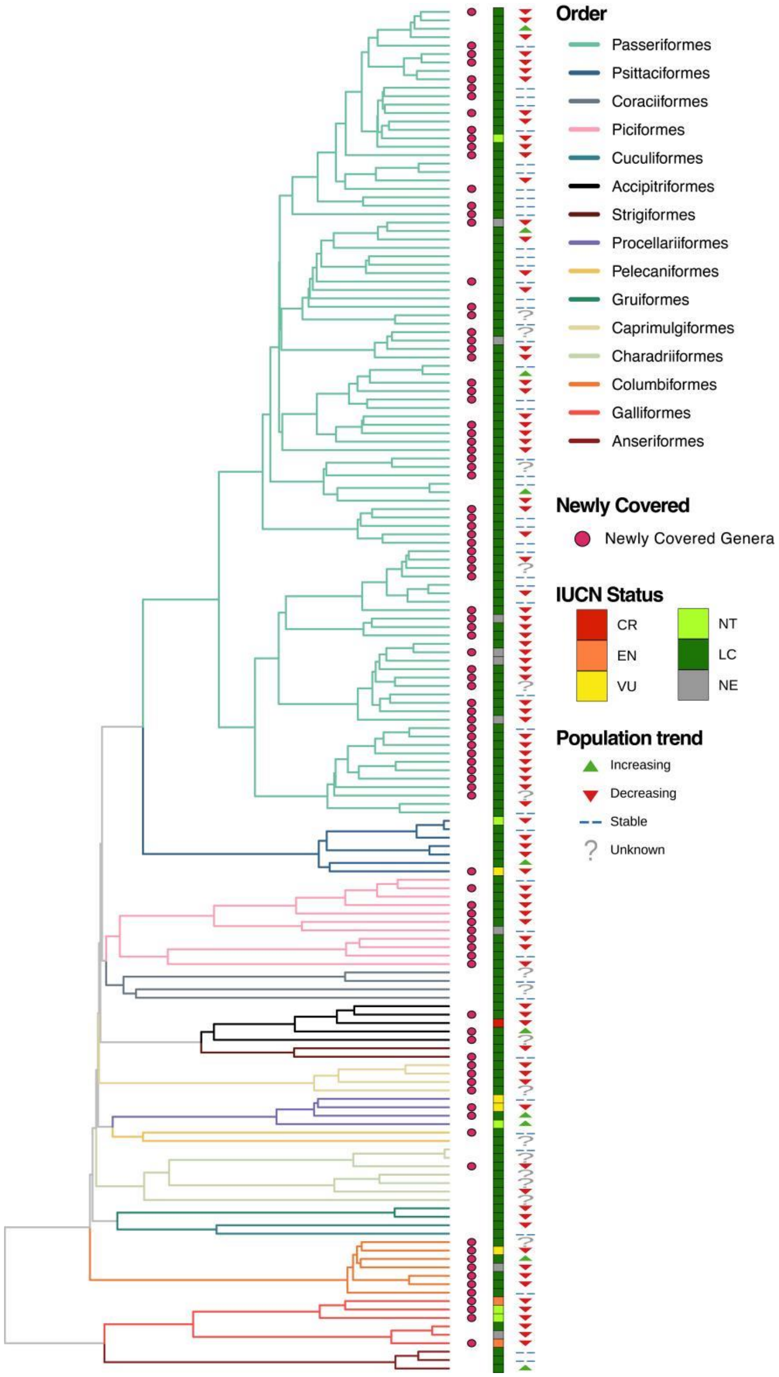

Figure 2. **Phylogenetic distribution, taxonomic representation, and conservation profiles of the 177 newly sequenced avian genomes.** Branches and internal clades are color-coded according to taxonomic orders. Red circles at the tips indicate newly covered genera. The color-coded strip represents the IUCN Red List categories (CR: Critically Endangered; EN: Endangered; VU: Vulnerable; NT: Near Threatened; LC: Least Concern; NE: Not Evaluated; obtained from [28]). And the symbols indicate the population trend, including increasing, decreasing, stable, and unknown.

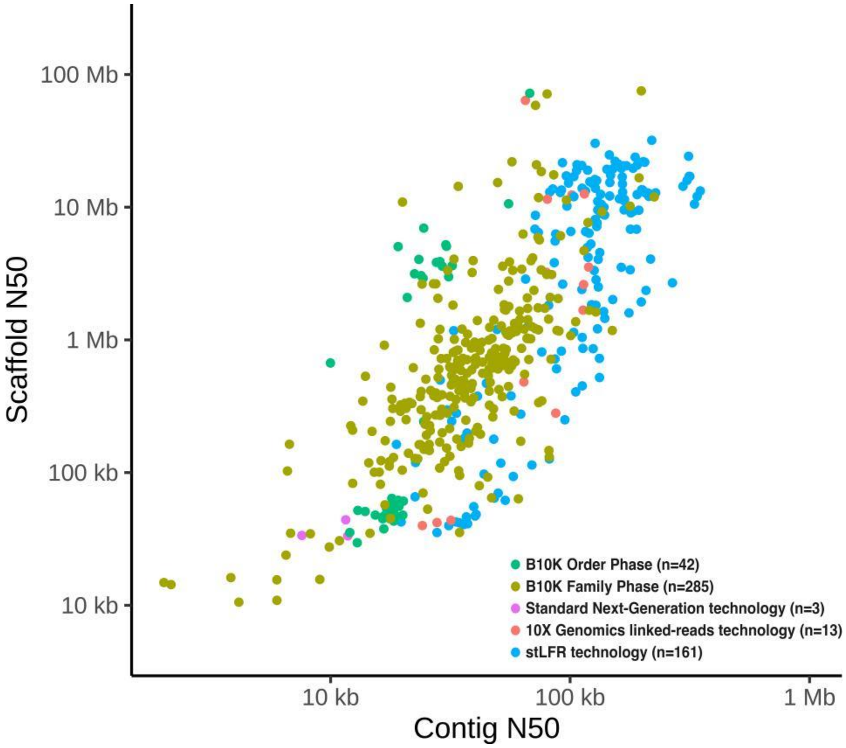

Figure 3. **Evaluation of assembly continuity for 504 avian genomes.** Scatter plot of Contig N50 versus Scaffold N50 metrics. Each dot represents a species. The dataset comprises 177 genomes from this study and 327 legacy genomes from the B10K project (Order Phase and Family Phase).

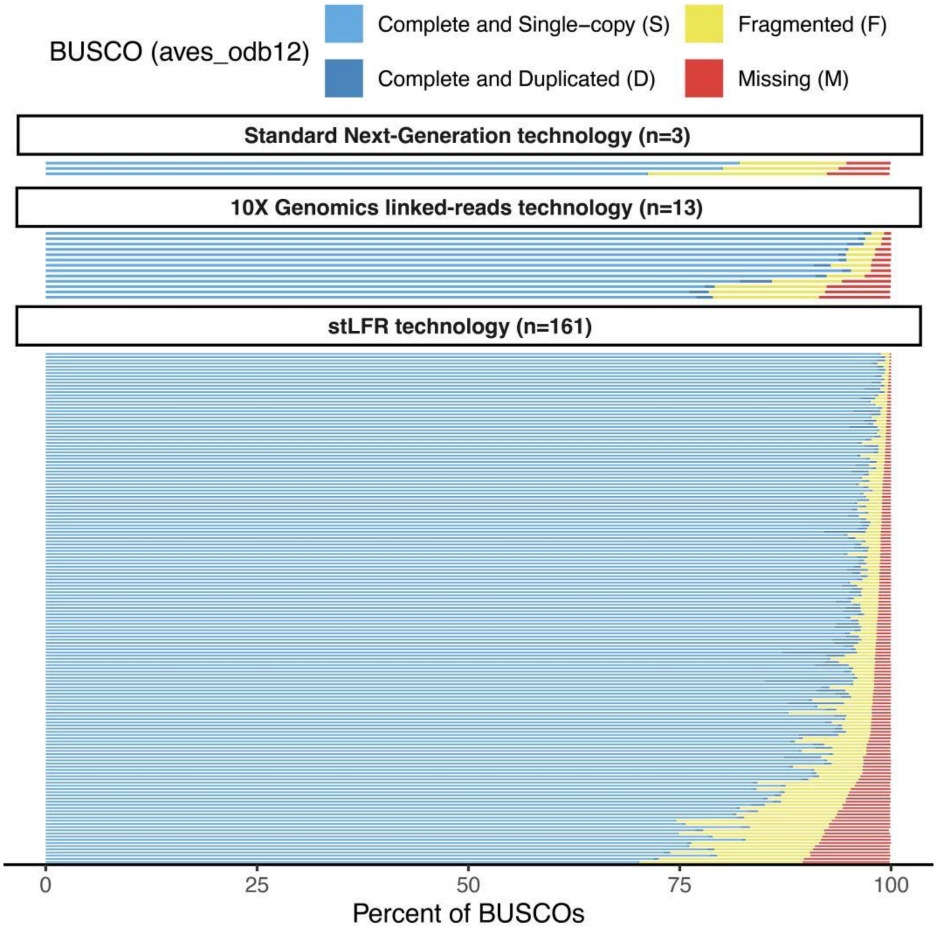

Figure 4. **Assessment of genome assembly completeness for 177 newly sequenced avian genomes.** Distribution of BUSCO scores (aves_odb12) for the 177 newly assembled genomes. Each bar represents an individual species, color-coded by gene category: complete single-copy, complete duplicated, fragmented, and missing.

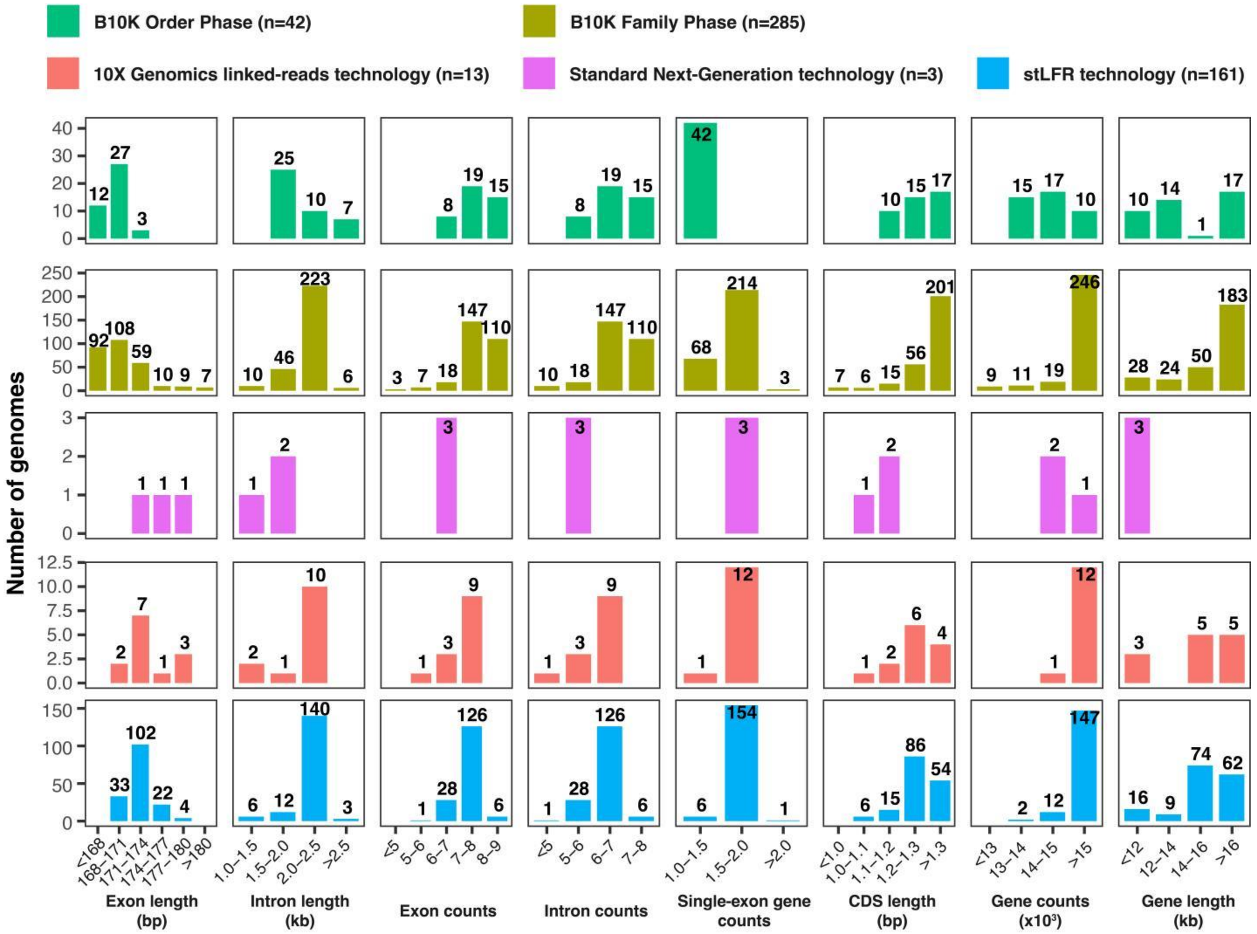

Figure 5. **Evaluation of gene annotation quality and consistency**. Distribution of eight primary annotation indicators for 504 avian genomes, stratified by B10K project phase and sequencing technology (stLFR, 10X Genomics, and NGS). From left to right, the panels display the frequency distributions of mean exon and intron lengths, mean numbers of exons and introns, single-exon gene counts, mean CDS lengths, total gene numbers, and mean gene lengths.

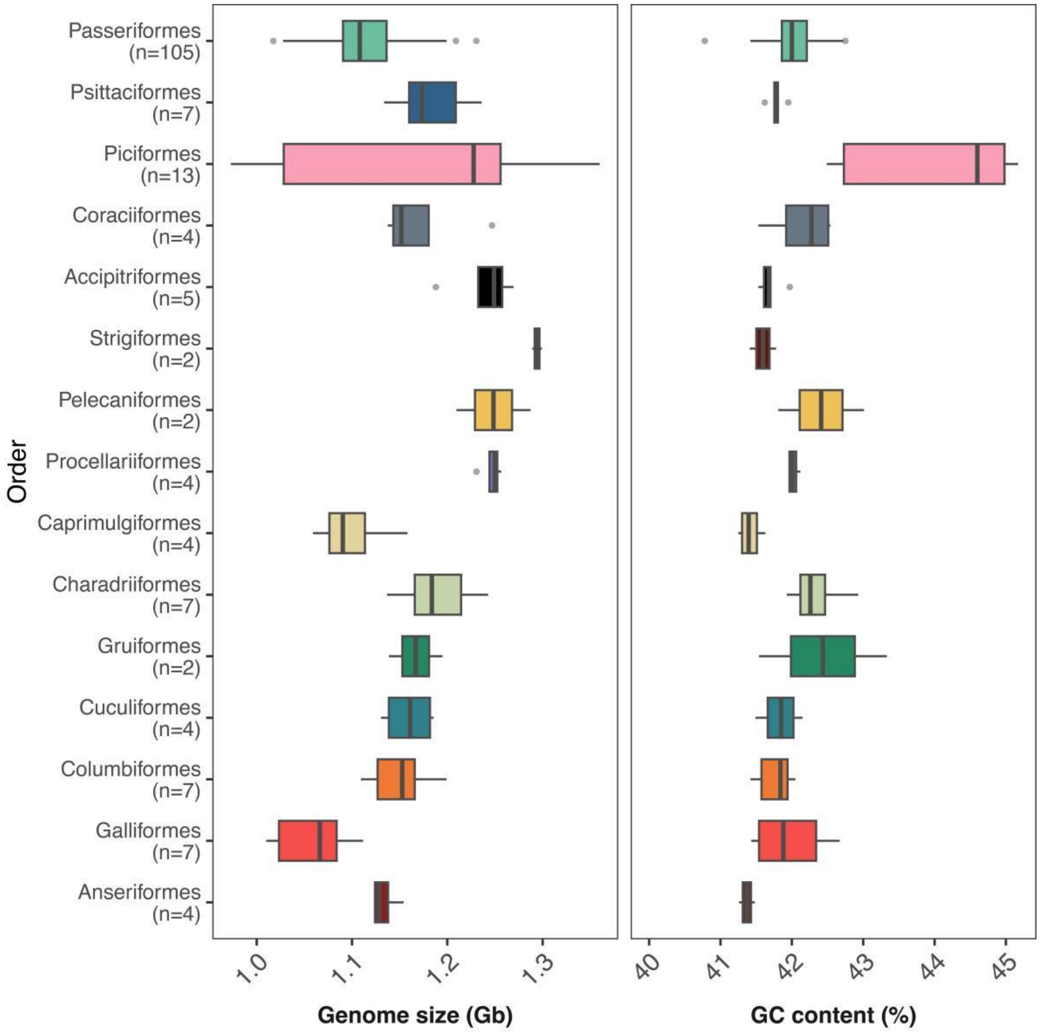

Figure 6. Comparative analysis of genome size and GC content across major avian taxonomic orders. Boxplots illustrating the distribution of estimated genome sizes (Gb) and GC content (%) for the 177 sequenced avian species, grouped by taxonomic order. The number of species (n) within each order is indicated in parentheses along the y-axis. For each boxplot, the thick vertical line represents the median, the box boundaries indicate the first and third quartiles (interquartile range, IQR), and the whiskers extend to the rest of the distribution, except for points that are determined to be outliers, which are plotted as individual gray dots.

Figure 1

Click here to access/download;Figure;Figure_1_

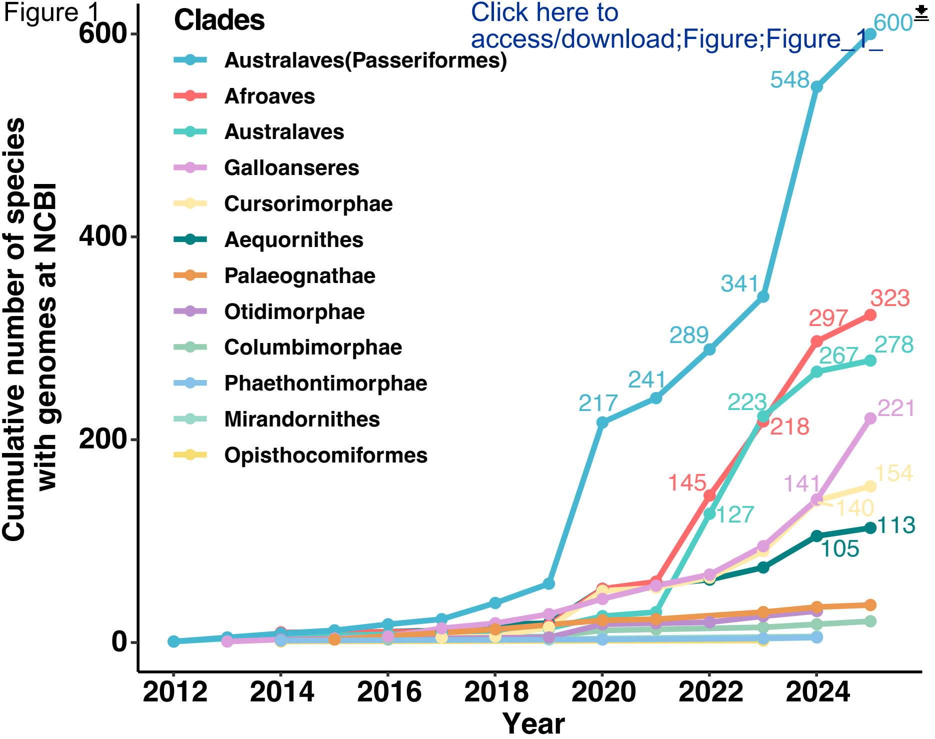

Figure 2

Click here to access/download;Figure;Figure_2_

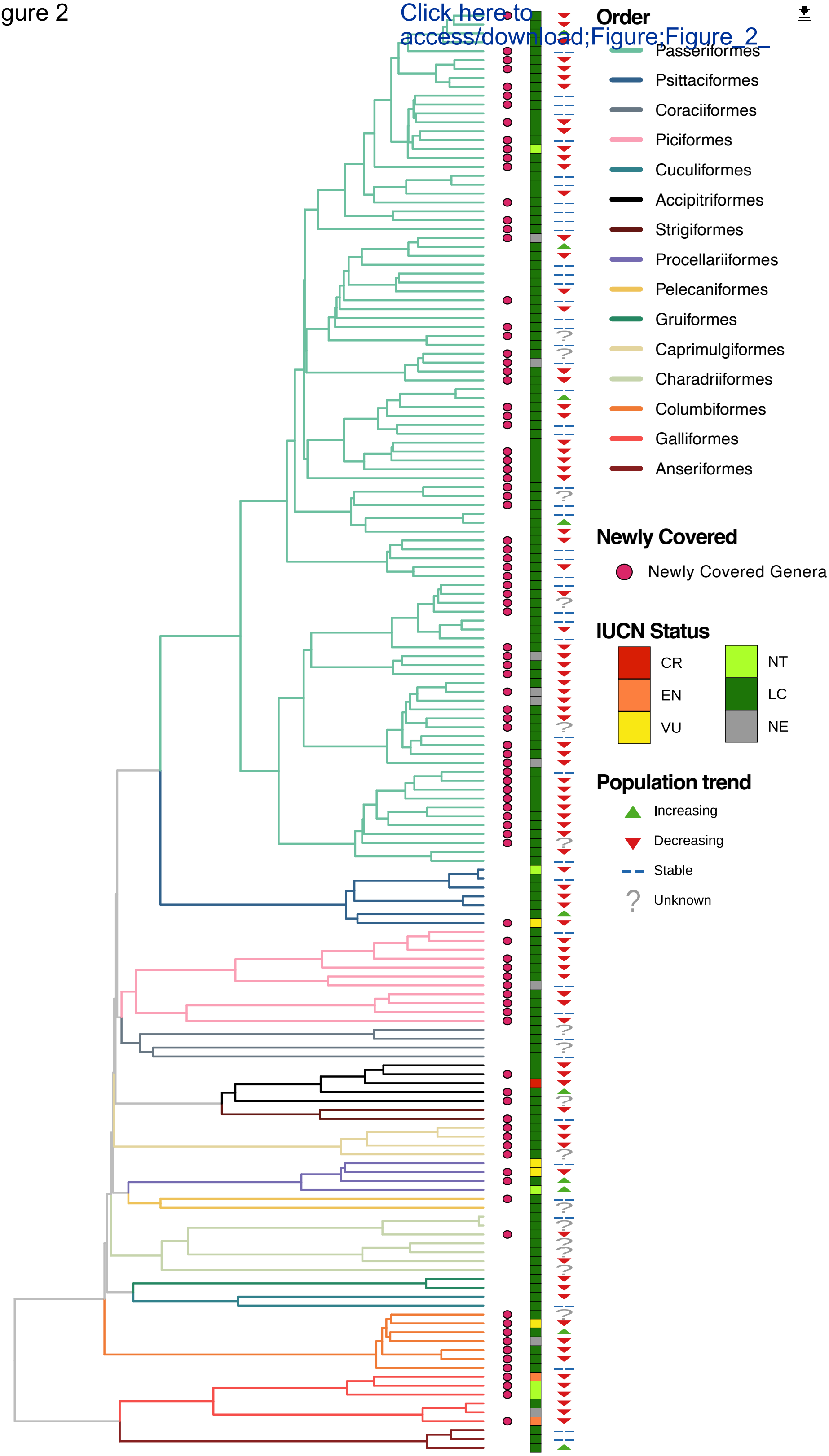

Figure 3

Click here to access/download;Figure;Figure

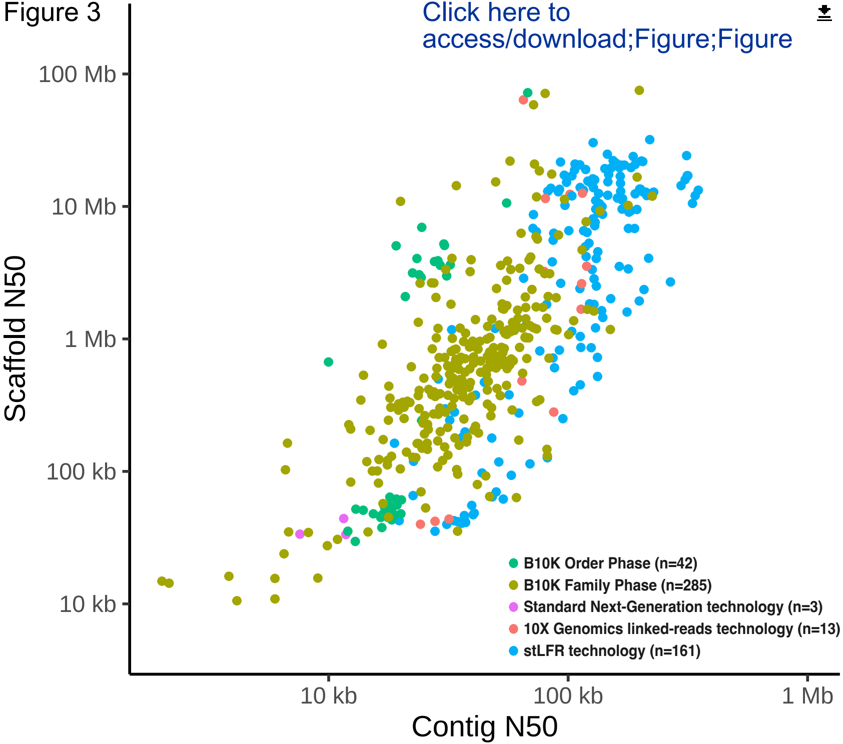

Figure 4

Click here to access/download;Figure;Figure_4_

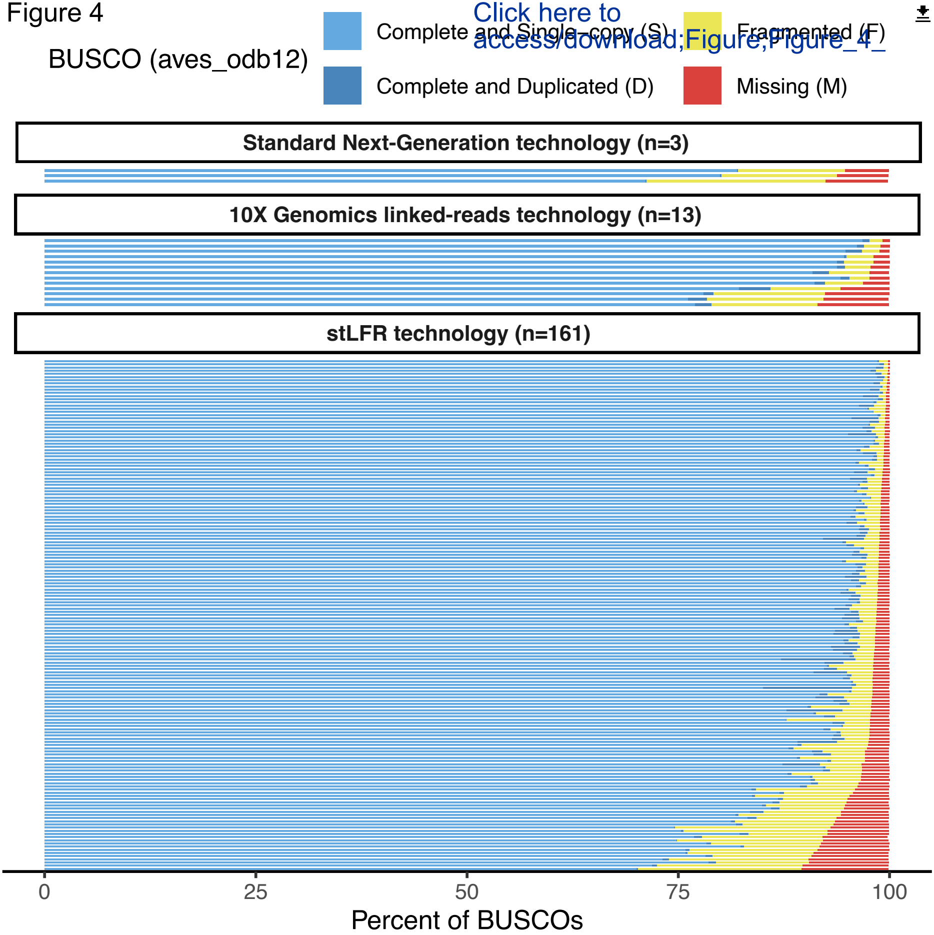

Figure 5

Click here to access/download;Figure;Figure_5_R1.pdf

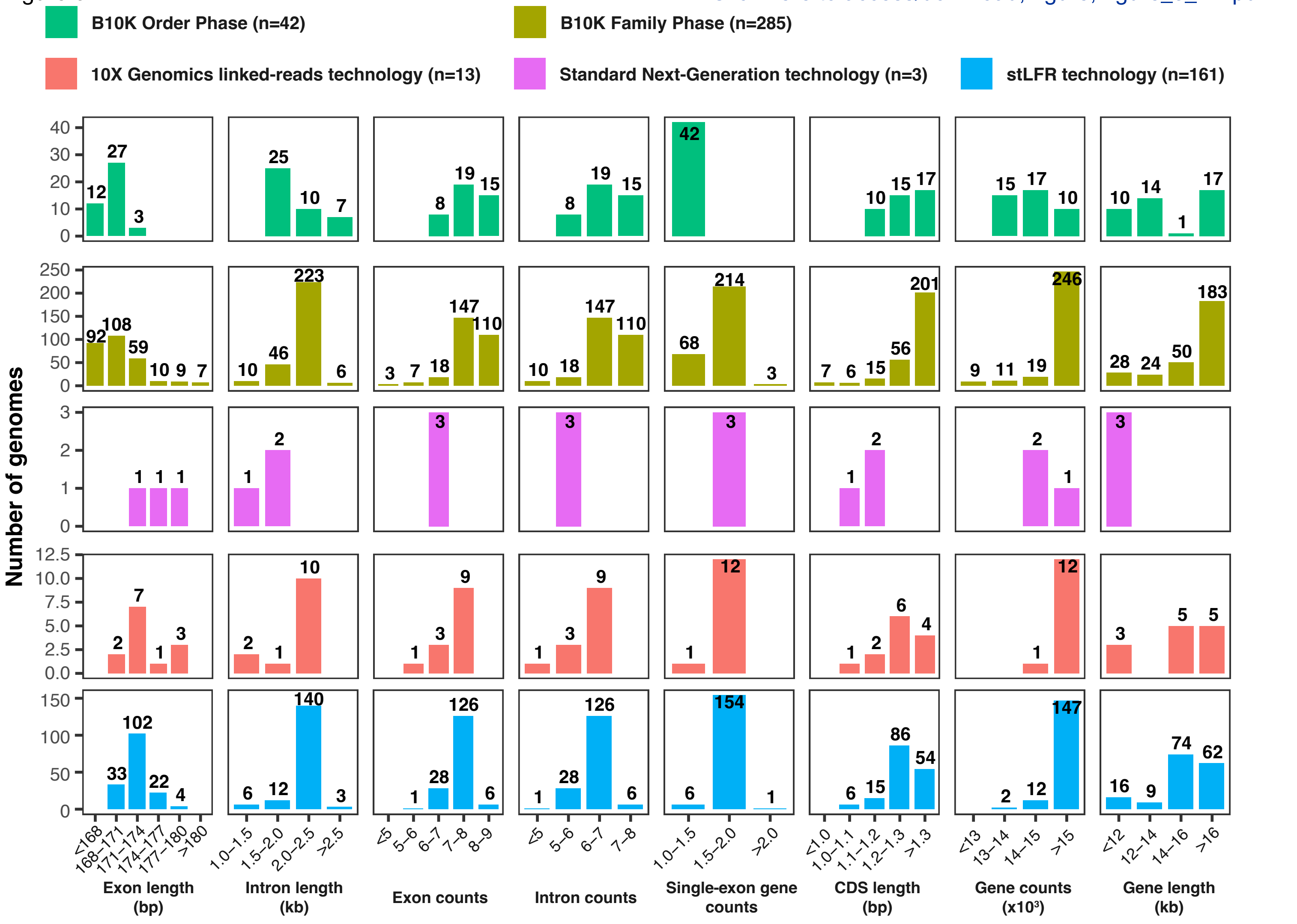

Figure 6

Click here to access/download;Figure;Figure_6_R1.pdf

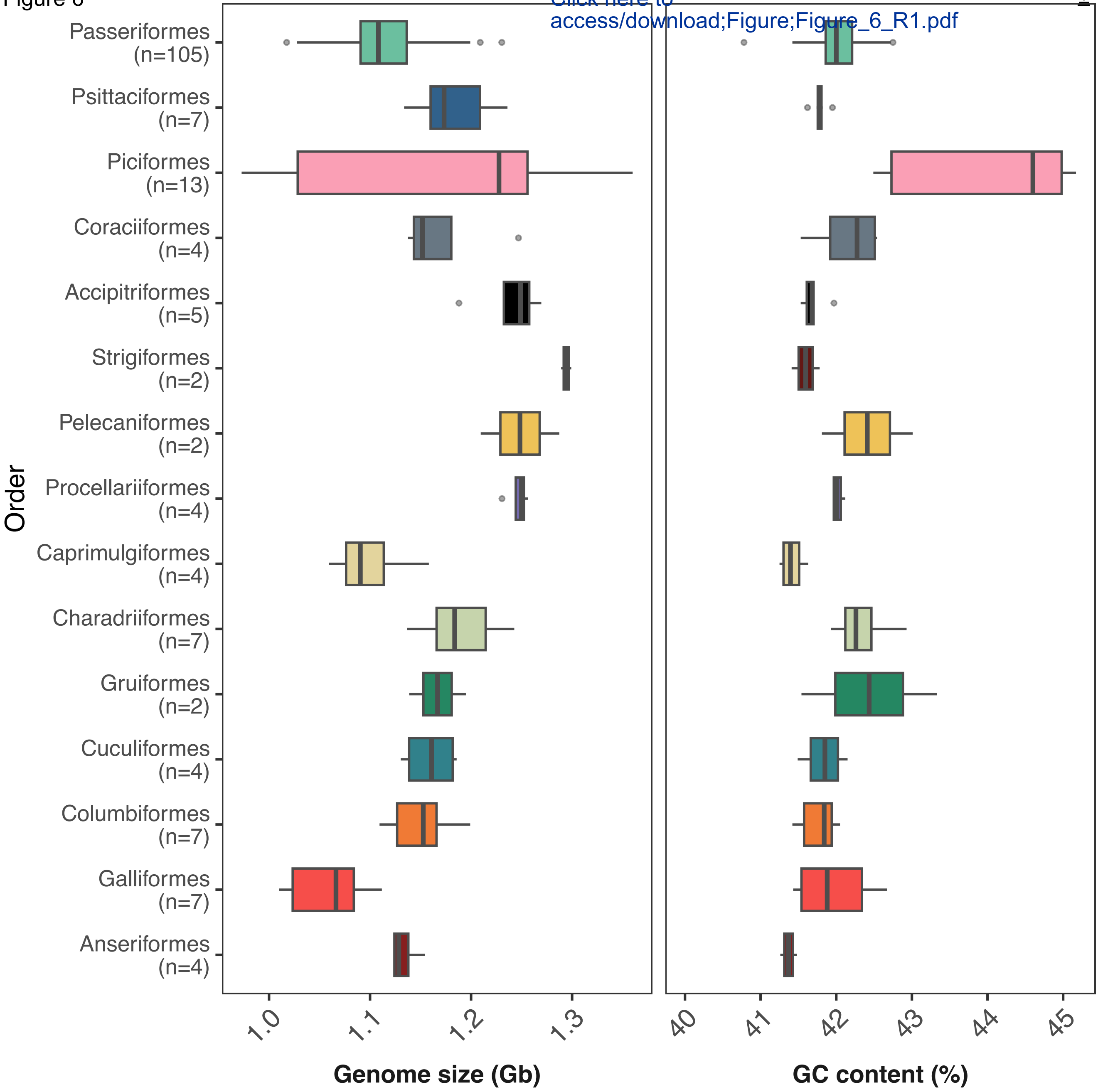

Supplementary Table 1

Click here to access/download
Supplementary Material
Supplementary Table 1.xlsx

Cover letter

Click here to access/download;Personal Cover;Cover Letter_R1.pdf

浙江大学生命演化研究中心
CENTRE FOR EVOLUTIONARY & ORGANISMAL BIOLOGY AT ZHEJIANG UNIVERSITY

ZHEJIANG UNIVERSITY
Centre for Evolutionary & Organismal Biology

School of Medicine, Zhejiang University
866# Yuhangtang Road, Xihu District
Hangzhou, Zhejiang, China
https://evolution.zju.edu.cn

Dear Editor,

We are pleased to submit the revision of the manuscript (GIGA-D-25-00475) entitled "Draft assemblies for 177 bird species enhance genus-level coverage" for consideration as a Data Note in GigaScience. We sincerely appreciate the constructive feedback from the editor and reviewers, which has significantly improved the quality and completeness of this work.

This manuscript presents 177 draft avian genome assemblies spanning 161 genera, including 102 genera with genomes sequenced for the first time at the genomic level. Leveraging primarily stLFR technology complemented by 10X Genomics and standard next-generation sequencing, we obtained draft genome assemblies from both frozen tissues and historical museum specimens, substantially augmenting genus-level taxonomic coverage within the avian phylogeny. These genomic resources provide a critical foundational dataset for comparative genomics, conservation genetics, phylogenomics, and evolutionary research.

In response to the reviewers' comments, we have made the following major revisions:

1. **Sequencing strategy rationale**: We have clarified the rationale for selecting different sequencing technologies (stLFR, 10X Genomics, and standard NGS) based on DNA integrity and the temporal availability of platforms, and added the relevant details in the Methods section.
2. **Metadata of Samples**: We have updated Supplementary Table 1 to include comprehensive sample metadata, including sample source type, collection time, location, specimen numbers, and the corresponding library type for each species.
3. **Assembly workflow reproducibility**: We have expanded the assembly workflow description with detailed tool versions, commands, key parameters, and the specific stlfr2supernova conversion pipeline.
4. **Contamination screening**: We have added the description of contamination screening using the NCBI FCS-GX pipeline and taxonomic verification via mitogenome BLAST and molecular markers.
5. **Additional quality metrics**: We have conducted Merqury-based k-mer evaluations and reported QV scores for all assemblies.
6. **Conservation relevance**: We have enhanced the discussion of conservation genetics applications, added IUCN status annotations to Figure 2, and improved all figure labels and legends.

A detailed point-by-point response to each reviewer's comment is provided in the accompanying "Response to Reviewers" document. All changes in the revised manuscript have been highlighted for the convenience of the editor and reviewers.

We believe that the revised manuscript has been substantially improved and now fully addresses all the concerns raised. We hope that the manuscript is now suitable for publication in GigaScience. We look forward to hearing from you at your earliest convenience.

Sincerely,

Guojie Zhang on behalf of all co-authors
Center of Evolutionary & Organismal Biology
Zhejiang University School of Medicine,
Hangzhou, 310058, China
E-mail: guojiezhang@zju.edu.cn
