# Supplementary material for: Draft assemblies for 177 bird species enhance genus-level coverage
Source: Gigascience. 2026 May 9;15:giag045. doi: 10.1093/gigascience/giag045 (PMC13274736; doi:10.1093/gigascience/giag045)
Supplement: giag045_Authors_Response_To_Reviewer_Comments_original_submission [file giag045_authors_response_to_reviewer_comments_original_submission.pdf]

Author's Response To Reviewer Comments

Response to Reviewers
Response: We would like to thank the editor and reviewers for taking the time to review our manuscript and providing constructive suggestions to improve this work. Below, we provide a point-by-point response to reviewers' comments.

Reviewer #1:
As a Data Note, this manuscript centers on the draft genome assemblies of 177 bird species, substantially augmenting the genus-level taxonomic coverage of avian genomes. Significantly, high-quality genomes were successfully acquired from museum specimens and tissue samples through st-LFR, addressing many gaps in avian genomic resources at the genus level. This study offers a critical foundational dataset for comparative genomics, conservation genetics, and evolutionary research. The research design is well-defined, the technical approach is logical, and the data volume is ample with high completeness and provided a valuable dataset for scientific field.
Response: We appreciate your time and effort on our manuscript. Your insightful comments are very useful for us to refine our manuscript. We have carefully read your comments and addressed them in this revision.

Several comments:
(1) The rationale for selecting three sequencing technologies (161 samples with st-LFR, 13 samples with 10X Genomics, and 3 samples with standard next-generation sequencing) is unclear (e.g., differences in sample quality, species-specific requirements, etc.). It is recommended to supplement the initial DNA quality parameters of different samples (such as DNA integrity values, degradation degrees), explain why some samples adopted 10X Genomics or standard next-generation sequencing, and whether the impact of this differentiated strategy on assembly quality has been evaluated. This details would help this field to have a general idea about the different sequencing methods adapted to different sample quality.
Response: We thank the reviewer for this suggestion. The differentiated sequencing strategy was indeed an adaptive approach based on both the temporal availability of technologies and the variations in DNA integrity across different samples. The 13 samples were sequenced during the early stages of the project when 10X Genomics was the leading technology for high-quality, long-range genome assembly. It was applied to samples with exceptional DNA integrity (fragment size >40kb). As stLFR technology matured and became the primary platform, it was adopted for the 161 samples due to its lower DNA input requirements (10ng with fragment size >10kb) while still providing high-quality linked-read information. And considering the preciousness of the samples, the remaining 3 samples were relatively degraded (fragment size <10kb) and did not meet the requirements for linked-read technologies, so we opted for standard next-generation sequencing to ensure at least basic genomic coverage. And we have added these information in the "Methods" section as following:
"The selection of sequencing technologies was strategically determined by the interplay between DNA integrity and the progressive availability of sequencing platforms throughout the project. The majority of the DNA samples (n = 161) were prepared using the stLFR (Single-Tube Long Fragment Reads) technology, which served as our primary pipeline due to its ability to generate high-quality linked-read data with 10 ng of input DNA with fragment sizes exceeding 10 kb. During the early phase of this study, 13 DNA samples which exhibited exceptional DNA integrity (main fragment sizes > 40 kb) were prepared using the 10X Genomics Chromium library system. For the remaining 3 samples with relative DNA degradation (fragment sizes < 10 kb) that failed to meet the requirements for linked-read library construction, standard short-read next-generation technology with approximately 500 ng input DNA was employed to ensure sufficient genomic coverage despite suboptimal sample quality."

(2) The reference gene set in homologous annotation includes core avian genes, human genes, and transcriptome data, but the rationale for using the human gene set for avian gene annotation is not explained (e.g., the risk of missing species-specific genes). Current several avian genomes and annotations can meet the T2T genome assemblies and annotations. It is recommended to supplement the screening criteria of the reference gene set and the contribution ratio of different reference sets to the annotation results (such as the proportion of genes annotated by core avian genes); meanwhile, the annotation of

non-coding RNAs is not mentioned. If no relevant analysis was conducted, it should be stated in the limitations; if it has been carried out, the relevant results need to be supplemented.
Response: We thank the reviewer for this comment regarding the reference gene sets. We agree that reliance on human data for avian annotation requires careful justification to avoid missing specific genes. To address the concern of potential bias, we have quantified the contribution of each reference set to the final gene models. Among the protein-coding annotations of these 177 avian species, on average, 89.53% by core avian genes set, 8.43% of genes supported by the transcriptome gene set, and 2.03% by human genes set. We have added the proportion of genes annotated by core avian genes into the Supplementary Table 1, and revised the related sentences in the "Gene structures annotation" and "Re-use potential" section.

We also acknowledge that the annotation of non-coding RNAs (ncRNAs) is a significant component of genome characterization. As suggested, we have clearly stated this in the "Re-use potential" section as following:
“Since our gene prediction primarily relies on a homology-based pipeline, there may be reduced sensitivity in identifying lineage-specific genes that lack close homologs in reference databases. Additionally, potential gene fragmentation effects may exist in regions where the assembly is less contiguous, which is a common trade-off in homology-based structural annotations. It should be noted that while this study provides the protein-coding gene annotations, the identification and characterization of non-coding RNAs (ncRNAs), such as miRNAs and lncRNAs, were not conducted in the current study. Future studies leveraging small RNA sequencing and specialized bioinformatic pipelines will be necessary to fully capture the regulatory landscape of these birds' genomes.”

(3) Only the average assembly and annotation indicators of the 177 genomes are presented, without grouping comparison by avian order or family-level taxonomic units, making it impossible to reflect the differences in genomic characteristics (such as genome size, gene number, intron length) among different avian groups. It is recommended to supplement grouped statistical analysis by taxonomic units to reveal the group-specific patterns of avian genomes.
Response: We thank the reviewer for this suggestion. In the revised manuscript, we have included a grouped statistical analysis of genome size and GC content across different taxonomic orders. Notably, our results show that bird species within the order Piciformes have a relatively higher average GC content of approximately 44% with larger genome size variation (Figure 6), which is consistent with previous avian genomic studies (Kapusta and Suh 2017).

(4) As a Data Note, in-depth functional analysis is not required, but combined with the conservation status of some species, the specific application scenarios of their genomic data in conservation genetics (such as population genetic structure analysis, genetic diversity assessment of endangered species) can be illustrated to enhance the application orientation of the data.
Response: We appreciate this suggestion. We agree that emphasizing the practical application of genomic data in conservation genetics will significantly enhance the value of this Data Note. As suggested, we have revised the "Re-use potential" section as following:
“While it will be useful for broad avian evolutionary studies, we expect its most significant value will be in conservation genetics and the practical management of the threatened species included. Specifically, these genomic resources enable the assessments of genetic diversity in endangered birds, providing essential data to identify inbreeding depression or genetic bottlenecks. Furthermore, the dataset facilitates precise population genetic structure analysis and the mapping of gene flow, which are critical for designing effective translocation or captive breeding programs.”

(5) It is recommended to mark the endangered species or endemic species can be marked in the figure to highlight the conservation value of the data if possible. The axis labels and legend information of Figures 3 and 5 are incomplete (e.g., some axes do not specify units, and abbreviations of indicator names are not annotated). It is recommended to standardize the figure annotations to ensure that readers can directly interpret the data.
Response: We have implemented the following improvements to enhance the clarity and impact of our data visualization. In Figure 2, we have explicitly marked IUCN Status and population trend according to the IUCN Red List. We also have thoroughly revised the axis labels, legends, and captions for Figures 3 and 5.

(6) The supplementary tables do not include detailed collection information of each sample (such as collection time, location, specimen number, preservation method) and specific parameters of DNA extraction (such as RNase A treatment time, bead purification conditions). This information is crucial for other researchers to repeat the experiment or verify the data. It is recommended to improve the supplementary tables and supplement the above key experimental details.
Response: We agree that detailed sample metadata and experimental parameters are essential for data reproducibility. We have addressed these concerns as suggested. We have updated the Supplementary Table 1 to include the collection details for each of the 177 species, including collection time, location, specimen/accession numbers, and sample types. We have revised the "DNA extraction" section to provide specific technical details as
“For post-lysis, RNA degradation was performed by incubating the lysate with 10 μL RNase A at 37°C for 5 minutes. Subsequently, genomic DNA was isolated using a bead-based purification method, utilizing 440 μL of DNA Binding Bead Mix per sample. The automated isolation process was executed on the Kingfisher Duo Prime system (Thermo Fisher Scientific), following the standard MMX_Ultra2_Cell_Tissue_96_Duo protocol.”

This manuscript successfully assembled the genomes of 177 bird species using low-input sequencing technologies, significantly enhancing the genus-level coverage of avian genomes. The data quality is reliable and the sharing is good, which meets the requirements for publishing Data Notes in GigaScience. It is recommended that the authors supplement the sample selection logic, technical details, and data completeness in response to the above issues, optimize the figure and code explanations. The manuscript can be considered for acceptance after revision.
Response: We appreciate your time and effort on our manuscript. We have carefully addressed your comments in this revision.

Reviewer #2:
Overall evaluation
This Data Note reports 177 draft avian genome assemblies spanning 161 genera, including 102 genera with genomes sequenced for the first time, and provides baseline quality metrics. The dataset substantially improves genus-level genome coverage in birds and represents a valuable community resource, particularly for comparative genomics, phylogenomics, and biodiversity/conservation applications.
The work is timely and impactful. The manuscript is generally clear and well-structured, and the resource value is high. However, as a Data Note, the strongest requirement is reusability and reproducibility. I recommend a minor revision to strengthen transparency of sample provenance, reproducibility of the assembly workflow, and contamination screening/validation.
Response: We appreciate the reviewer's interest in our work. We have revised the manuscript accordingly according to the constructive and insightful comments of the reviewer.

Major comments
1) Clarify sample provenance (museum vs fresh tissue/blood) and dataset composition
The manuscript emphasizes the utility of low-input strategies and mentions museum specimens, but it is not fully clear what proportion of the 177 genomes were derived from challenging sources (e.g., historic toepads) versus fresh tissues or blood. For re-use and interpretation, the manuscript would benefit from a concise summary of sample types.
Requested improvement :
Add a short table or summary paragraph specifying, for all species:
sample source type (museum toepad/historical vs fresh/frozen tissue vs blood)
preservation condition (if known)
library type per sample (st-LFR / 10X / short-read NGS)
Response: We agree that clarifying the provenance and type of samples is essential for interpreting the assembly quality and the utility of our low-input strategies. As requested, we have provided a comprehensive breakdown of the sample metadata. We have updated the Supplementary Table 1 to include specific columns for sample source type (e.g., skin tissue, muscle tissue, or blood sample), and the corresponding library type (stLFR, 10X, or NGS) for each of the 177 species. We also noted that 80.8% of the samples (143 out of 177) were collected prior to 2020 in the revised manuscript.

2) Assembly workflow needs more parameter detail for reproducibility

The assembly workflow is described at a high level (st-LFR conversion to 10X format; Supernova pseudohap; Gapcloser), but critical details are missing for others to reproduce the pipeline.
Requested improvement:
Please add the main tools/commands and key parameters, including:
name/version of the tool for st-LFR → 10X conversion, and the essential settings
any major Supernova settings beyond pseudohap mode
Gapcloser parameters
clarify at what stage and how many scaffolds were removed with the "N > 80%" filtering criterion
Response: We thank the reviewer for emphasizing the importance of reproducibility in assembly workflow. In the revised manuscript, we have expanded the sentences to include comprehensive technical details and specific parameters. We have explicitly stated the use of the stlfr2supernova pipeline (https://github.com/BGI-Qingdao/stlfr2supernova_pipeline) for format conversion and added the command line for Supernova (v2.0.1). We also clarified the scaffold filtering process based on "N" content, which resulted in the removal of an average of 2 scaffolds per assembly. Furthermore, we revised the sentences related to GapCloser (v1.12) using the default parameters and the cleaned paired-end reads for gap filling.

3) Add contamination screening / taxonomic validation summary
Species validation is performed using BLAST of mitochondrial genomes (and nuclear markers when mitochondria are unavailable). This is useful, but for large datasets—especially those potentially involving museum/historic material—contamination screening is important.
Requested improvement :
Provide a short contamination screening result summary using a standard approach (e.g., Kraken2/Centrifuge summary, BlobToolKit, or similar), even if only for a subset, OR
Include a statement of what contamination checks were done and thresholds used
Response: We agree that contamination screening and taxonomic validation are critical for large-scale genomic datasets. We have now included a detailed summary of our quality control pipeline in the revised manuscript under the "Contamination screening" and "Sample taxonomic verification" sections. Specifically, we employed the NCBI Foreign Contamination Screen (FCS-GX) pipeline to systematically detect and remove adapter sequences and non-avian contaminant scaffolds (e.g., bacteria, fungi, or human DNA) using default sensitivity thresholds. Additionally, we implemented a taxonomic verification strategy using mitogenome BLAST searches against the NCBI reference database, supplemented by specific mitochondrial markers (COI, ND2, CYB) or a suite of conserved avian nuclear markers (e.g., RAG1, GAPDH, FGB) to ensure both assembly purity and the accuracy of species identification.

4) Include at least one additional assembly integrity metric (optional but strongly recommended)
N50 and BUSCO are informative, but many users will expect at least one additional metric that reflects base-level accuracy or k-mer completeness.
Requested improvement:
If raw reads are available, please consider reporting Merqury QV/k-mer completeness (even for a representative subset). If this is not feasible, a short justification would be acceptable.
Response: We thank the reviewer for this suggestion, which has further strengthened the quality assessment of our dataset. As requested, we have conducted a k-mer-based evaluation using Merqury for our assemblies, which resulted in an average Merqury QV score of ~56. We have also incorporated these results into the revised manuscript and added the specific details for individual species to the Supplementary Table 1.

5) Gene annotation section: clarify limitations of purely homology-based prediction
Annotation is performed using a homology-based pipeline (TBLASTN → genBlastA → GeneWise). This is reasonable for a Data Note, but users should be warned about limitations such as reduced sensitivity for lineage-specific genes and potential fragmentation effects.
Requested improvement:
Add 2-3 sentences in the "Re-use potential" section clarifying expected limitations (e.g., no RNA-seq support, structural annotations mainly, assembly fragmentation effects).
Response: We have revised the "Re-use potential" section to explicitly clarify the limitations of our homology-based annotation pipeline. As suggested, we added sentences discussing the potential reduced sensitivity for lineage-specific genes and possible gene fragmentation effects associated with structural annotations. We believe these additions provide a more transparent and realistic guide for future users of

our dataset.

Minor comments
1. Please correct minor typos/formatting inconsistencies (e.g., figure label spelling and consistent "st-LFR" naming).
Response: We have revised the figure labels and consistent the naming as “stLFR” as Reviewer 3 mentioned.

2. Expand read preprocessing description slightly (adapter trimming, barcode handling, trimming thresholds) to improve clarity.
Response: We appreciate the reviewer's request for clarity on read preprocessing. While the "Quality Control" section provided a general overview, we have now explicitly integrated the technical parameters and filtering thresholds into the "Assembly" section to ensure full transparency and reproducibility as following:
“For the 161 samples sequenced using stLFR technology, raw data were initially transformed into 10X Genomics linked-reads format following the stlfr2supernova pipeline (https://github.com/BGI-Qingdao/stlfr2supernova_pipeline). Subsequently, these 161 samples, together with 13 samples sequenced by 10X Genomics linked-reads technology, were preprocessed with SOAPfilter (v2.2.1) using parameters `-i 500 -p -z -g 1 -M 2 -Q 10 lane.lst stat_file` to remove adapters and low-quality reads.”

3. Add one or two sentences interpreting the key figures showing genus-level coverage improvement across avian orders/families.
Response: We have added a sentence to the manuscript interpreting the significance of the improved genus-level coverage. The 102 newly sequenced genera effectively fill previous gaps in the avian genomic map, and an increase of 4.43% in total genus coverage significantly enhances the resolution of the avian tree of life, providing a more robust foundation for exploring evolutionary traits across diverse lineages.

Reviewer #3:
In this paper, the B10K Project reports the adoption of low-input sequencing strategies to reduce costs while improving assembly quality compared with their earlier generation of order- and family-level avian genomes. Using primarily stLFR, complemented by 10X Genomics, the authors report the generation of 177 avian genomes representing 161 genera, including 102 newly sequenced at the genomic level. These genomes will be of broad use to the research community. The paper is well written and represents a significant data contribution. I have a few minor suggestions for the authors to consider.
Response: We appreciate your professional review work on our manuscript. We have carefully read your comments and addressed them in this revision.

Sampling and DNA extraction
From the Introduction, I understood that genomes were being generated primarily from historical sources (e.g., toe pads), but the mention of tissue and blood here suggests I may have misunderstood. Are you using the stLFR method for samples with limited amounts of high-molecular-weight DNA? It would be useful to clarify the source material used for DNA extraction.
Response: We apologize for any confusion. To clarify, our dataset is composed of mostly frozen materials and historical museum specimens. If properly preserved, we can still obtain relatively high-quality DNA from these samples (main fragments size >20k). We chose the stLFR method not only for its ability to generate long-range genomic information from HMW DNA but also because of its low-input requirement. We revised the manuscript and added the tissue type and library construction method to each sample in the Supplementary Table 1.

Library construction
Please provide a citation for the stLFR method. In addition, please elaborate on the 100 + 100 + 42 bp paired-end reads. Are these three separate runs on the DNBSEQ-T7 platform?
Response: We have added the citation for stLFR technology (Wang et al., 2019) to the manuscript. And the 100 + 100 + 42 bp format does not represent three separate runs. Instead, it is a single, integrated paired-end sequencing run performed on the DNBSEQ-T7 platform. In this configuration, the first two 100 bp segments sequence the paired-end genomic inserts (Read 1 and Read 2), while the additional 42 bp

segment is a dedicated read designed to capture the co-barcoding information essential for stLFR long-fragment reconstruction.

stLFR and 10X Genomics
Please provide additional information on how the stLFR data were transformed into 10X Genomics format to enable assembly. If possible, could the script used for this conversion be deposited on GitHub or a similar repository to allow others to adopt a similar strategy?
Response: We have revised the sentences to include the technical details and specific parameters. We have explicitly stated the use of the stlfr2supernova pipeline (https://github.com/BGI-Qingdao/stlfr2supernova_pipeline) for format conversion.

Gene structures and annotation
In the first line of this section, something appears to be missing (e.g., "…homology-based method for both the ???…").
Response: Thank you for pointing this out. We have corrected the typo in the revised manuscript.

The genome annotation strategy is very useful and will further enhance the value of these genomes. To enable other researchers to replicate these analyses and apply similar approaches to their own genomes, could the primary reference gene set (20,194 genes), the supplemental human gene set (20,169 genes), and the supplemental transcriptome gene set (5,257 transcripts) be made available on GitHub or a similar data archive?
Response: We agree that making these gene sets accessible is crucial for the reproducibility of our analyses and for the broader research community. The primary reference gene set (20,194 genes), the supplemental human gene set (20,169 genes), and the supplemental transcriptome gene set (5,257 transcripts) can be accessed and downloaded via our GitHub repository via (https://github.com/B10KGenomes/annotation/tree/master/Gene_Annotation/0.homolog_data). And, we also have explicitly added this link in the relevant section to ensure easy navigation for future users.

Editorial Manager®

Cookie Preference Center

We use cookies which are necessary to make our site work. We may also use additional cookies to analyze, improve and personalize our content and your digital experience. For more information, see our Cookie Policy.

You may choose not to allow some types of cookies. However, blocking some types may impact your experience of our site and the services we are able to offer. See the different category headings below to find out more or change your settings.

Allow all

Manage Consent Preferences

Strictly Necessary Cookies

Always active

These cookies are necessary for the website to function and cannot be switched off in our systems. They are usually only set in response to actions made by you which amount to a request for services, such as setting your privacy preferences, logging in or filling in forms. You can set your browser to block or alert you about these cookies, but some parts of the site will not then work.

Functional Cookies

These cookies enable the website to provide enhanced functionality and personalisation. They may be set by us or by third party providers whose services we have added to our pages. If you do not allow these cookies then some or all of these services may not function properly.

Performance Cookies

These cookies allow us to count visits and traffic sources so we can measure and improve the performance of our site. They help us to know which pages are the most and least popular and see how visitors move around the site.

Targeting Cookies

These cookies may be set through our site by our advertising partners. They may be used by those companies to build a profile of your interests and show you relevant adverts on other sites. If you do not allow these cookies, you will experience less targeted advertising.

Cookie List

Clear

Apply Cancel

Consent Leg.Interest

Confirm my choices

Powe
