# Supplementary material for: Draft assemblies for 177 bird species enhance genus-level coverage
Source: Gigascience. 2026 May 9;15:giag045. doi: 10.1093/gigascience/giag045 (PMC13274736; doi:10.1093/gigascience/giag045)
Supplement: giag045_Reviewer_1_Report_original_submission — Reviewer 1 -- 12/21/2025 [file giag045_reviewer_1_report_original_submission.pdf]

Reviewer Report

Title: Draft assemblies for 177 bird species enhance genus-level coverage

Version: Original Submission **Date:** 12/21/2025

Reviewer name: Reviewer 1

Reviewer Comments to Author:

As a Data Note, this manuscript centers on the draft genome assemblies of 177 bird species, substantially augmenting the genus-level taxonomic coverage of avian genomes. Significantly, high-quality genomes were successfully acquired from museum specimens and tissue samples through st-LFR, addressing many gaps in avian genomic resources at the genus level. This study offers a critical foundational dataset for comparative genomics, conservation genetics, and evolutionary research. The research design is well-defined, the technical approach is logical, and the data volume is ample with high completeness and provided a valuable dataset for scientific field.Several comments:(1) The rationale for selecting three sequencing technologies (161 samples with st-LFR, 13 samples with 10X Genomics, and 3 samples with standard next-generation sequencing) is unclear (e.g., differences in sample quality, species-specific requirements, etc.). It is recommended to supplement the initial DNA quality parameters of different samples (such as DNA integrity values, degradation degrees), explain why some samples adopted 10X Genomics or standard next-generation sequencing, and whether the impact of this differentiated strategy on assembly quality has been evaluated. This details would help this field to have a general idea about the different sequencing methods adapted to different sample quality.(2) The reference gene set in homologous annotation includes core avian genes, human genes, and transcriptome data, but the rationale for using the human gene set for avian gene annotation is not explained (e.g., the risk of missing species-specific genes). Current several avian genomes and annotations can meet the T2T genome assemblies and annotations. It is recommended to supplement the screening criteria of the reference gene set and the contribution ratio of different reference sets to the annotation results (such as the proportion of genes annotated by core avian genes); meanwhile, the annotation of non-coding RNAs is not mentioned. If no relevant analysis was conducted, it should be stated in the limitations; if it has been carried out, the relevant results need to be supplemented.(3) Only the average assembly and annotation indicators of the 177 genomes are presented, without grouping comparison by avian order or family-level taxonomic units, making it impossible to reflect the differences in genomic characteristics (such as genome size, gene number, intron length) among different avian groups. It is recommended to supplement grouped statistical analysis by taxonomic units to reveal the group-specific patterns of avian genomes.(4) As a Data Note, in-depth functional analysis is not required, but combined with the conservation status of some species, the specific application scenarios of their genomic data in conservation genetics (such as population genetic structure analysis, genetic diversity assessment of endangered species) can be illustrated to enhance the application orientation of the data.(5) It is recommended to mark the endangered species or endemic species can be marked in the figure to highlight the conservation value of the data if possible. The axis labels and legend information of Figures 3 and 5 are incomplete (e.g., some axes do not specify units, and

abbreviations of indicator names are not annotated). It is recommended to standardize the figure annotations to ensure that readers can directly interpret the data.(6) The supplementary tables do not include detailed collection information of each sample (such as collection time, location, specimen number, preservation method) and specific parameters of DNA extraction (such as RNase A treatment time, bead purification conditions). This information is crucial for other researchers to repeat the experiment or verify the data. It is recommended to improve the supplementary tables and supplement the above key experimental details.This manuscript successfully assembled the genomes of 177 bird species using low-input sequencing technologies, significantly enhancing the genus-level coverage of avian genomes. The data quality is reliable and the sharing is good, which meets the requirements for publishing Data Notes in GigaScience. It is recommended that the authors supplement the sample selection logic, technical details, and data completeness in response to the above issues, optimize the figure and code explanations. The manuscript can be considered for acceptance after revision.

Level of Interest

Please indicate how interesting you found the manuscript: Choose an item.

Quality of Written English

Please indicate the quality of language in the manuscript: Choose an item.

Declaration of Competing Interests

Please complete a declaration of competing interests, considering the following questions:

- Have you in the past five years received reimbursements, fees, funding, or salary from an organisation that may in any way gain or lose financially from the publication of this manuscript, either now or in the future?
- Do you hold any stocks or shares in an organisation that may in any way gain or lose financially from the publication of this manuscript, either now or in the future?
- Do you hold or are you currently applying for any patents relating to the content of the manuscript?
- Have you received reimbursements, fees, funding, or salary from an organization that holds or has applied for patents relating to the content of the manuscript?
- Do you have any other financial competing interests?
- Do you have any non-financial competing interests in relation to this paper?

If you can answer no to all of the above, write 'I declare that I have no competing interests' below. If your reply is yes to any, please give details below.

I declare that I have no competing interests

I agree to the open peer review policy of the journal. I understand that my name will be included on my report to the authors and, if the manuscript is accepted for publication, my named report including any attachments I upload will be posted on the website along with the authors' responses. I agree for my report to be made available under an Open Access Creative Commons CC-BY license (http://creativecommons.org/licenses/by/4.0/). I understand that any comments which I do not wish to be included in my named report can be included as confidential comments to the editors, which will not be published.

Choose an item.

To further support our reviewers, we have joined with Publons, where you can gain additional credit to further highlight your hard work (see: https://publons.com/journal/530/gigascience). On publication of this paper, your review will be automatically added to Publons, you can then choose whether or not to claim your Publons credit. I understand this statement.

Yes Choose an item.
