# Supplementary material for: Draft assemblies for 177 bird species enhance genus-level coverage
Source: Gigascience. 2026 May 9;15:giag045. doi: 10.1093/gigascience/giag045 (PMC13274736; doi:10.1093/gigascience/giag045)
Supplement: giag045_Reviewer_2_Report_original_submission — Reviewer 2 -- 1/17/2026 [file giag045_reviewer_2_report_original_submission.pdf]

Reviewer Report

Title: Draft assemblies for 177 bird species enhance genus-level coverage

Version: Original Submission **Date:** 1/17/2026

Reviewer name: Reviewer 2

Reviewer Comments to Author:

Reviewer ReportManuscript: Draft assemblies for 177 bird species enhance genus-level coverageID: GIGA-D-25-00475Overall evaluationThis Data Note reports 177 draft avian genome assemblies spanning 161 genera, including 102 genera with genomes sequenced for the first time, and provides baseline quality metrics. The dataset substantially improves genus-level genome coverage in birds and represents a valuable community resource, particularly for comparative genomics, phylogenomics, and biodiversity/conservation applications.The work is timely and impactful. The manuscript is generally clear and well-structured, and the resource value is high. However, as a Data Note, the strongest requirement is reusability and reproducibility. I recommend a minor revision to strengthen transparency of sample provenance, reproducibility of the assembly workflow, and contamination screening/validation.Major comments1) Clarify sample provenance (museum vs fresh tissue/blood) and dataset compositionThe manuscript emphasizes the utility of low-input strategies and mentions museum specimens, but it is not fully clear what proportion of the 177 genomes were derived from challenging sources (e.g., historic toepads) versus fresh tissues or blood. For re-use and interpretation, the manuscript would benefit from a concise summary of sample types.Requested improvement :Add a short table or summary paragraph specifying, for all species:sample source type (museum toepad/historical vs fresh/frozen tissue vs blood)preservation condition (if known)library type per sample (st-LFR / 10X / short-read NGS)2) Assembly workflow needs more parameter detail for reproducibilityThe assembly workflow is described at a high level (st-LFR conversion to 10X format; Supernova pseudohap; Gapcloser), but critical details are missing for others to reproduce the pipeline.Requested improvement:Please add the main tools/commands and key parameters, including:name/version of the tool for st-LFR â†’ 10X conversion, and the essential settingsany major Supernova settings beyond pseudohap modeGapcloser parametersclarify at what stage and how many scaffolds were removed with the "N > 80%" filtering criterion3) Add contamination screening / taxonomic validation summarySpecies validation is performed using BLAST of mitochondrial genomes (and nuclear markers when mitochondria are unavailable). This is useful, but for large datasetsâ€”especially those potentially involving museum/historic materialâ€”contamination screening is important.Requested improvement :Provide a short contamination screening result summary using a standard approach (e.g., Kraken2/Centrifuge summary, BlobToolKit, or similar), even if only for a subset, ORInclude a statement of what contamination checks were done and thresholds used4) Include at least one additional assembly integrity metric (optional but strongly recommended)N50 and BUSCO are informative, but many users will expect at least one additional metric that reflects base-level accuracy or k-mer completeness.Requested improvement:If raw reads are available, please consider reporting Merqury QV/k-mer completeness (even for a representative subset). If this is not feasible, a short

justification would be acceptable.5) Gene annotation section: clarify limitations of purely homology-based predictionAnnotation is performed using a homology-based pipeline (TBLASTN â†’ genBlastA â†’ GeneWise). This is reasonable for a Data Note, but users should be warned about limitations such as reduced sensitivity for lineage-specific genes and potential fragmentation effects.Requested improvement:Add 2-3 sentences in the "Re-use potential" section clarifying expected limitations (e.g., no RNA-seq support, structural annotations mainly, assembly fragmentation effects).Minor commentsPlease correct minor typos/formatting inconsistencies (e.g., figure label spelling and consistent "st-LFR" naming).Expand read preprocessing description slightly (adapter trimming, barcode handling, trimming thresholds) to improve clarity.Add one or two sentences interpreting the key figures showing genus-level coverage improvement across avian orders/families.

Level of Interest

Please indicate how interesting you found the manuscript: Choose an item.

Quality of Written English

Please indicate the quality of language in the manuscript: Choose an item.

Declaration of Competing Interests

Please complete a declaration of competing interests, considering the following questions:

- Have you in the past five years received reimbursements, fees, funding, or salary from an organisation that may in any way gain or lose financially from the publication of this manuscript, either now or in the future?
- Do you hold any stocks or shares in an organisation that may in any way gain or lose financially from the publication of this manuscript, either now or in the future?
- Do you hold or are you currently applying for any patents relating to the content of the manuscript?
- Have you received reimbursements, fees, funding, or salary from an organization that holds or has applied for patents relating to the content of the manuscript?
- Do you have any other financial competing interests?
- Do you have any non-financial competing interests in relation to this paper?

If you can answer no to all of the above, write 'I declare that I have no competing interests' below. If your reply is yes to any, please give details below.

I declare that I have no competing interests

I agree to the open peer review policy of the journal. I understand that my name will be included on my report to the authors and, if the manuscript is accepted for publication, my named report including any

attachments I upload will be posted on the website along with the authors' responses. I agree for my report to be made available under an Open Access Creative Commons CC-BY license (http://creativecommons.org/licenses/by/4.0/). I understand that any comments which I do not wish to be included in my named report can be included as confidential comments to the editors, which will not be published.

Choose an item.

To further support our reviewers, we have joined with Publons, where you can gain additional credit to further highlight your hard work (see: https://publons.com/journal/530/gigascience). On publication of this paper, your review will be automatically added to Publons, you can then choose whether or not to claim your Publons credit. I understand this statement.

Yes Choose an item.
