# Supplementary material for: Draft assemblies for 177 bird species enhance genus-level coverage
Source: Gigascience. 2026 May 9;15:giag045. doi: 10.1093/gigascience/giag045 (PMC13274736; doi:10.1093/gigascience/giag045)
Supplement: giag045_Reviewer_3_Report_original_submission — Reviewer 3 -- 2/12/2026 [file giag045_reviewer_3_report_original_submission.pdf]

Reviewer Report

Title: Draft assemblies for 177 bird species enhance genus-level coverage

Version: Original Submission **Date:** 2/12/2026

Reviewer name: Reviewer 3

Reviewer Comments to Author:

In this paper, the B10K Project reports the adoption of low-input sequencing strategies to reduce costs while improving assembly quality compared with their earlier generation of order- and family-level avian genomes. Using primarily stLFR, complemented by 10X Genomics, the authors report the generation of 177 avian genomes representing 161 genera, including 102 newly sequenced at the genomic level. These genomes will be of broad use to the research community. The paper is well written and represents a significant data contribution. I have a few minor suggestions for the authors to consider.Sampling and DNA extractionFrom the Introduction, I understood that genomes were being generated primarily from historical sources (e.g., toe pads), but the mention of tissue and blood here suggests I may have misunderstood. Are you using the stLFR method for samples with limited amounts of high-molecular-weight DNA? It would be useful to clarify the source material used for DNA extraction.Library constructionPlease provide a citation for the stLFR method. In addition, please elaborate on the 100 + 100 + 42 bp paired-end reads. Are these three separate runs on the DNBSEQ-T7 platform?stLFR and 10X GenomicsPlease provide additional information on how the stLFR data were transformed into 10X Genomics format to enable assembly. If possible, could the script used for this conversion be deposited on GitHub or a similar repository to allow others to adopt a similar strategy?Gene structures and annotationIn the first line of this section, something appears to be missing (e.g., "…homology-based method for both the ???…").The genome annotation strategy is very useful and will further enhance the value of these genomes. To enable other researchers to replicate these analyses and apply similar approaches to their own genomes, could the primary reference gene set (20,194 genes), the supplemental human gene set (20,169 genes), and the supplemental transcriptome gene set (5,257 transcripts) be made available on GitHub or a similar data archive?

Level of Interest

Please indicate how interesting you found the manuscript: Choose an item.

Quality of Written English

Please indicate the quality of language in the manuscript: Choose an item.

Declaration of Competing Interests

Please complete a declaration of competing interests, considering the following questions:

- Have you in the past five years received reimbursements, fees, funding, or salary from an organisation that may in any way gain or lose financially from the publication of this manuscript, either now or in the future?
- Do you hold any stocks or shares in an organisation that may in any way gain or lose financially from the publication of this manuscript, either now or in the future?
- Do you hold or are you currently applying for any patents relating to the content of the manuscript?
- Have you received reimbursements, fees, funding, or salary from an organization that holds or has applied for patents relating to the content of the manuscript?
- Do you have any other financial competing interests?
- Do you have any non-financial competing interests in relation to this paper?

If you can answer no to all of the above, write 'I declare that I have no competing interests' below. If your reply is yes to any, please give details below.

None.

I agree to the open peer review policy of the journal. I understand that my name will be included on my report to the authors and, if the manuscript is accepted for publication, my named report including any attachments I upload will be posted on the website along with the authors' responses. I agree for my report to be made available under an Open Access Creative Commons CC-BY license (http://creativecommons.org/licenses/by/4.0/). I understand that any comments which I do not wish to be included in my named report can be included as confidential comments to the editors, which will not be published.

I agree to the open peer review policy of the journal

To further support our reviewers, we have joined with Publons, where you can gain additional credit to further highlight your hard work (see: https://publons.com/journal/530/gigascience). On publication of this paper, your review will be automatically added to Publons, you can then choose whether or not to claim your Publons credit. I understand this statement.

Yes Choose an item.
